# Supplementary figures and images for: Peptide-based NTA(Ni)-nanodiscs for studying membrane enhanced FGFR1 kinase activities
Source: PeerJ. 2019 Jul 23;7:e7234. doi: 10.7717/peerj.7234 (PMC6659669; doi:10.7717/peerj.7234)

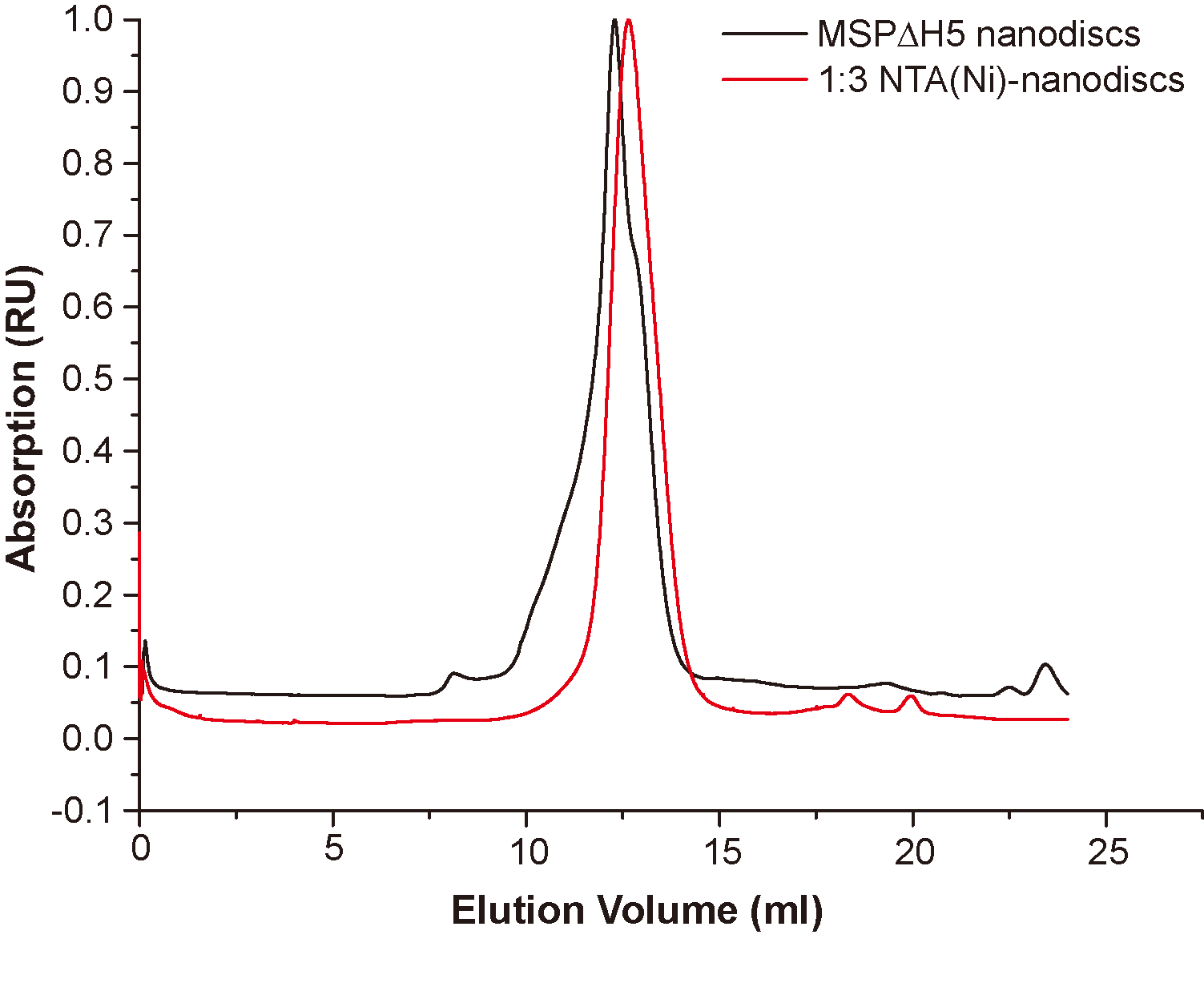

Supplement: Supplemental Information 1 [file peerj-07-7234-s001.png]

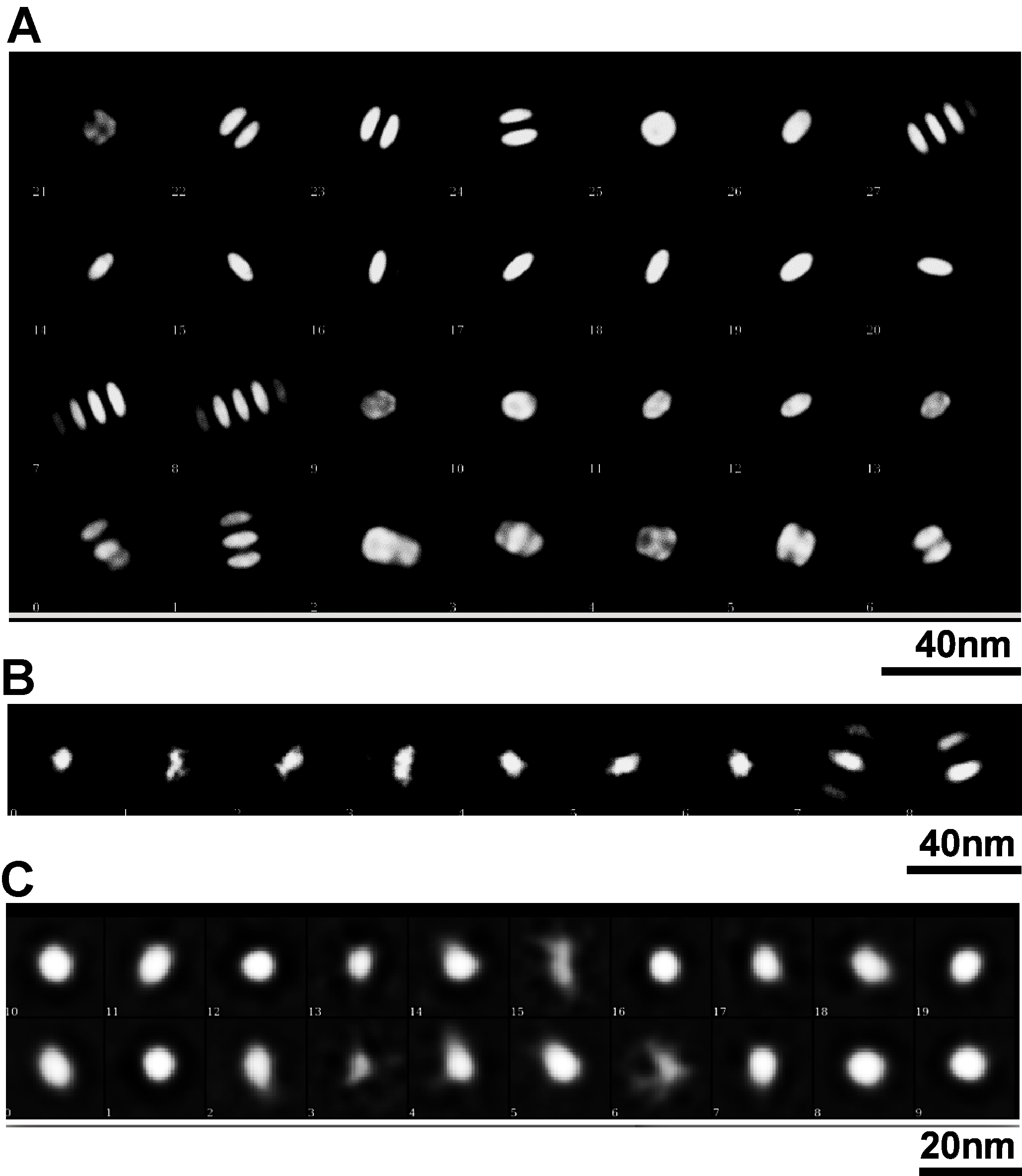

Supplement: Supplemental Information 2 [file peerj-07-7234-s002.png]

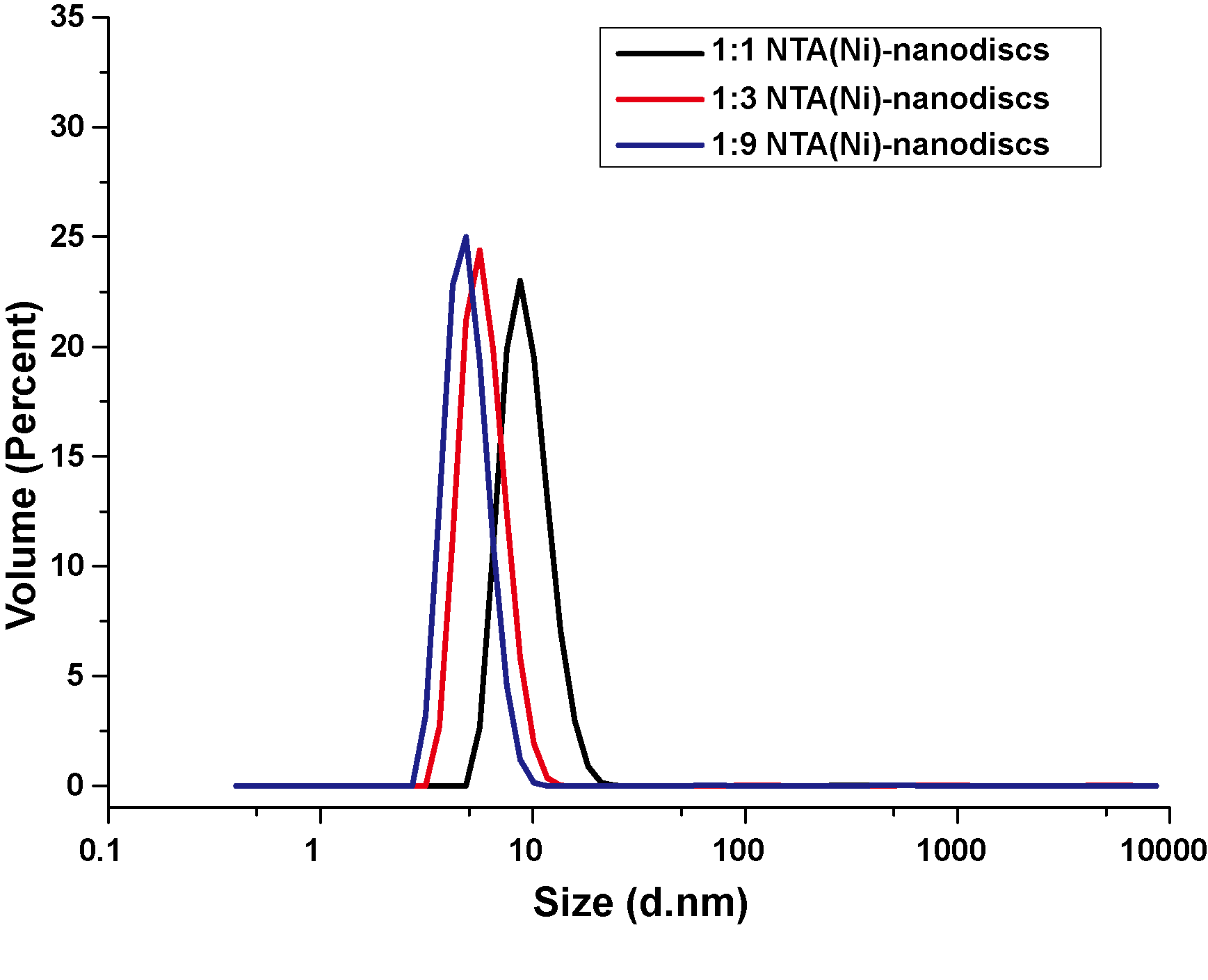

Supplement: Supplemental Information 3 [file peerj-07-7234-s003.png]

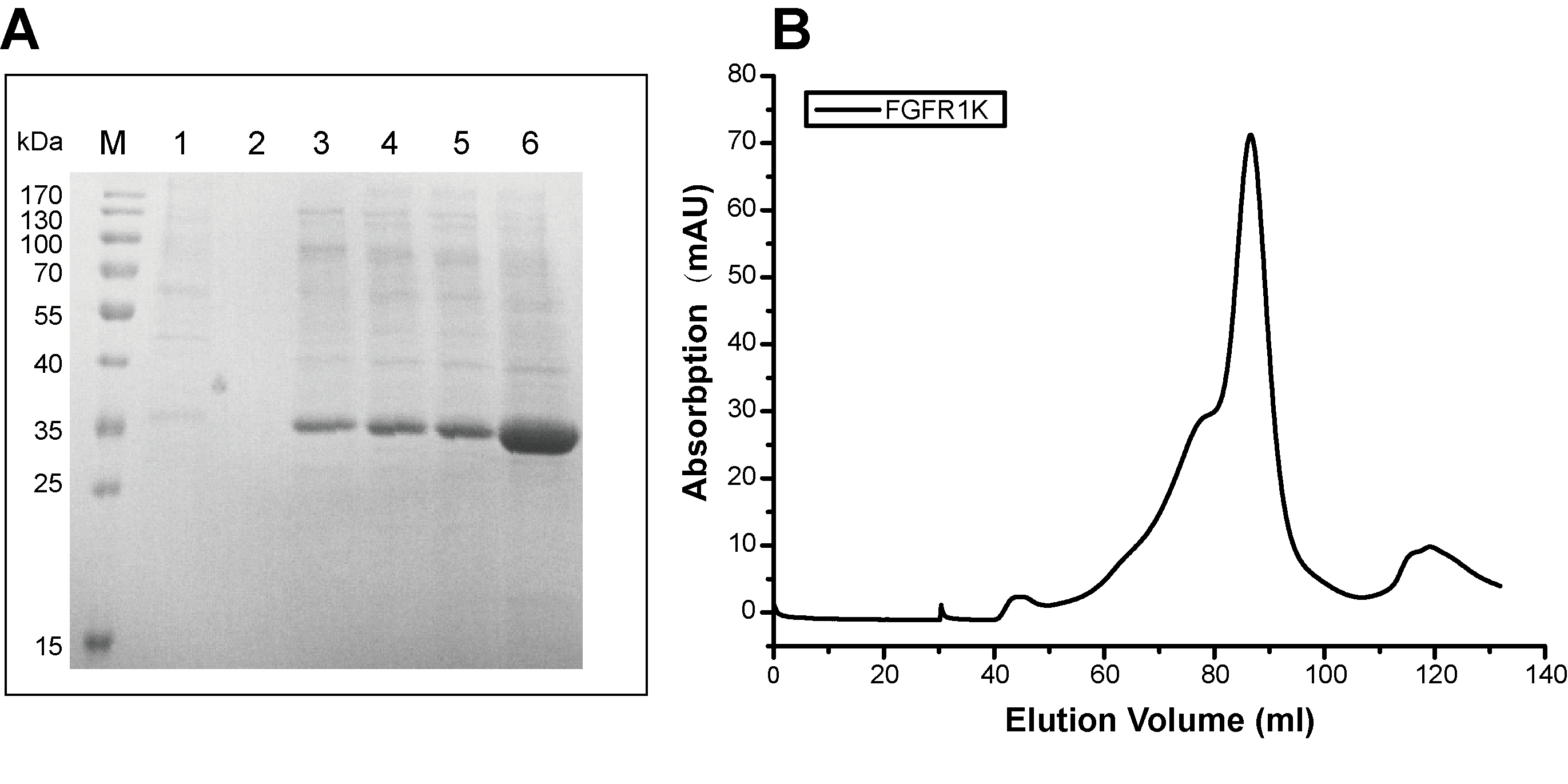

Supplement: Supplemental Information 4 — (A) SDS PAGE gel of elution aliquots from Ni-NTA affinity purification of FGFR1K protein. M: protein marks with molecular weights as labeled; 1: flow through; 2: wash buffer elution; 3, 4, 5: elutions with 30 mM imidazole; 6: elution with 300 mM imidazole. (B) Size exclusion chromatography of FGFR1K (Ni-NTA elution with 300 mM imidazole) with a Superdex 200 GL 16/60 column (GE Healthcare). [file peerj-07-7234-s004.png]

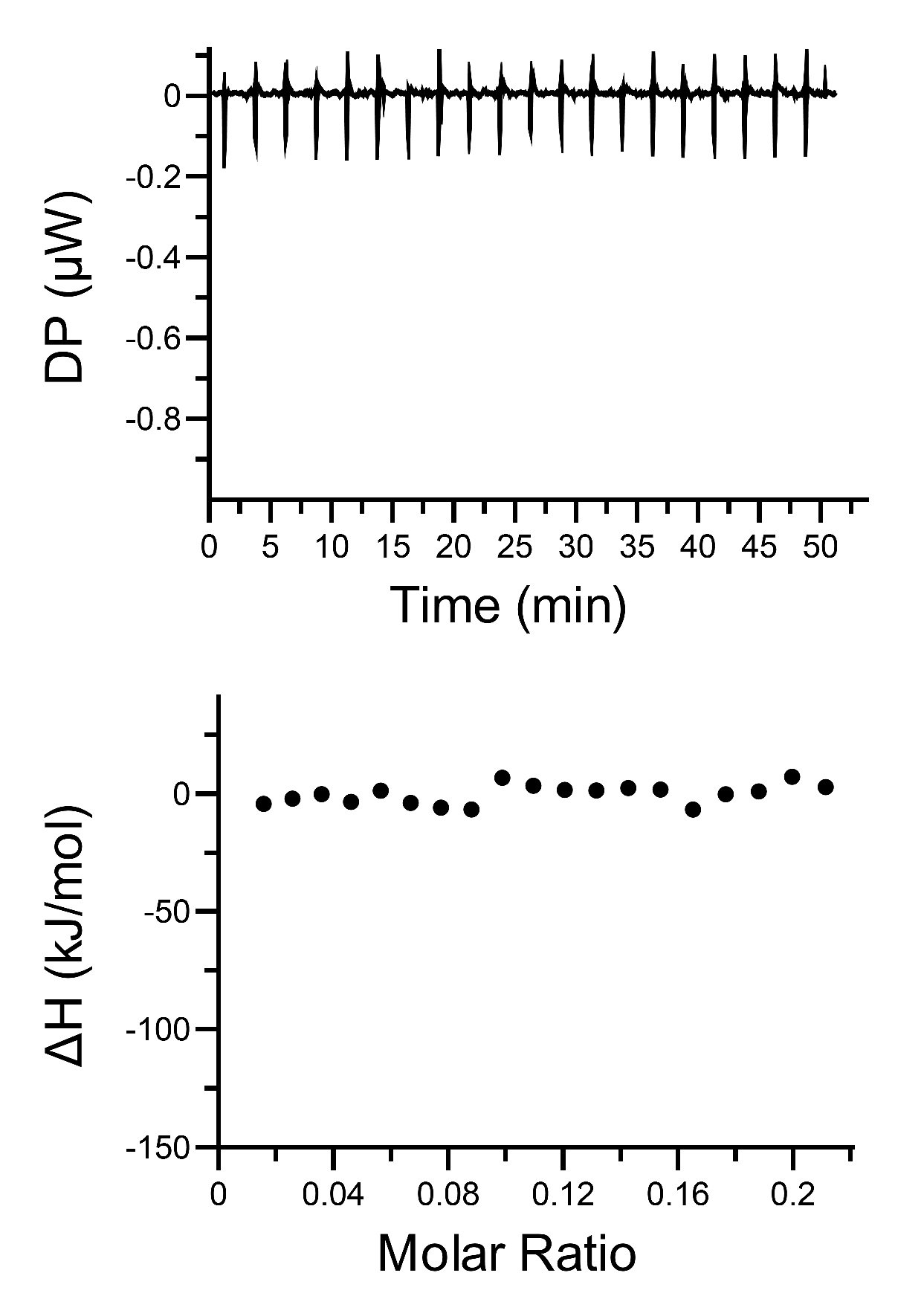

Supplement: Supplemental Information 5 [file peerj-07-7234-s005.png]

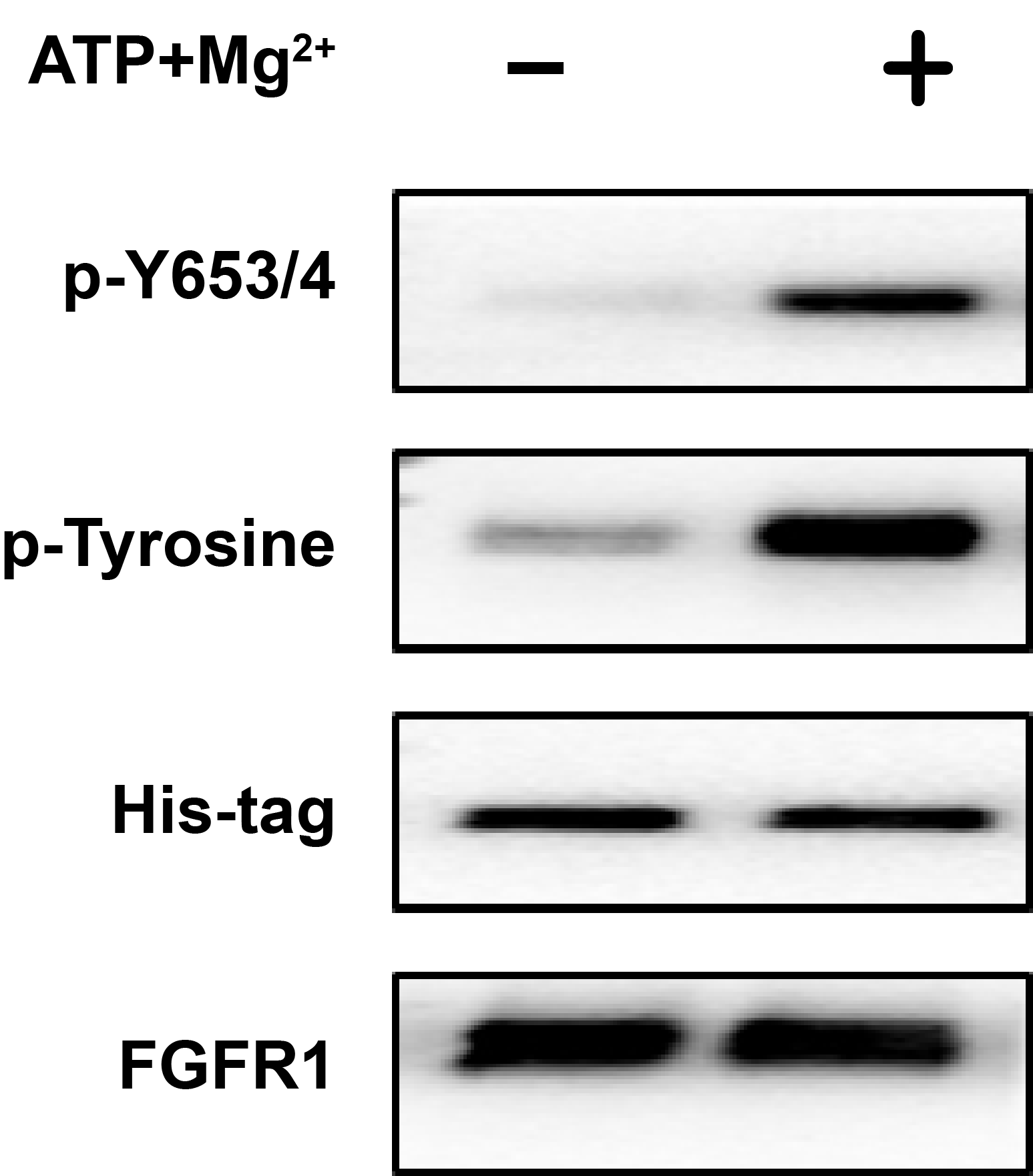

Supplement: Supplemental Information 6 — The total phosphorylation (p-Tyrosine) levels and phosphorylation of Tyrosine 653/654 before (first lane) and after phosphorylation reaction for 10 min (second lane) were characterized by western blotting using specific antibodies. The amount of FGFR1K loaded were verified by western blotting using both His-tag antibody and FGFR1 antibody. [file peerj-07-7234-s006.png]

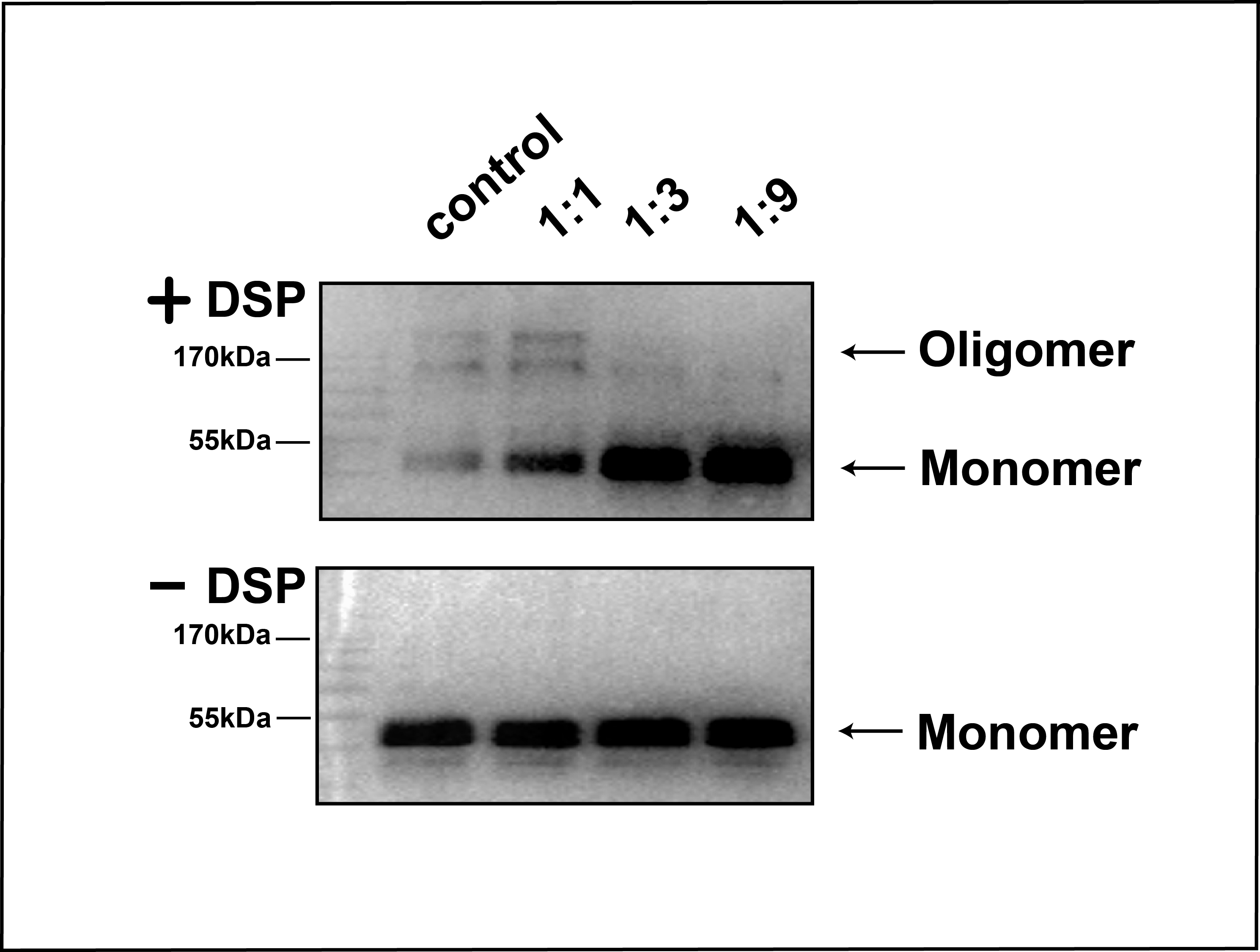

Supplement: Supplemental Information 7 [file peerj-07-7234-s007.png]

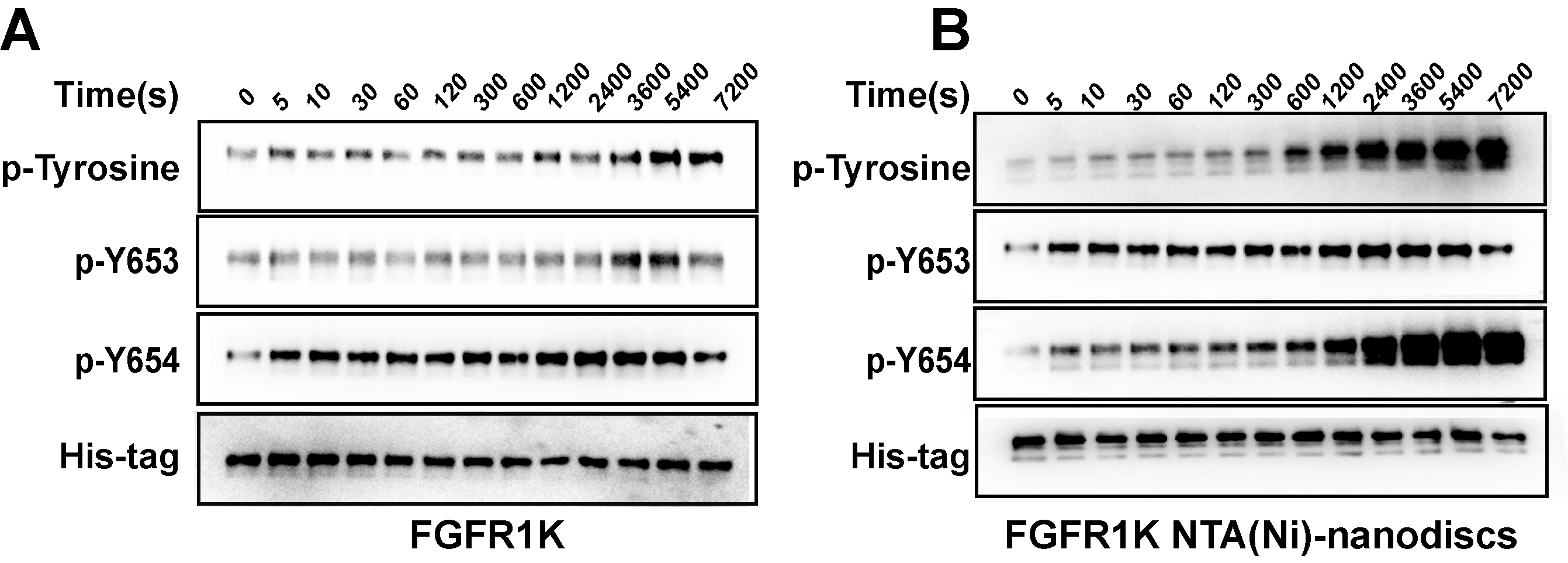

Supplement: Supplemental Information 8 — (A) and on peptide NTA(Ni)-nanodiscs (B). Specific antibodies of phospho-tyrosine, FGFR1 p-Y653, and FGFR1 p-Y654 were used. [file peerj-07-7234-s008.png]

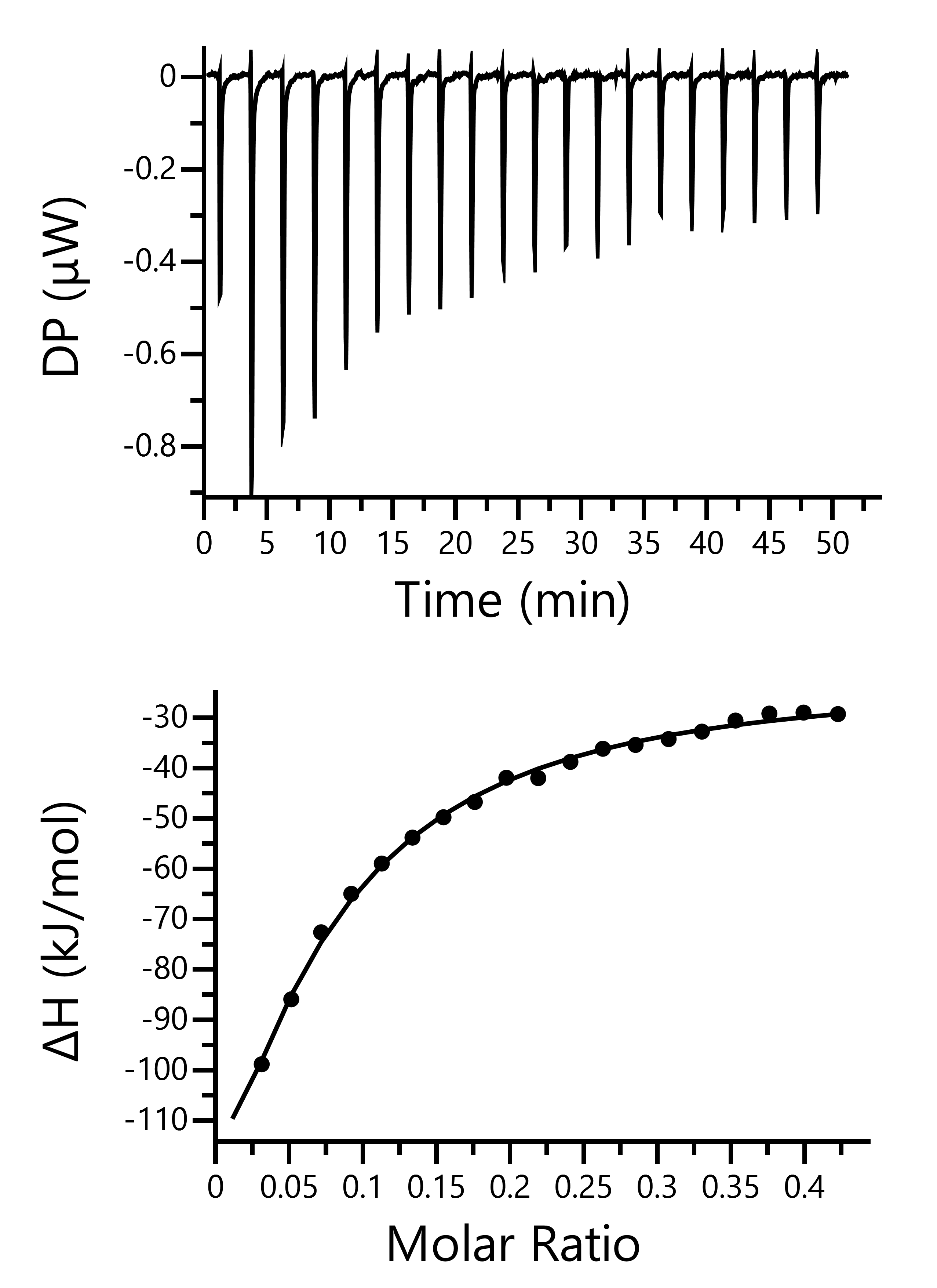

Supplement: Supplemental Information 13 — Raw data exported from ITC for data analyses for Fig. 3B and Fig. S5. [file peerj-07-7234-s013.zip › ITC raw data figure 3B S5/figure 3B raw data.TIF]

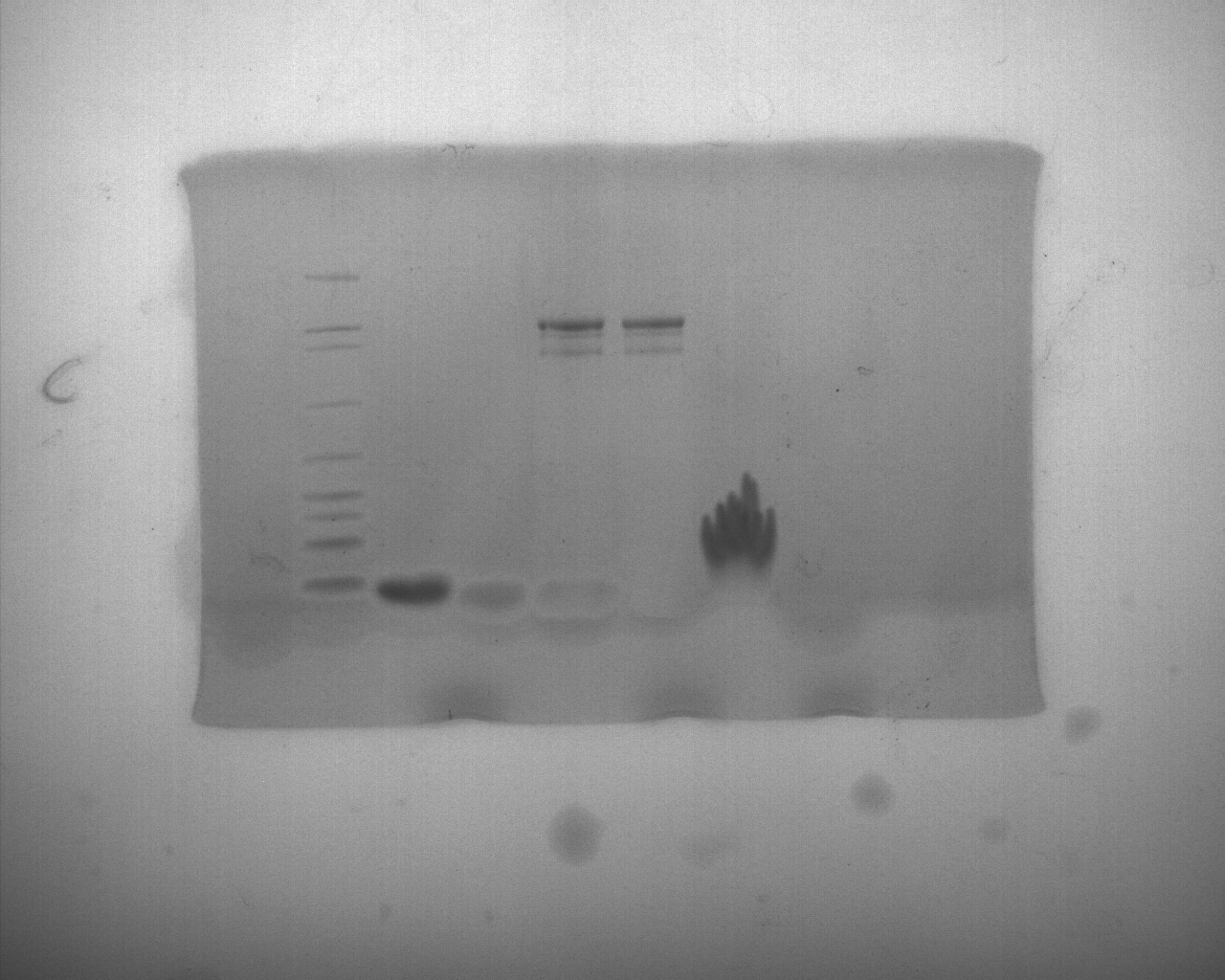

Supplement: Supplemental Information 14 — Raw data exported from SDS PAGE for data analyses for Fig. 3A and Fig. S4A. [file peerj-07-7234-s014.zip › SDS PAGE raw data figure 3A figure S4A/figure 3A raw data.bmp]

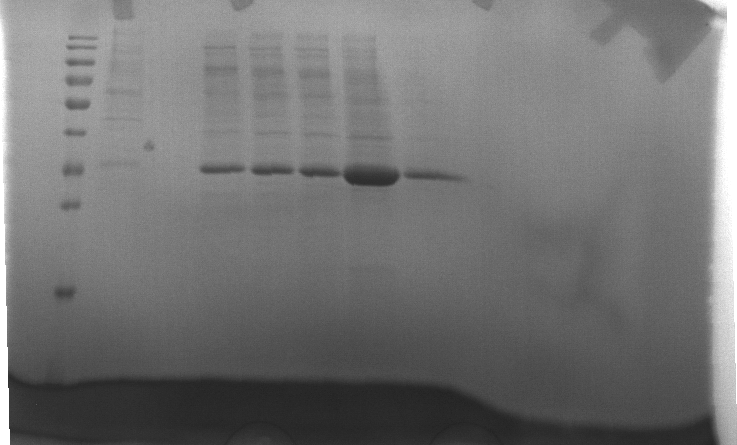

Supplement: Supplemental Information 14 — Raw data exported from SDS PAGE for data analyses for Fig. 3A and Fig. S4A. [file peerj-07-7234-s014.zip › SDS PAGE raw data figure 3A figure S4A/figure S4 A raw data.png]

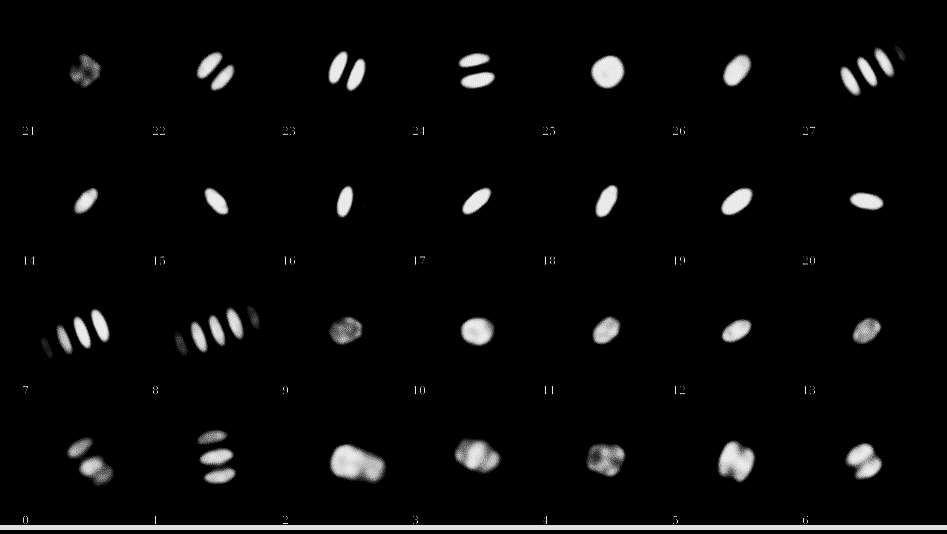

Supplement: Supplemental Information 15 — Raw data exported from TEM for data analyses for Figs. 2B–2D and Fig. S2. [file peerj-07-7234-s015.zip › TEM raw data Figure 2B-D S2/figue S2 raw data/1-1.png]

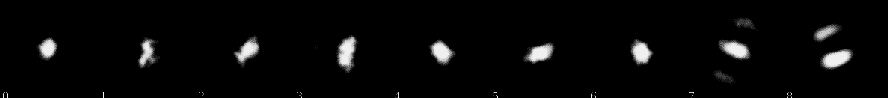

Supplement: Supplemental Information 15 — Raw data exported from TEM for data analyses for Figs. 2B–2D and Fig. S2. [file peerj-07-7234-s015.zip › TEM raw data Figure 2B-D S2/figue S2 raw data/1-3.png]

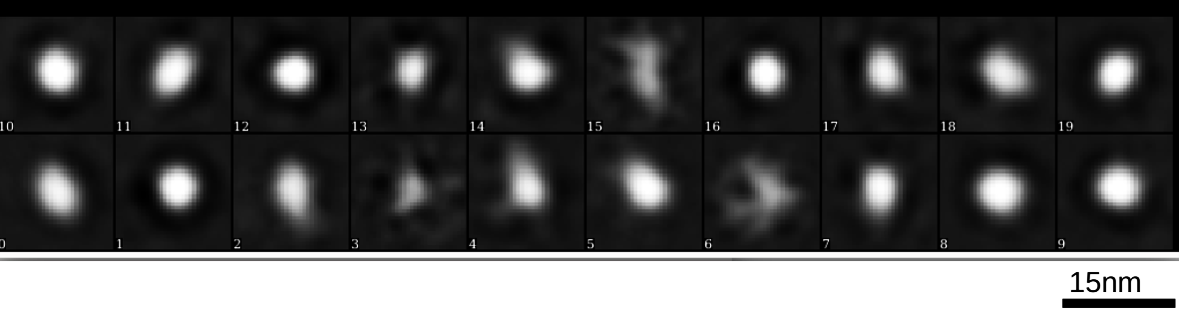

Supplement: Supplemental Information 15 — Raw data exported from TEM for data analyses for Figs. 2B–2D and Fig. S2. [file peerj-07-7234-s015.zip › TEM raw data Figure 2B-D S2/figue S2 raw data/1-9.png]

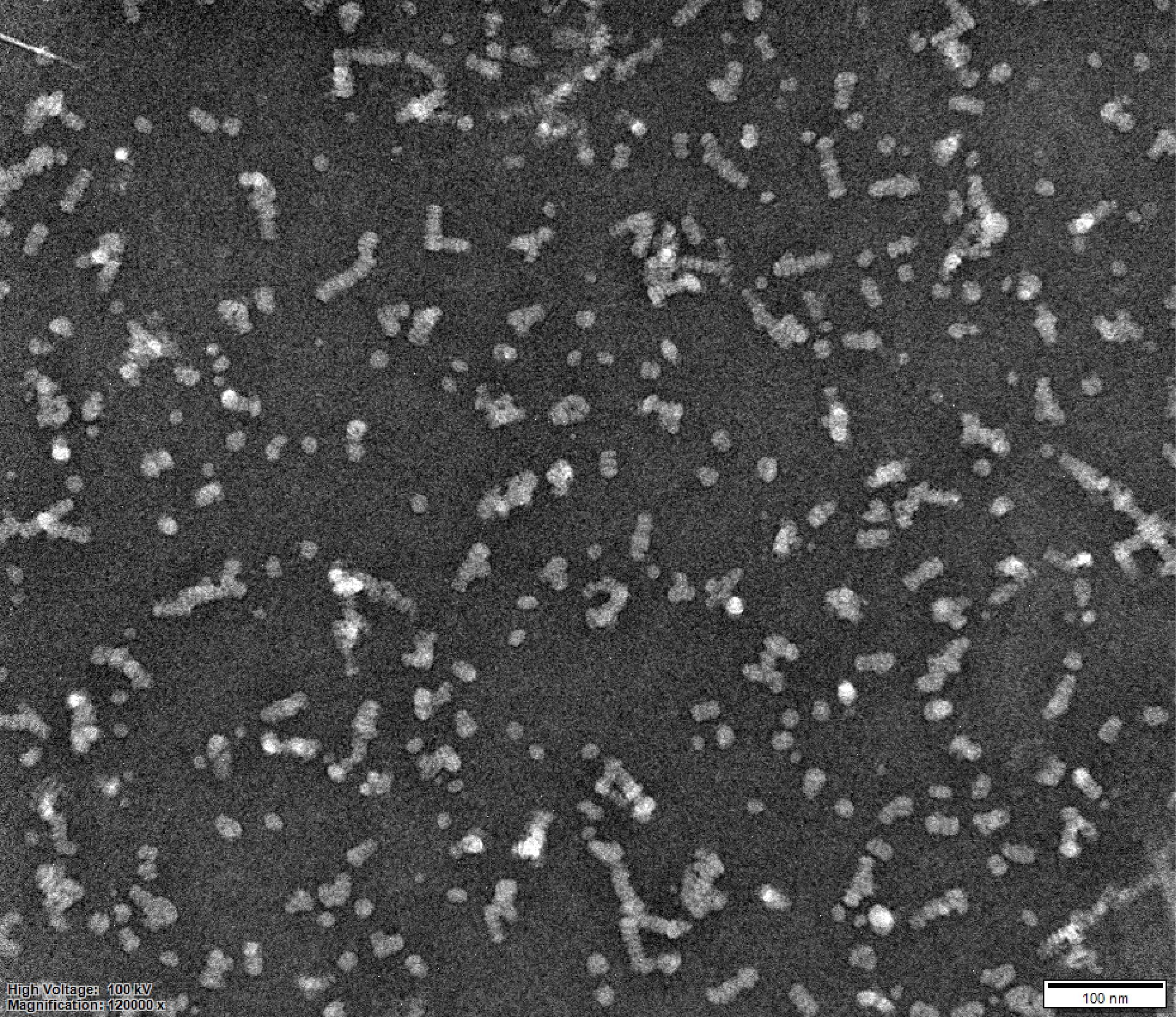

Supplement: Supplemental Information 15 — Raw data exported from TEM for data analyses for Figs. 2B–2D and Fig. S2. [file peerj-07-7234-s015.zip › TEM raw data Figure 2B-D S2/figure 2B-D raw data/TEM 1-1 raw data.jpg]

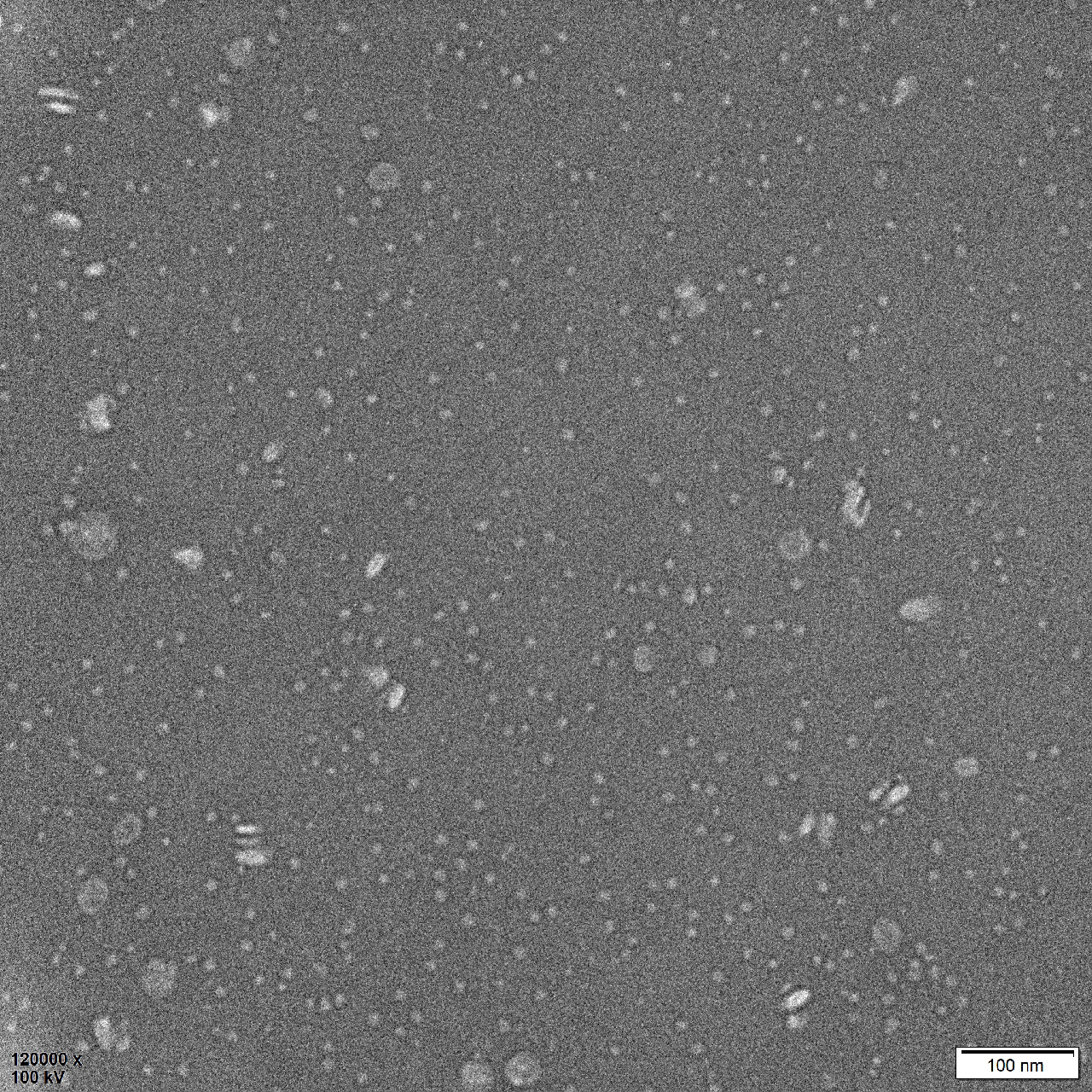

Supplement: Supplemental Information 15 — Raw data exported from TEM for data analyses for Figs. 2B–2D and Fig. S2. [file peerj-07-7234-s015.zip › TEM raw data Figure 2B-D S2/figure 2B-D raw data/TEM 1-3 raw data.jpg]

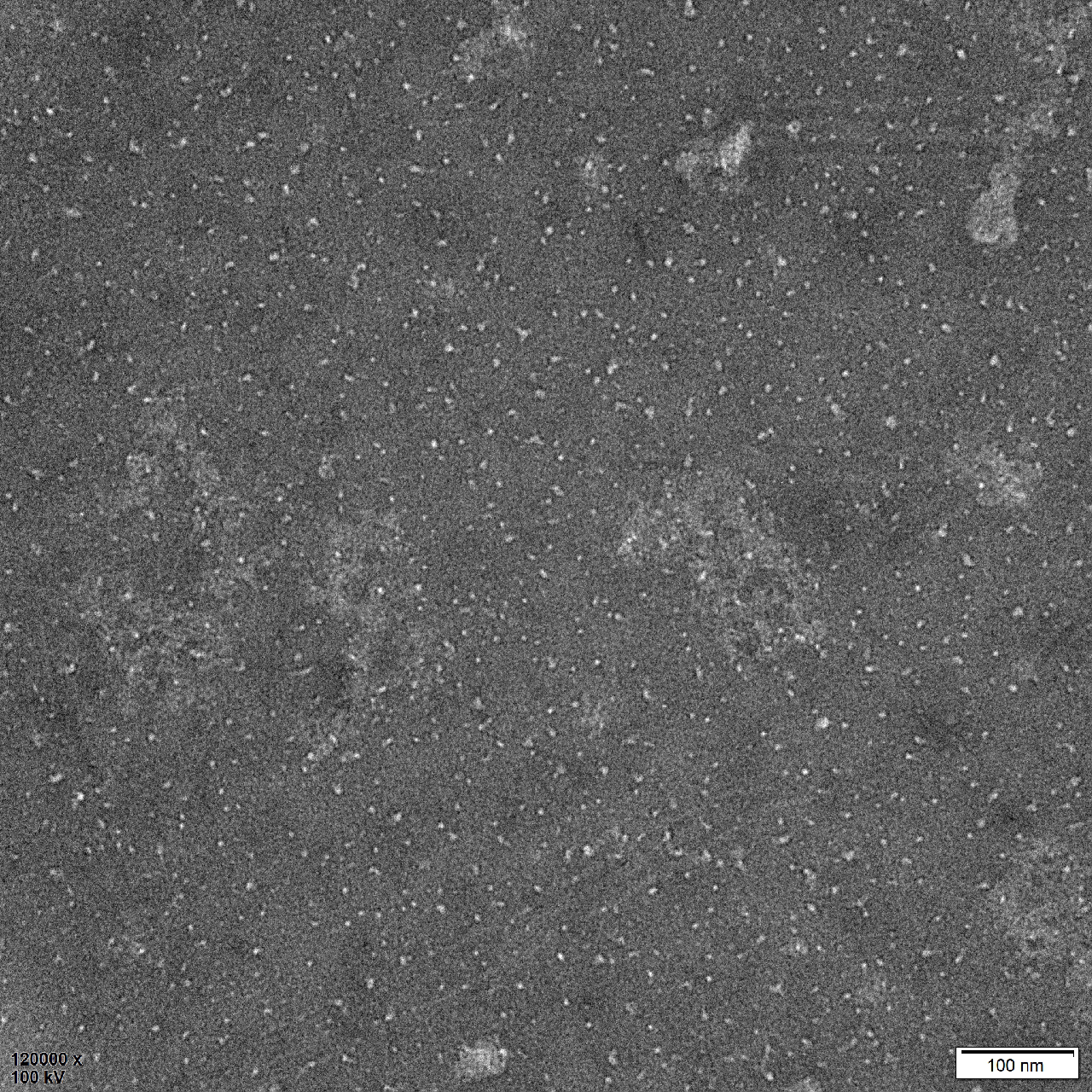

Supplement: Supplemental Information 15 — Raw data exported from TEM for data analyses for Figs. 2B–2D and Fig. S2. [file peerj-07-7234-s015.zip › TEM raw data Figure 2B-D S2/figure 2B-D raw data/TEM 1-9 raw data.jpg]

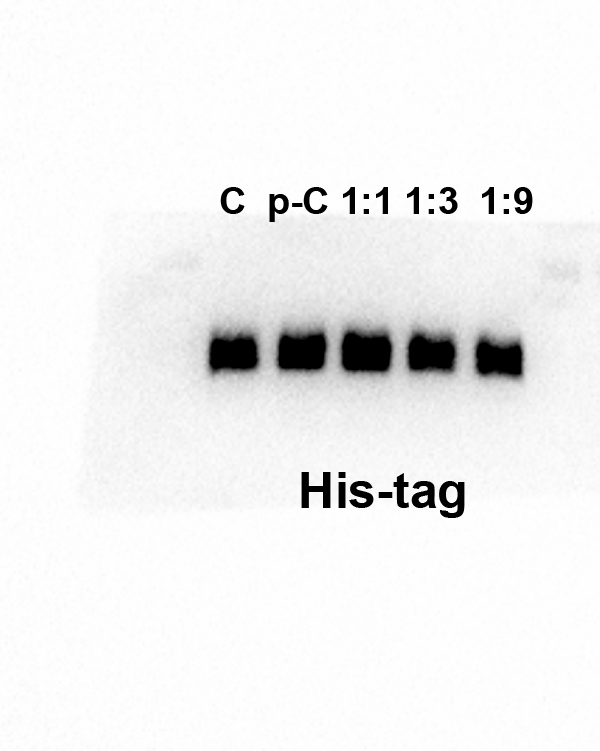

Supplement: Supplemental Information 16 — Raw data exported from western-blot for data analyses for Figs. 3C, 4 and Figs. S6–S8. [file peerj-07-7234-s016.zip › Western blot raw data figure 3C 4 s6 s7 s8/Figure 3C raw data/HIS.png]

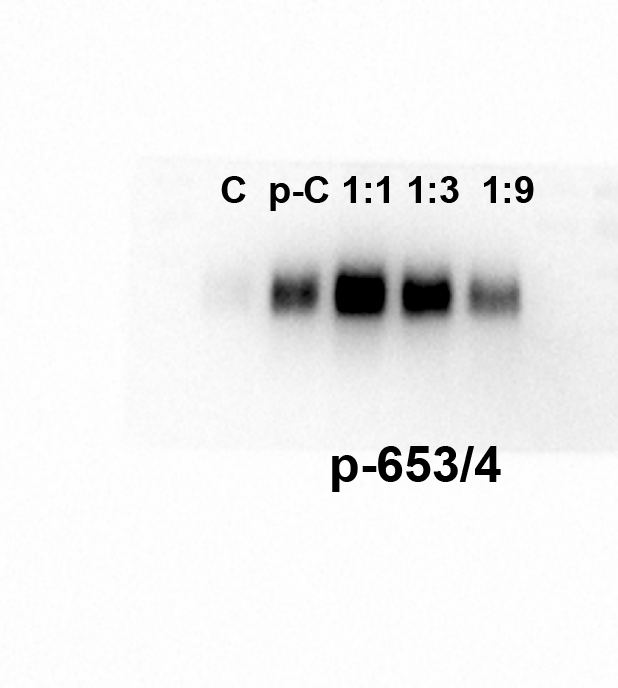

Supplement: Supplemental Information 16 — Raw data exported from western-blot for data analyses for Figs. 3C, 4 and Figs. S6–S8. [file peerj-07-7234-s016.zip › Western blot raw data figure 3C 4 s6 s7 s8/Figure 3C raw data/p-6534.png]

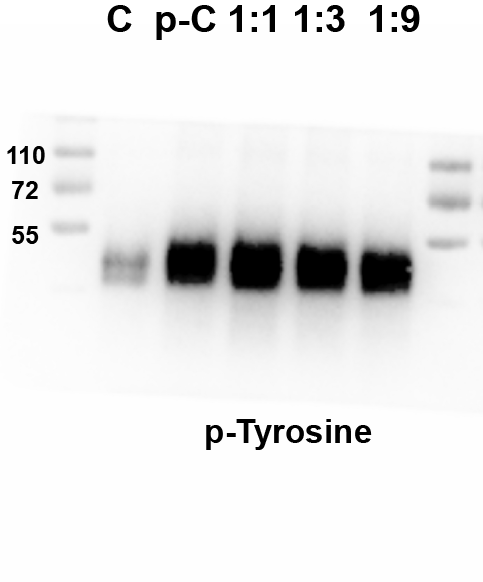

Supplement: Supplemental Information 16 — Raw data exported from western-blot for data analyses for Figs. 3C, 4 and Figs. S6–S8. [file peerj-07-7234-s016.zip › Western blot raw data figure 3C 4 s6 s7 s8/Figure 3C raw data/p-Tyrosine.png]

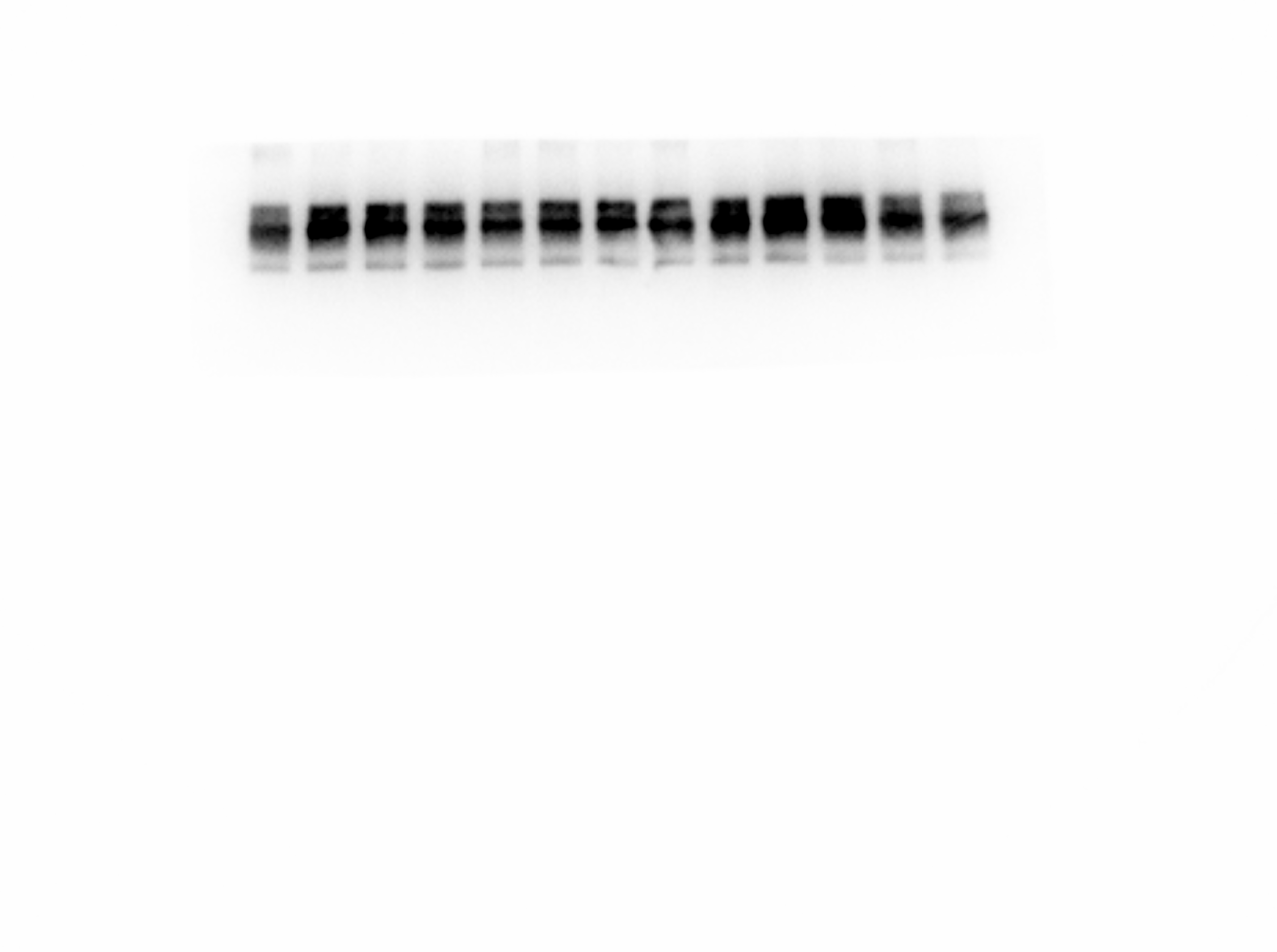

Supplement: Supplemental Information 16 — Raw data exported from western-blot for data analyses for Figs. 3C, 4 and Figs. S6–S8. [file peerj-07-7234-s016.zip › Western blot raw data figure 3C 4 s6 s7 s8/Figure 4 raw data/C1/C P653.jpg]

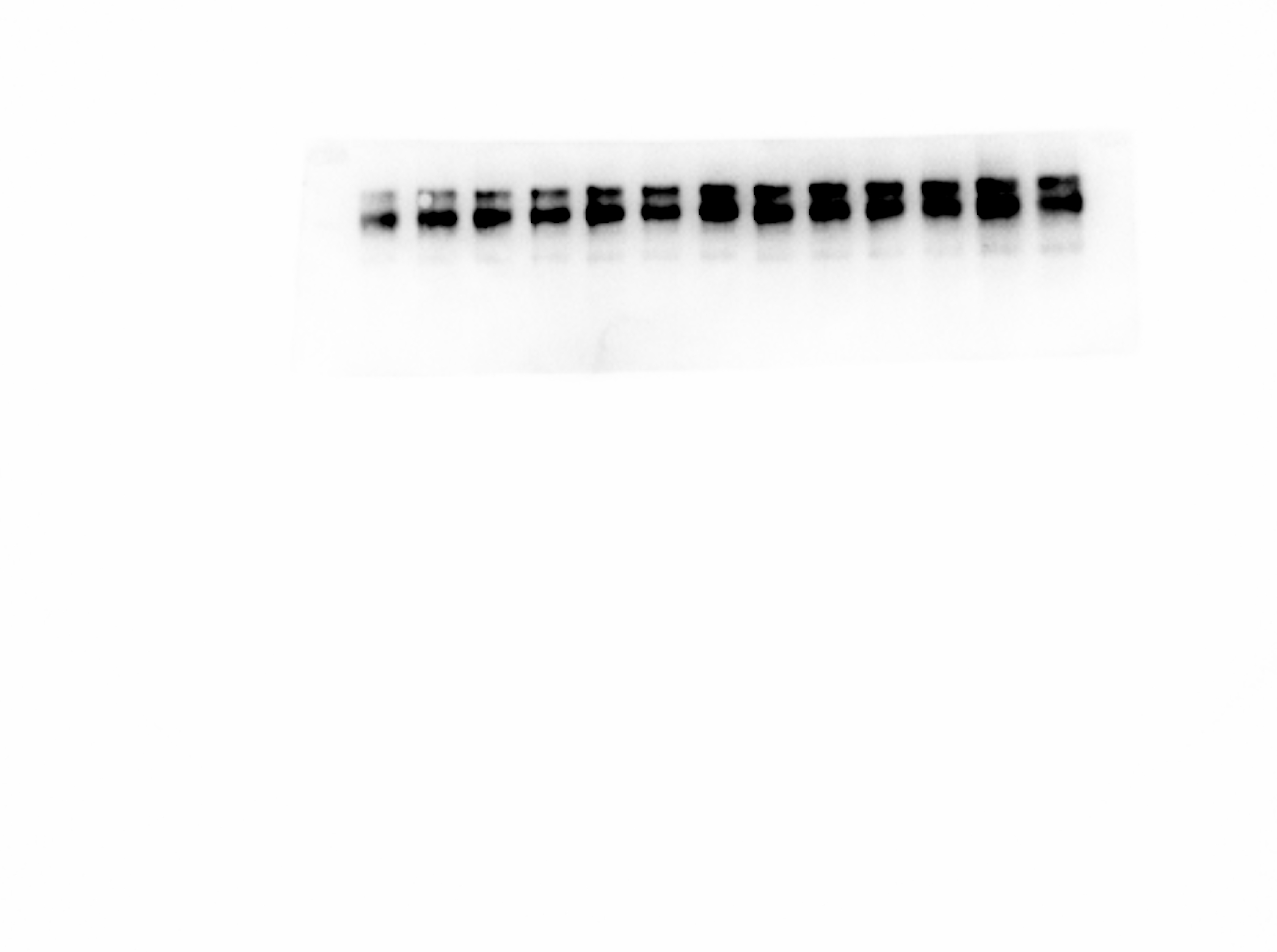

Supplement: Supplemental Information 16 — Raw data exported from western-blot for data analyses for Figs. 3C, 4 and Figs. S6–S8. [file peerj-07-7234-s016.zip › Western blot raw data figure 3C 4 s6 s7 s8/Figure 4 raw data/C1/C P654.jpg]

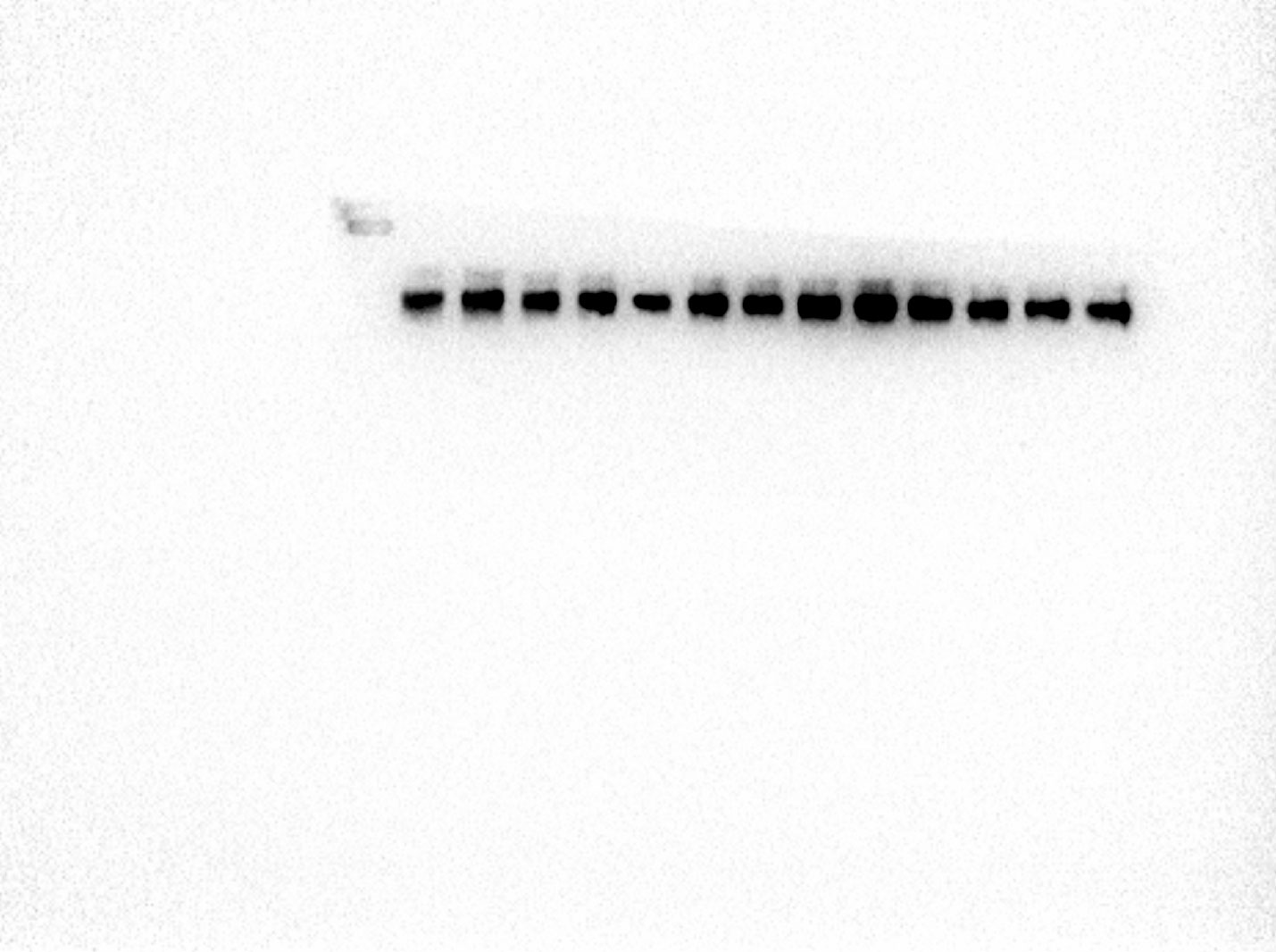

Supplement: Supplemental Information 16 — Raw data exported from western-blot for data analyses for Figs. 3C, 4 and Figs. S6–S8. [file peerj-07-7234-s016.zip › Western blot raw data figure 3C 4 s6 s7 s8/Figure 4 raw data/C1/C HIS.jpg]

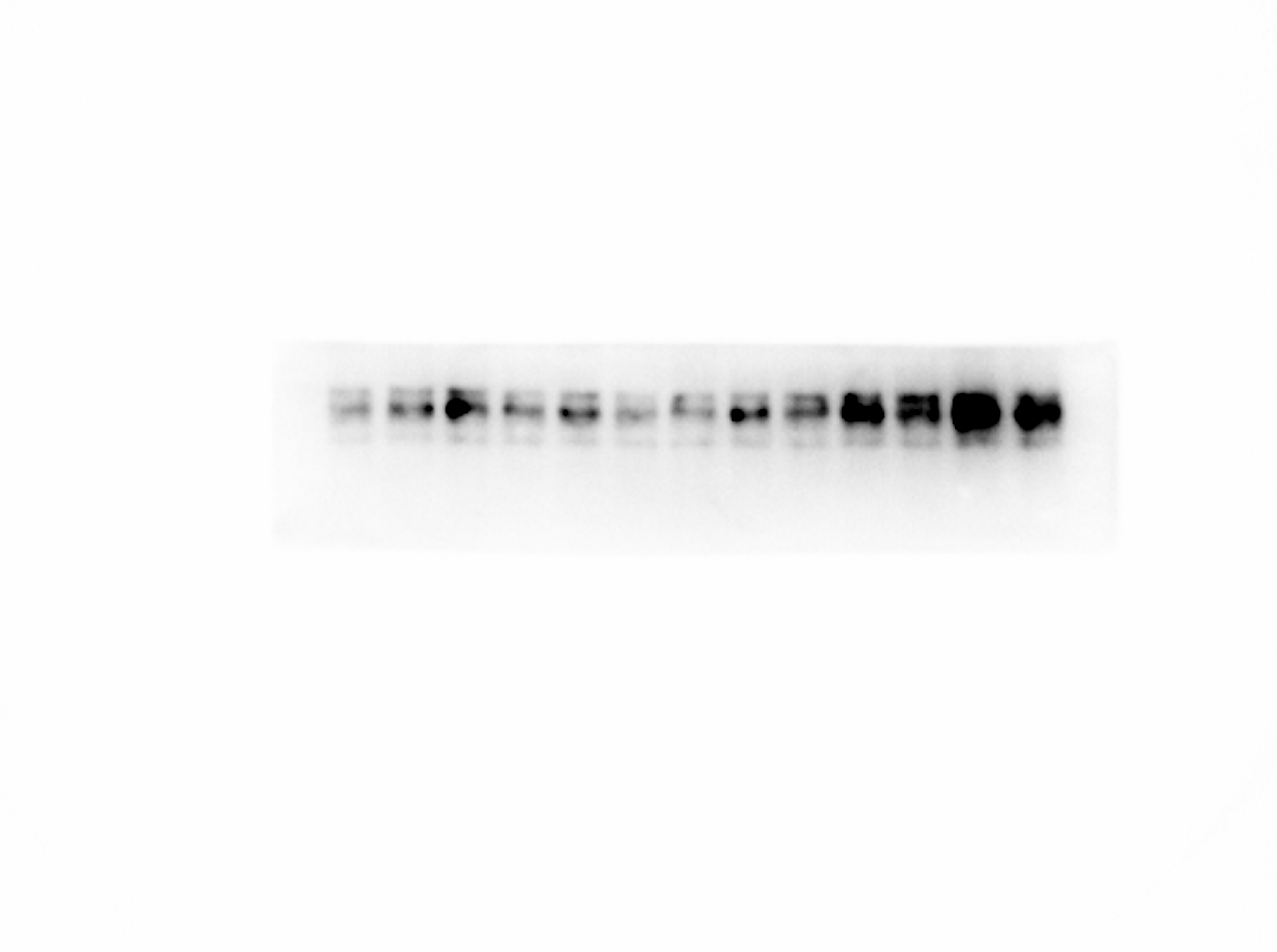

Supplement: Supplemental Information 16 — Raw data exported from western-blot for data analyses for Figs. 3C, 4 and Figs. S6–S8. [file peerj-07-7234-s016.zip › Western blot raw data figure 3C 4 s6 s7 s8/Figure 4 raw data/C1/C PY.jpg]

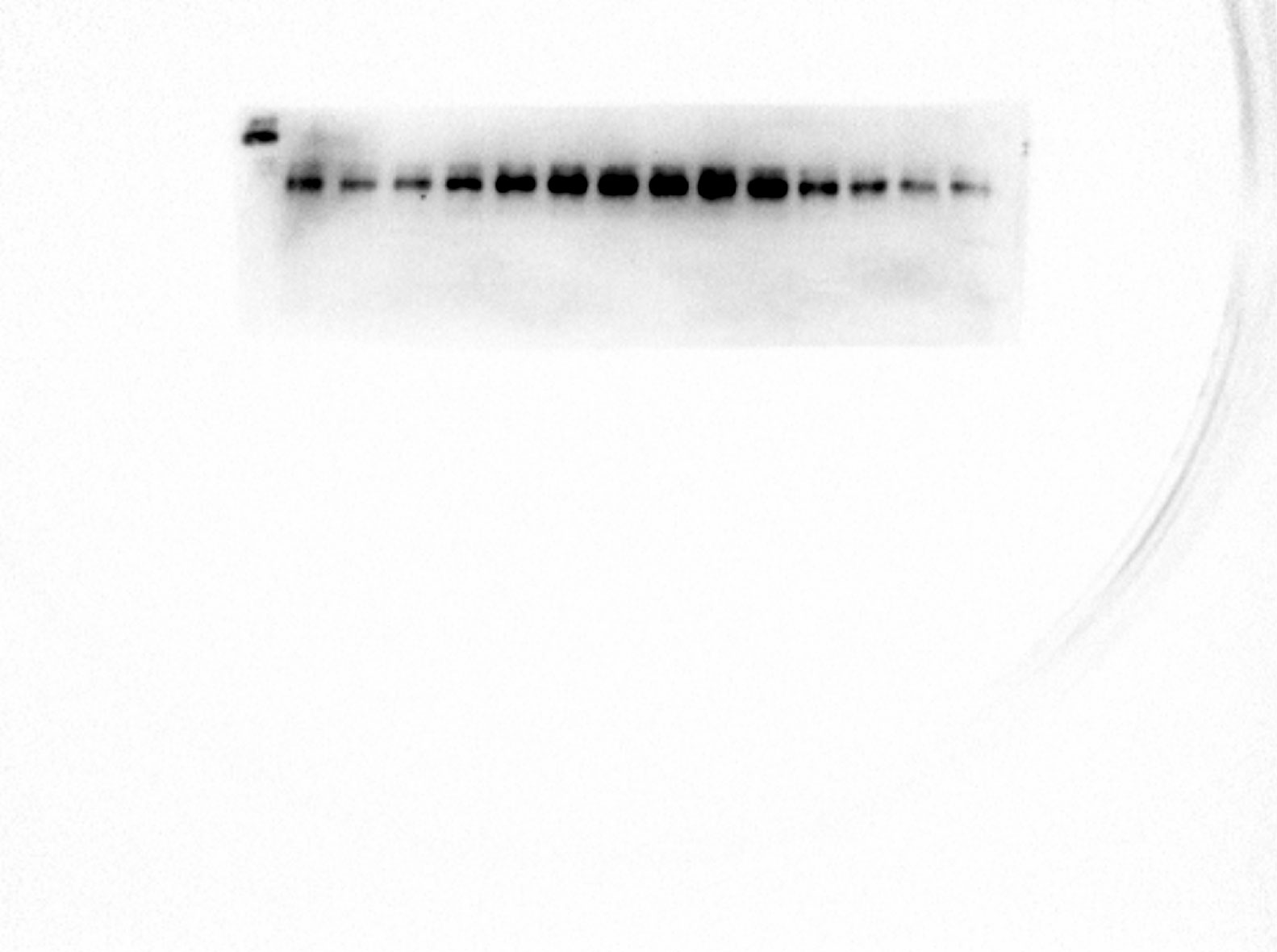

Supplement: Supplemental Information 16 — Raw data exported from western-blot for data analyses for Figs. 3C, 4 and Figs. S6–S8. [file peerj-07-7234-s016.zip › Western blot raw data figure 3C 4 s6 s7 s8/Figure 4 raw data/C2/C HIS.jpg]

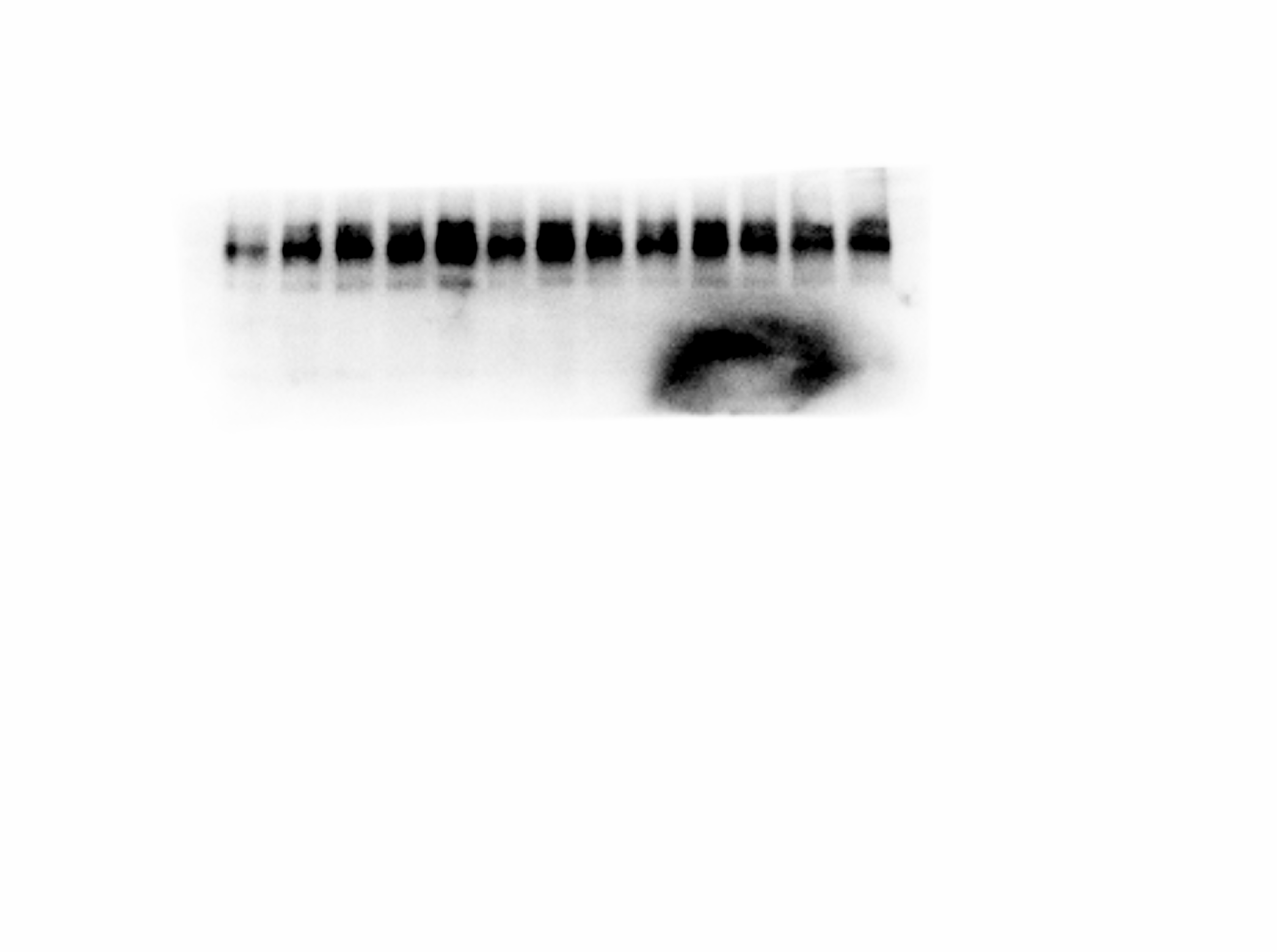

Supplement: Supplemental Information 16 — Raw data exported from western-blot for data analyses for Figs. 3C, 4 and Figs. S6–S8. [file peerj-07-7234-s016.zip › Western blot raw data figure 3C 4 s6 s7 s8/Figure 4 raw data/C2/C P-Y.jpg]

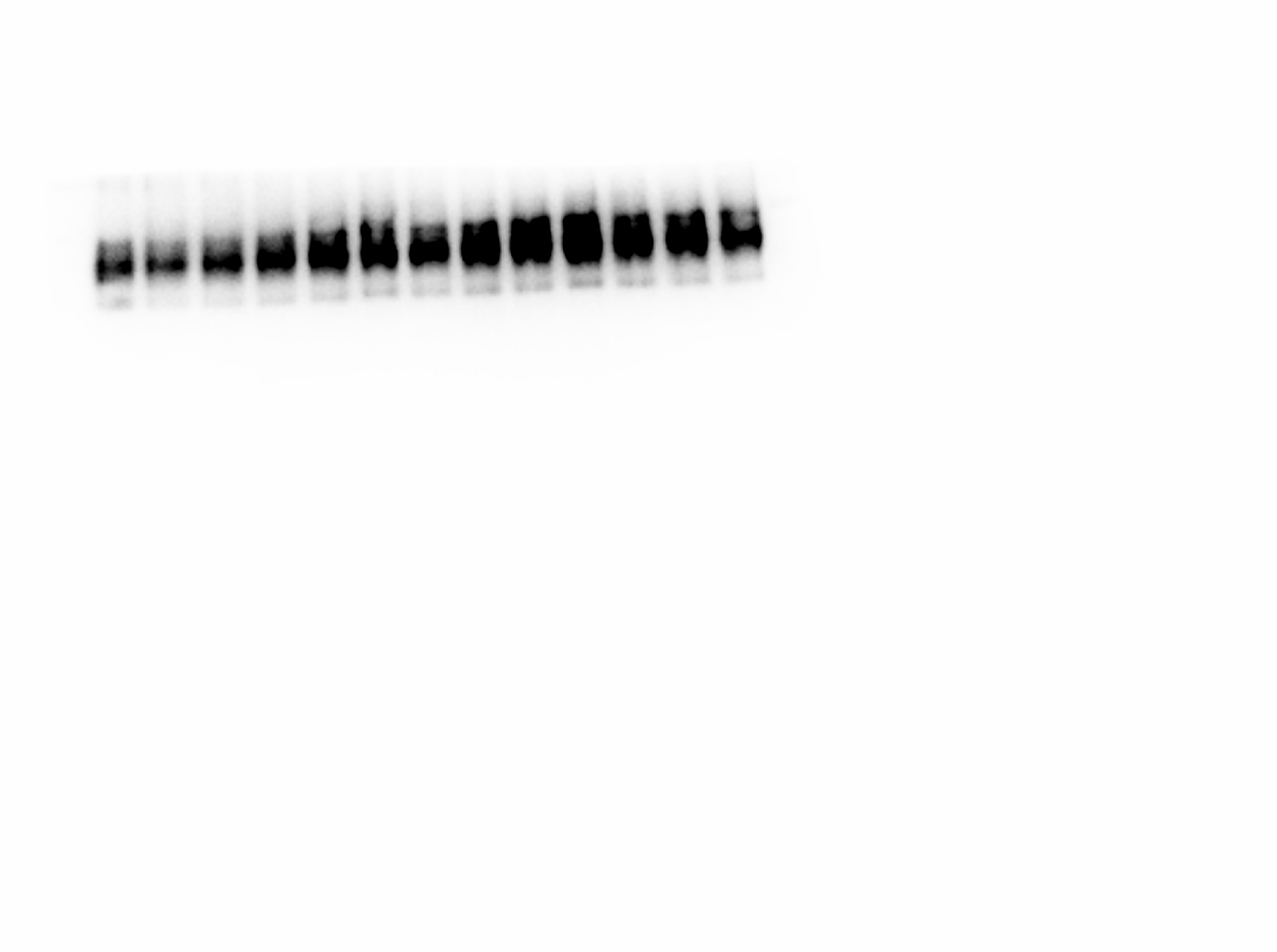

Supplement: Supplemental Information 16 — Raw data exported from western-blot for data analyses for Figs. 3C, 4 and Figs. S6–S8. [file peerj-07-7234-s016.zip › Western blot raw data figure 3C 4 s6 s7 s8/Figure 4 raw data/C2/C P653.jpg]

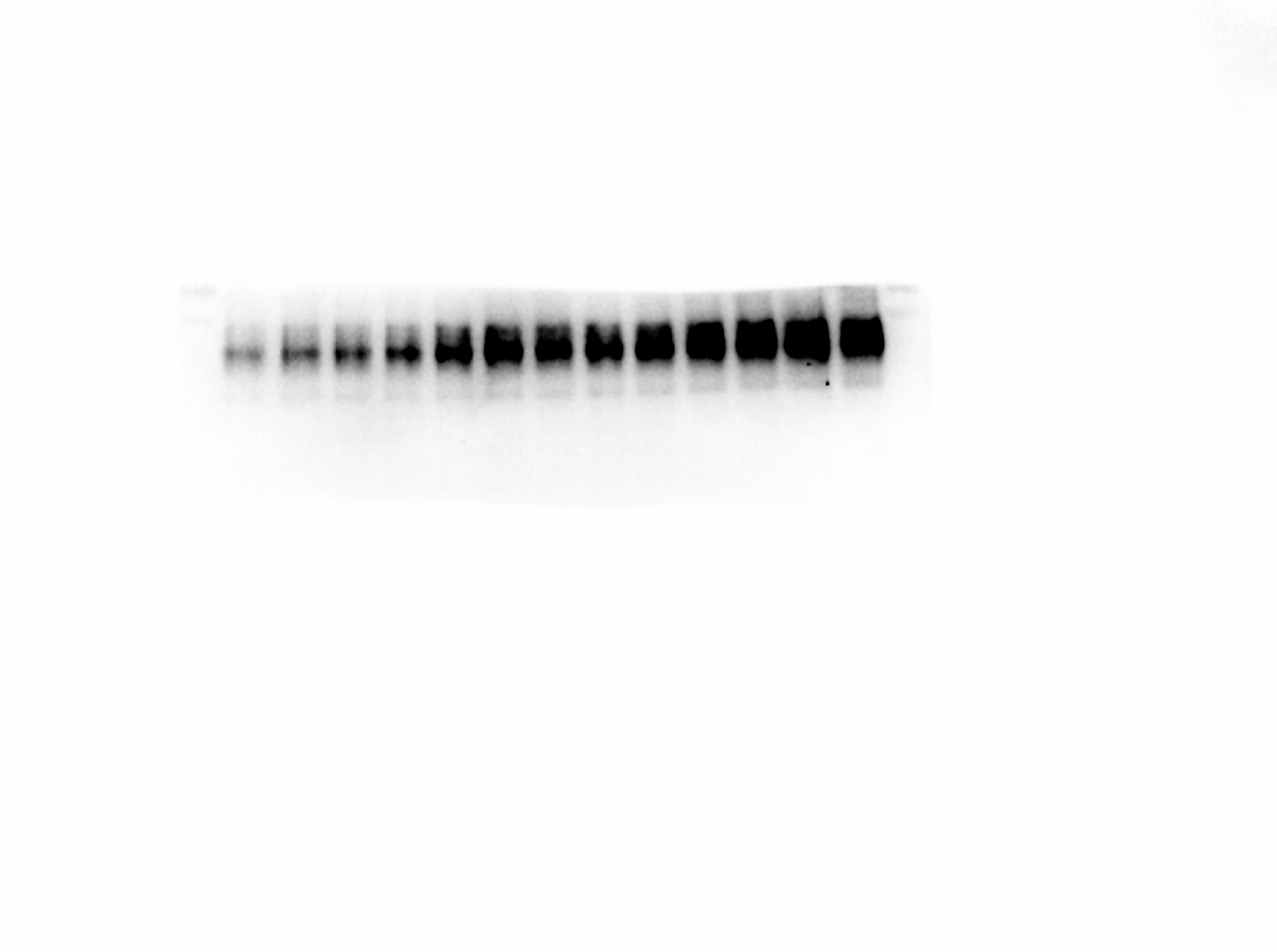

Supplement: Supplemental Information 16 — Raw data exported from western-blot for data analyses for Figs. 3C, 4 and Figs. S6–S8. [file peerj-07-7234-s016.zip › Western blot raw data figure 3C 4 s6 s7 s8/Figure 4 raw data/C2/C P654.jpg]

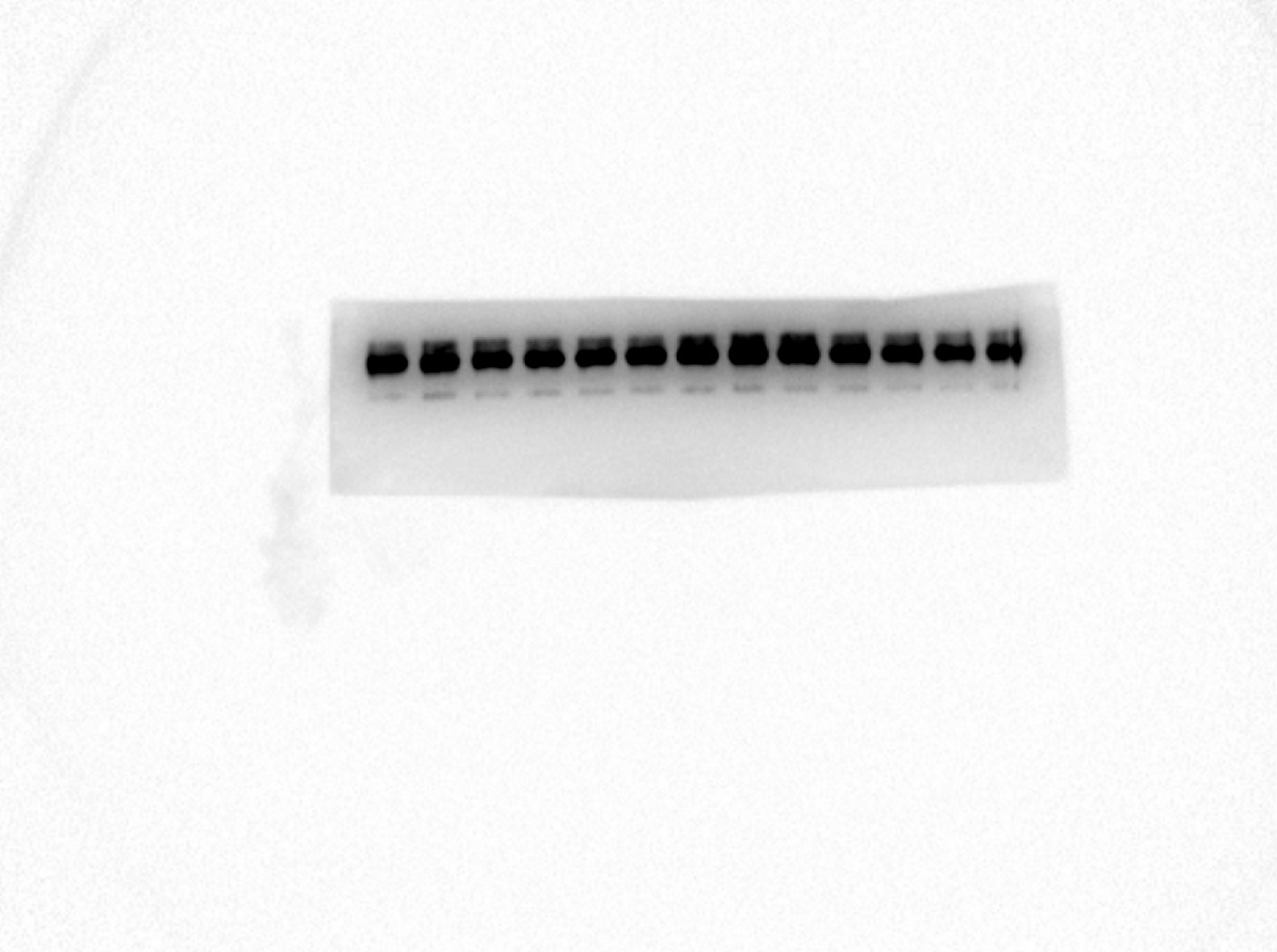

Supplement: Supplemental Information 16 — Raw data exported from western-blot for data analyses for Figs. 3C, 4 and Figs. S6–S8. [file peerj-07-7234-s016.zip › Western blot raw data figure 3C 4 s6 s7 s8/Figure 4 raw data/C3/C His.jpg]

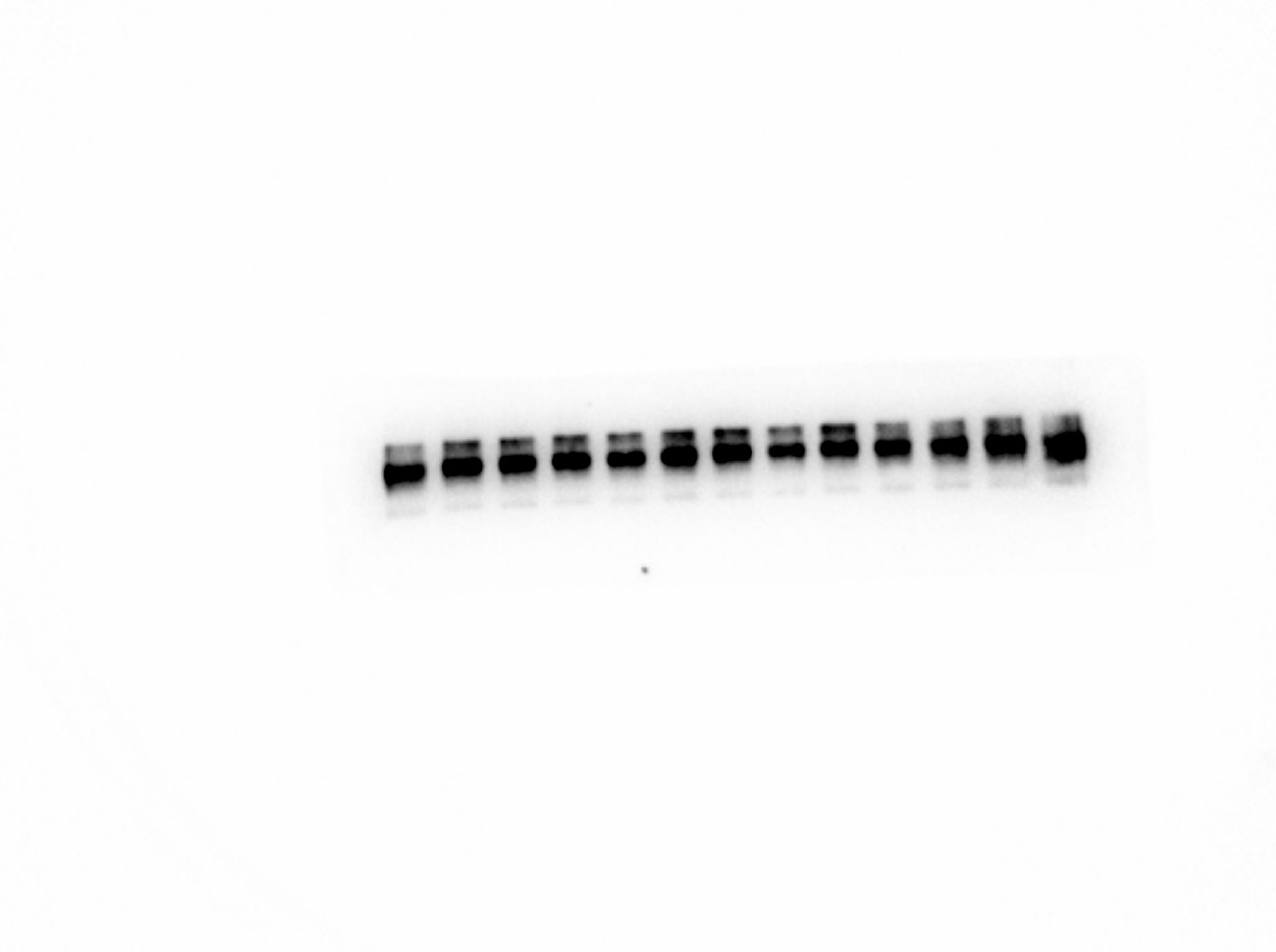

Supplement: Supplemental Information 16 — Raw data exported from western-blot for data analyses for Figs. 3C, 4 and Figs. S6–S8. [file peerj-07-7234-s016.zip › Western blot raw data figure 3C 4 s6 s7 s8/Figure 4 raw data/C3/c p653.jpg]

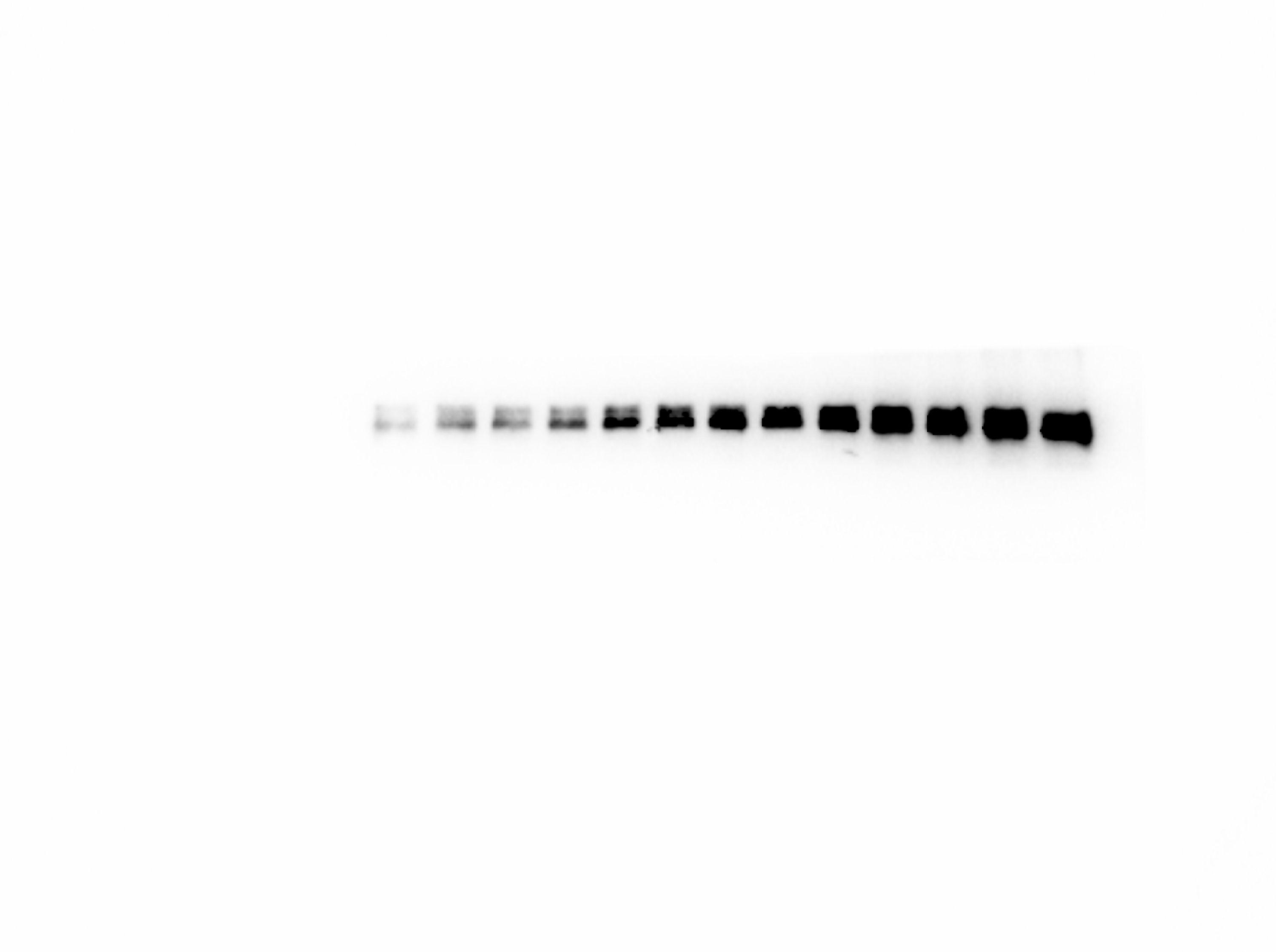

Supplement: Supplemental Information 16 — Raw data exported from western-blot for data analyses for Figs. 3C, 4 and Figs. S6–S8. [file peerj-07-7234-s016.zip › Western blot raw data figure 3C 4 s6 s7 s8/Figure 4 raw data/C3/c p654.jpg]

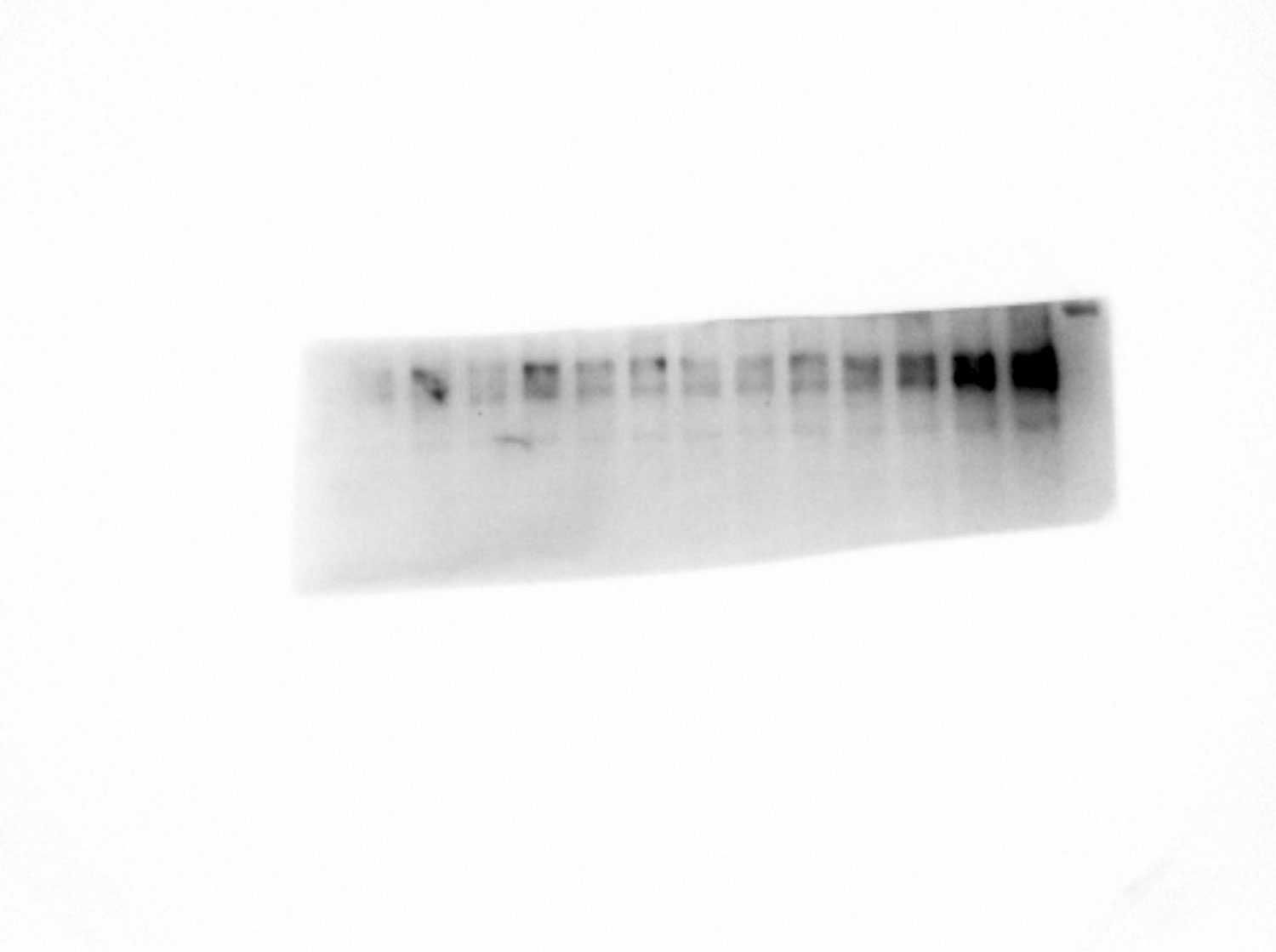

Supplement: Supplemental Information 16 — Raw data exported from western-blot for data analyses for Figs. 3C, 4 and Figs. S6–S8. [file peerj-07-7234-s016.zip › Western blot raw data figure 3C 4 s6 s7 s8/Figure 4 raw data/C3/c py.jpg]

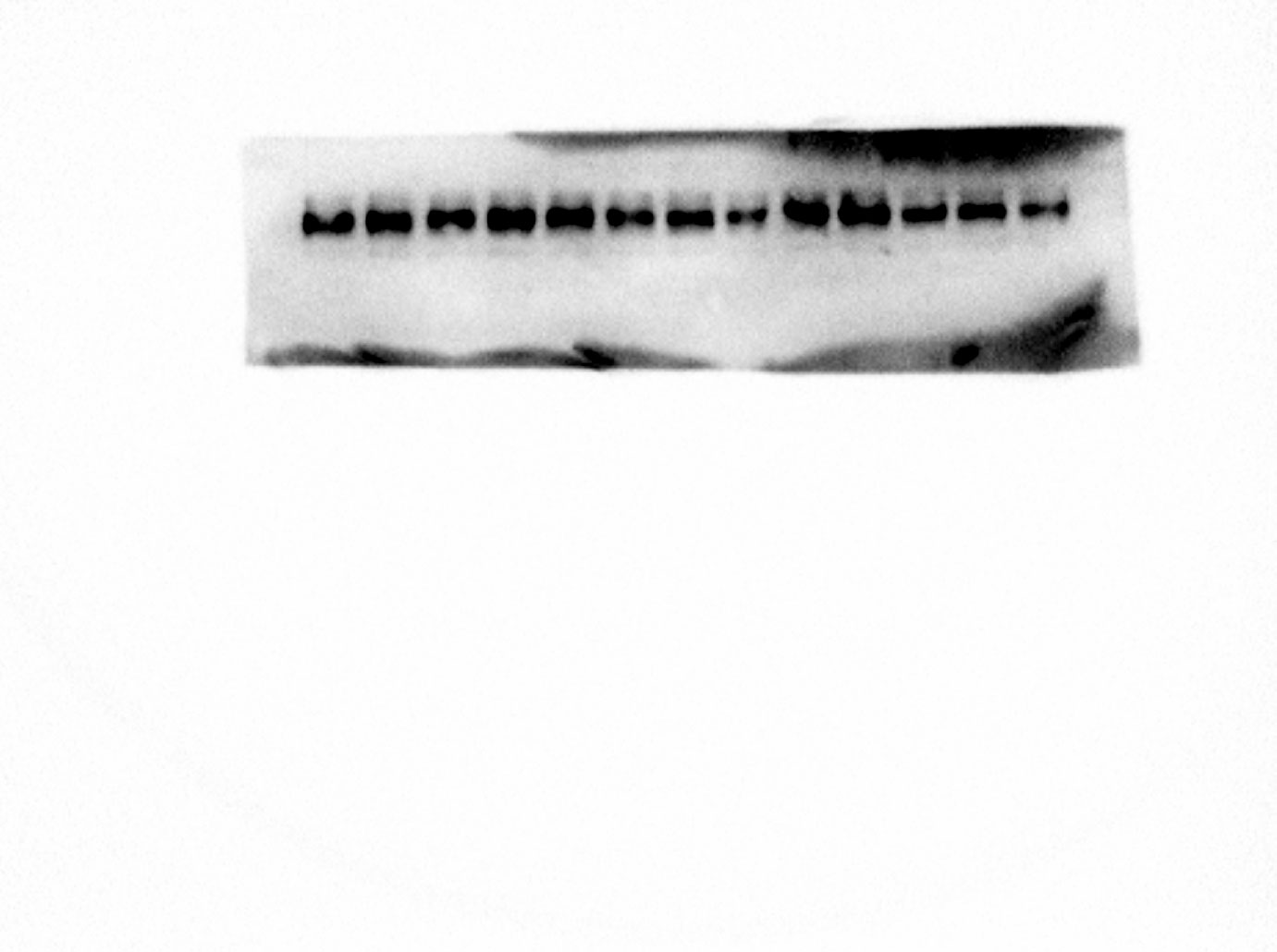

Supplement: Supplemental Information 16 — Raw data exported from western-blot for data analyses for Figs. 3C, 4 and Figs. S6–S8. [file peerj-07-7234-s016.zip › Western blot raw data figure 3C 4 s6 s7 s8/Figure 4 raw data/C4/C HIS.jpg]

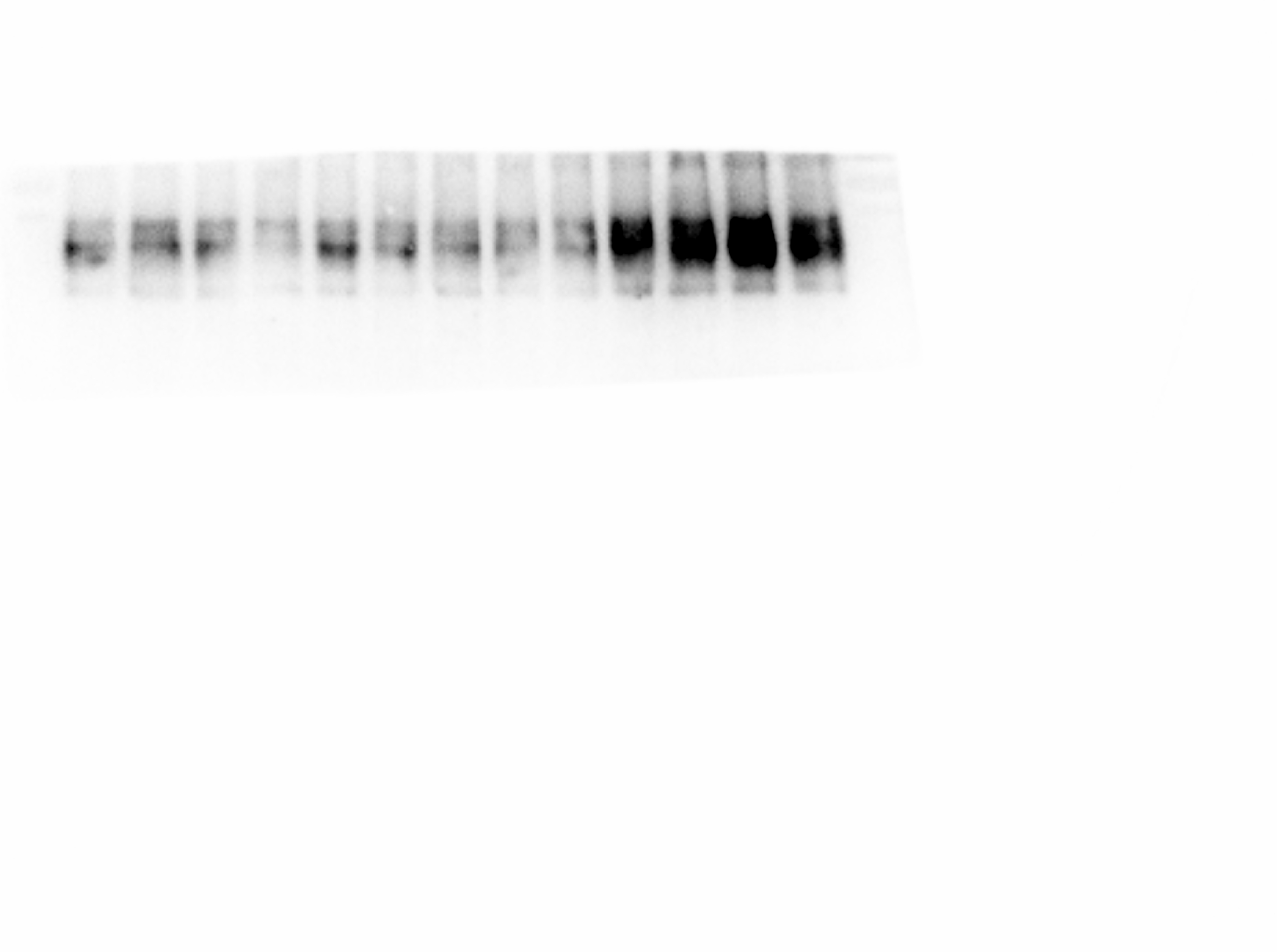

Supplement: Supplemental Information 16 — Raw data exported from western-blot for data analyses for Figs. 3C, 4 and Figs. S6–S8. [file peerj-07-7234-s016.zip › Western blot raw data figure 3C 4 s6 s7 s8/Figure 4 raw data/C4/C P-653.jpg]

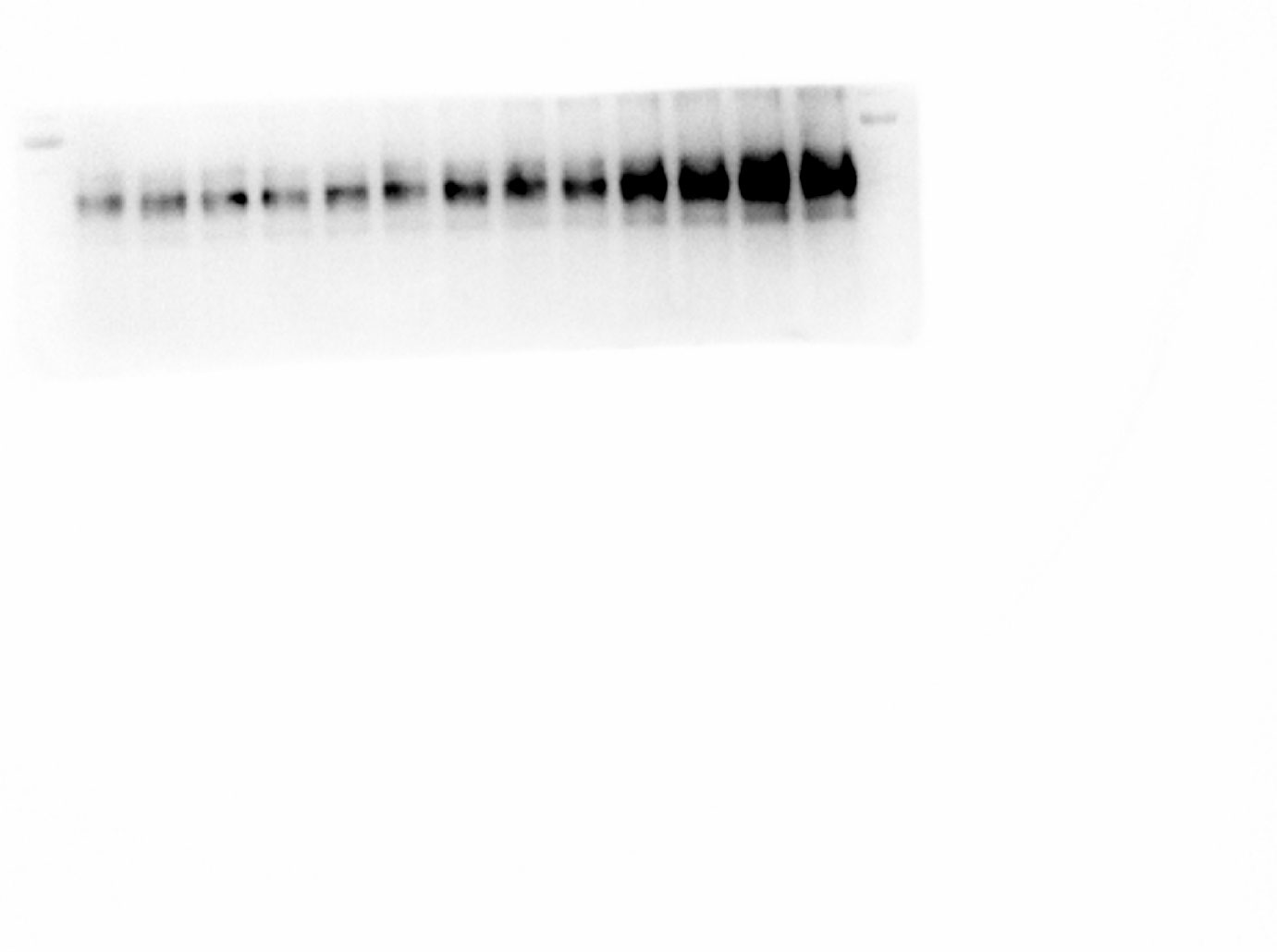

Supplement: Supplemental Information 16 — Raw data exported from western-blot for data analyses for Figs. 3C, 4 and Figs. S6–S8. [file peerj-07-7234-s016.zip › Western blot raw data figure 3C 4 s6 s7 s8/Figure 4 raw data/C4/C P-654.jpg]

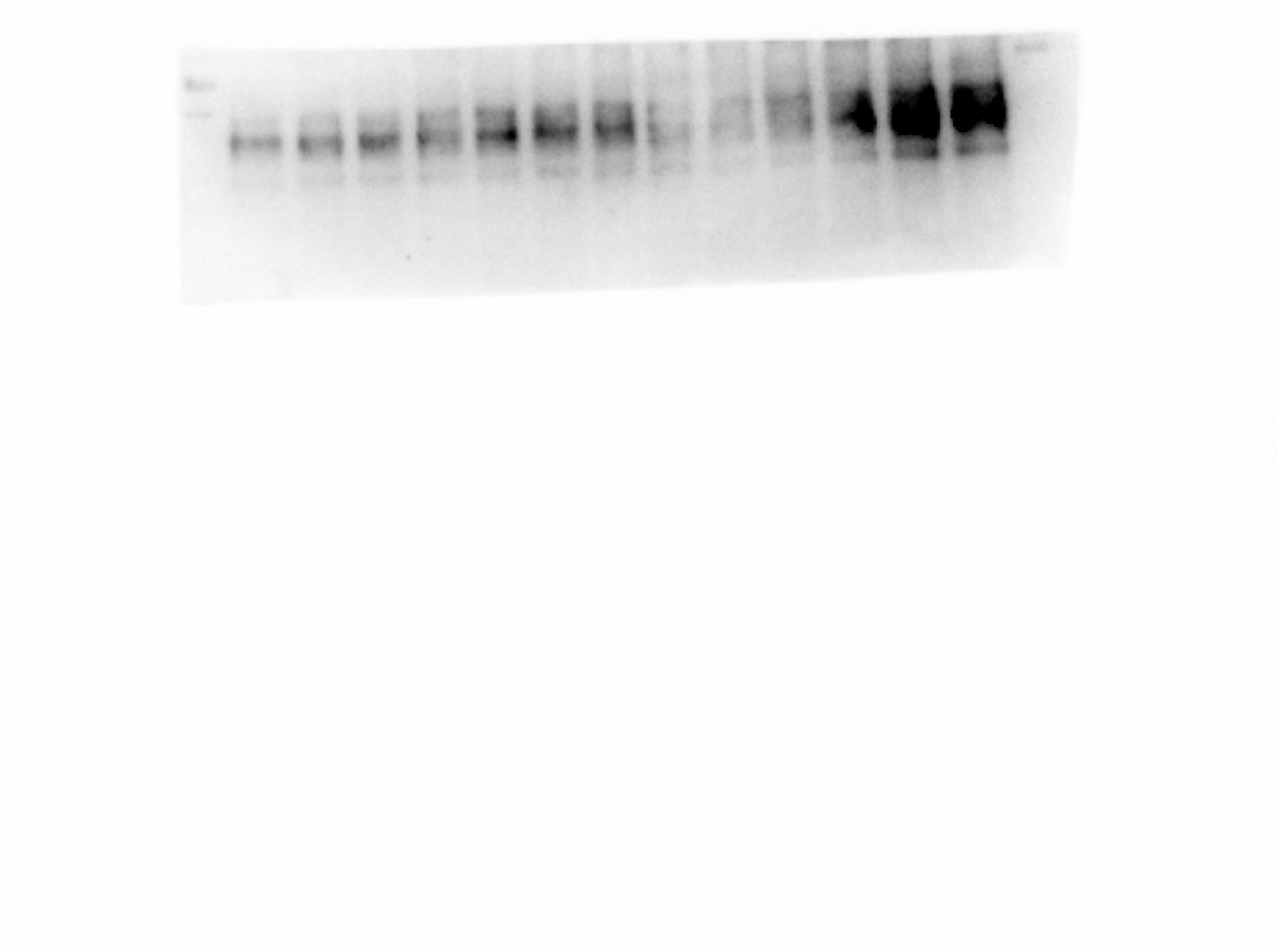

Supplement: Supplemental Information 16 — Raw data exported from western-blot for data analyses for Figs. 3C, 4 and Figs. S6–S8. [file peerj-07-7234-s016.zip › Western blot raw data figure 3C 4 s6 s7 s8/Figure 4 raw data/C4/C PY.jpg]

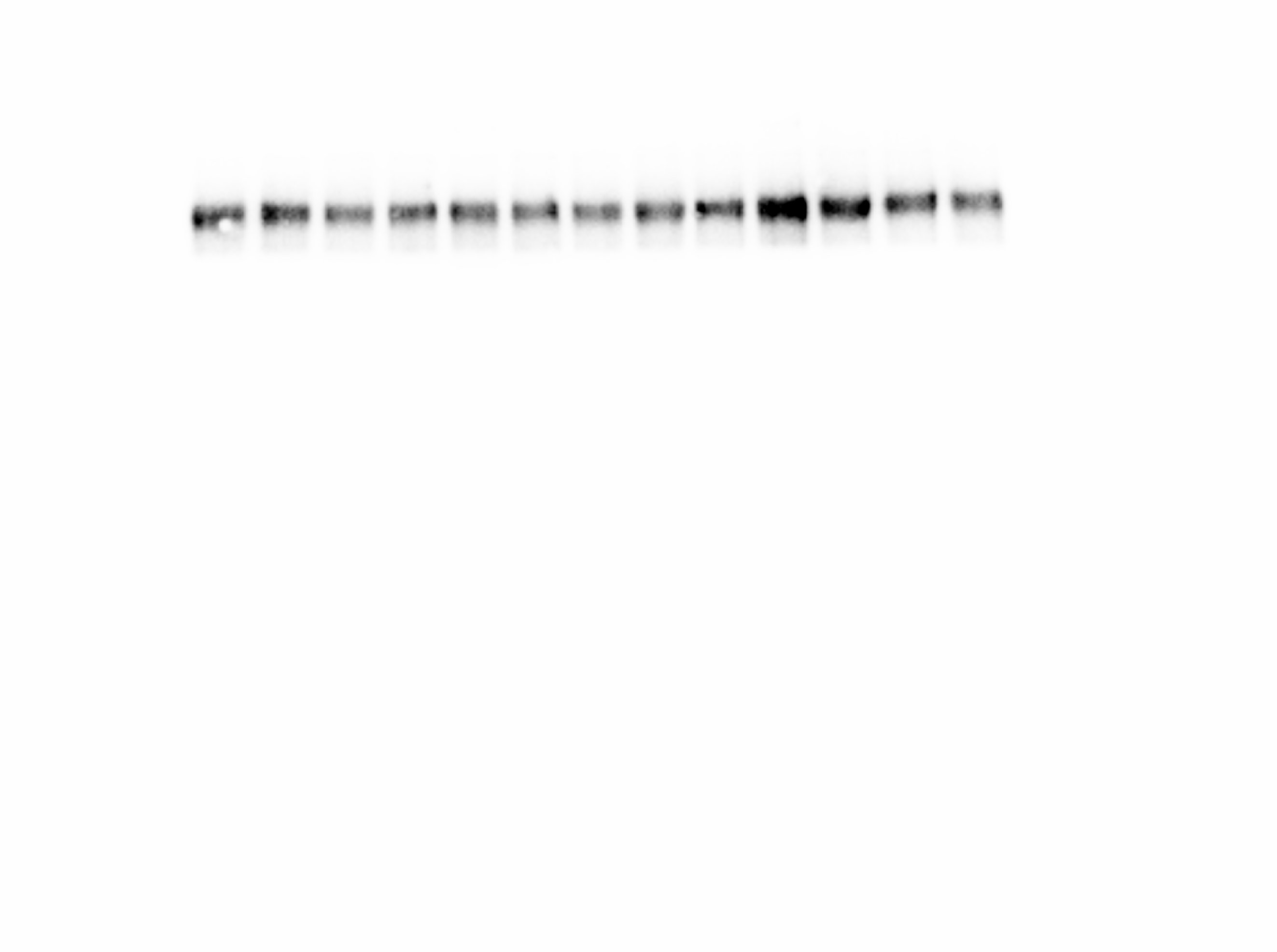

Supplement: Supplemental Information 16 — Raw data exported from western-blot for data analyses for Figs. 3C, 4 and Figs. S6–S8. [file peerj-07-7234-s016.zip › Western blot raw data figure 3C 4 s6 s7 s8/Figure 4 raw data/C5/1C P653.jpg]

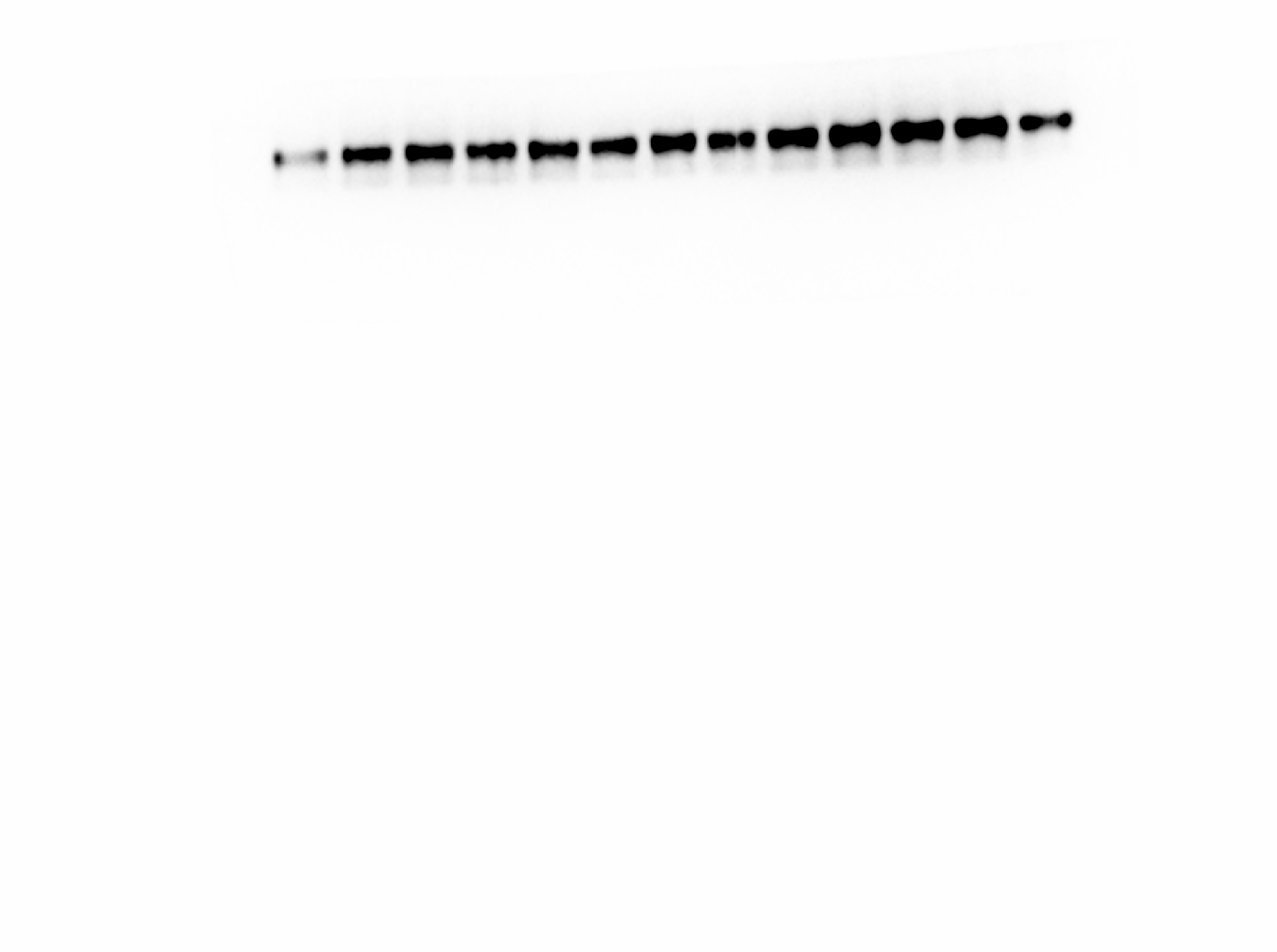

Supplement: Supplemental Information 16 — Raw data exported from western-blot for data analyses for Figs. 3C, 4 and Figs. S6–S8. [file peerj-07-7234-s016.zip › Western blot raw data figure 3C 4 s6 s7 s8/Figure 4 raw data/C5/2C P654.jpg]

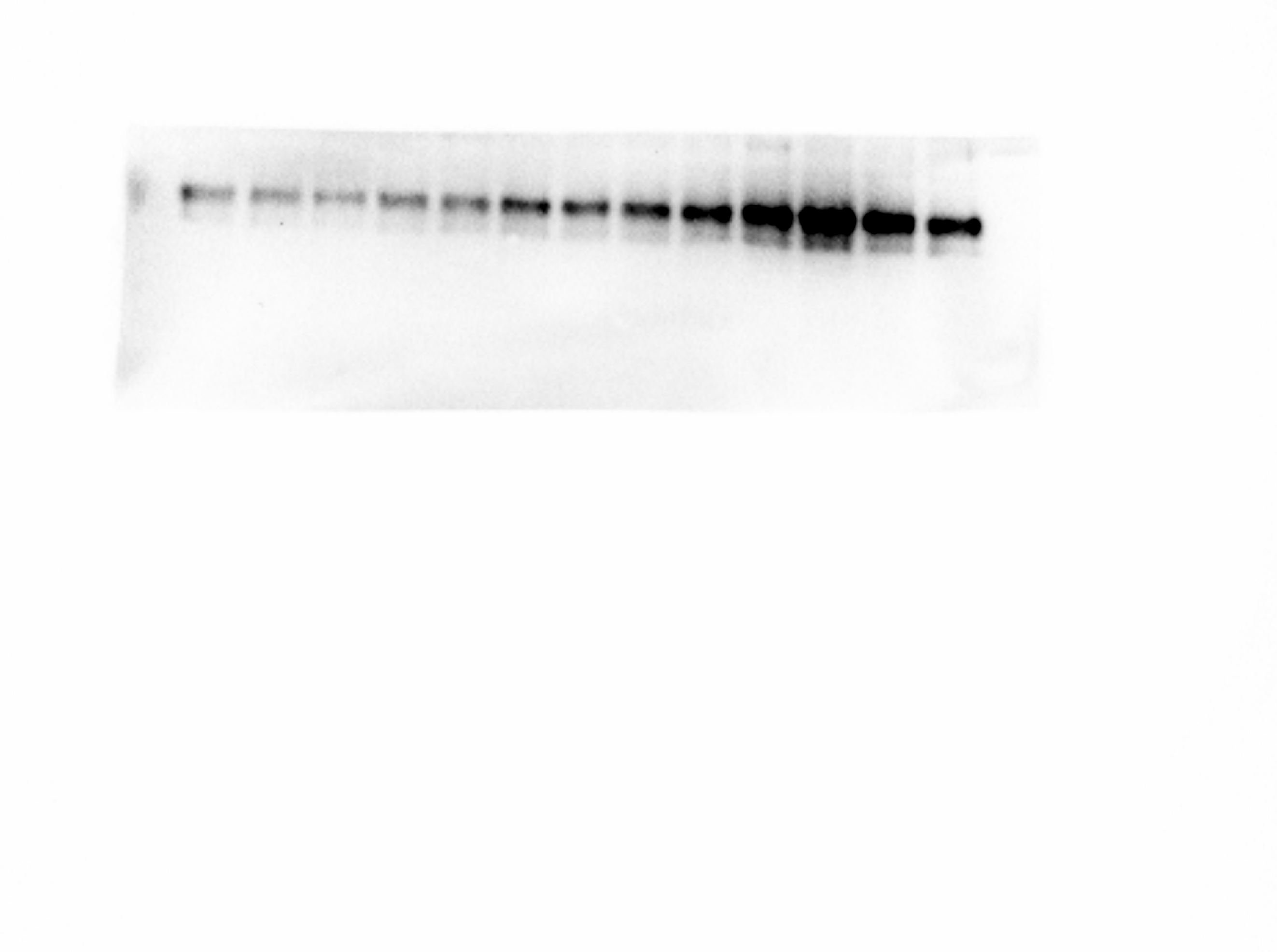

Supplement: Supplemental Information 16 — Raw data exported from western-blot for data analyses for Figs. 3C, 4 and Figs. S6–S8. [file peerj-07-7234-s016.zip › Western blot raw data figure 3C 4 s6 s7 s8/Figure 4 raw data/C5/2C PY.jpg]

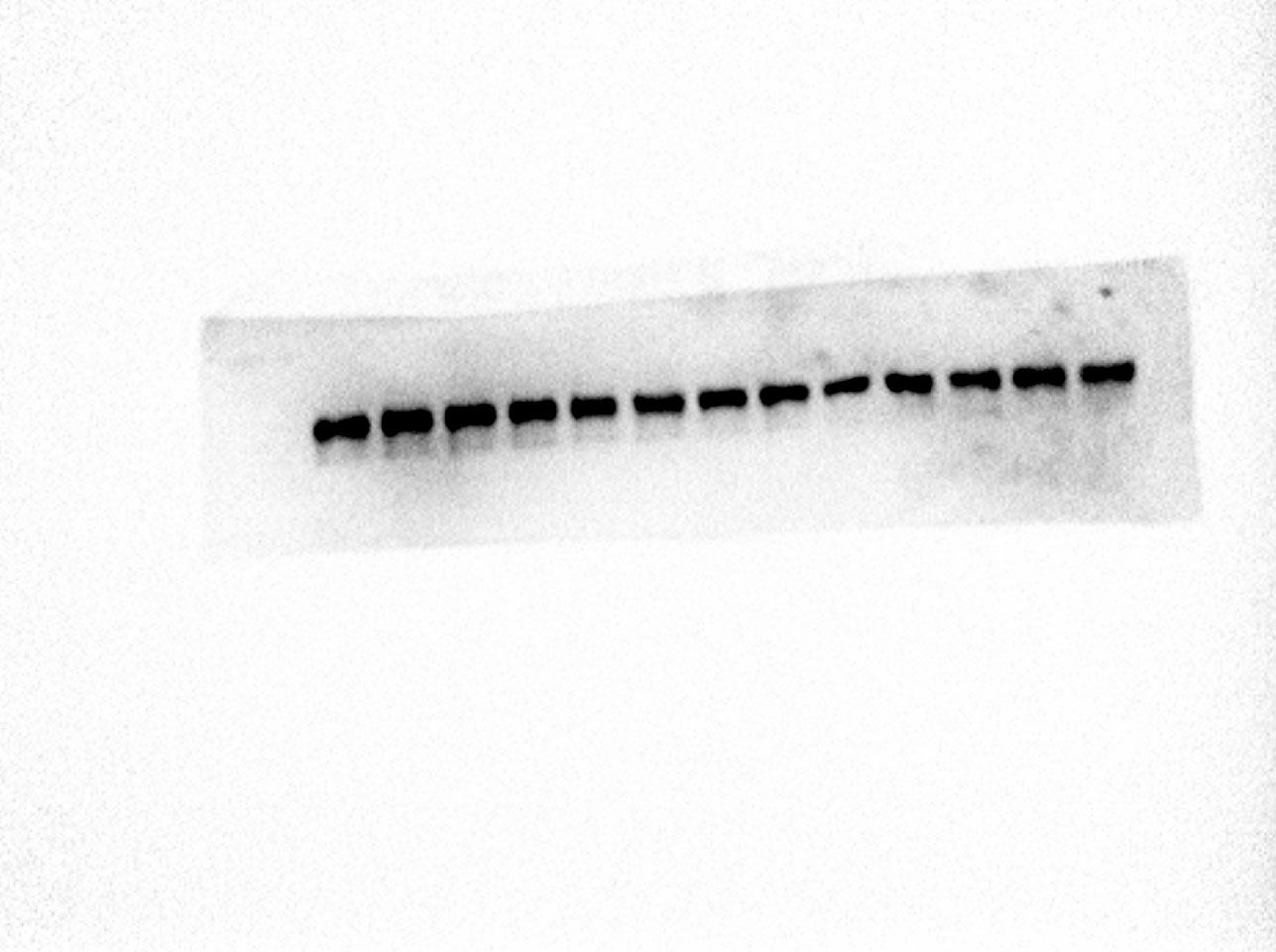

Supplement: Supplemental Information 16 — Raw data exported from western-blot for data analyses for Figs. 3C, 4 and Figs. S6–S8. [file peerj-07-7234-s016.zip › Western blot raw data figure 3C 4 s6 s7 s8/Figure 4 raw data/C5/C HIS.jpg]

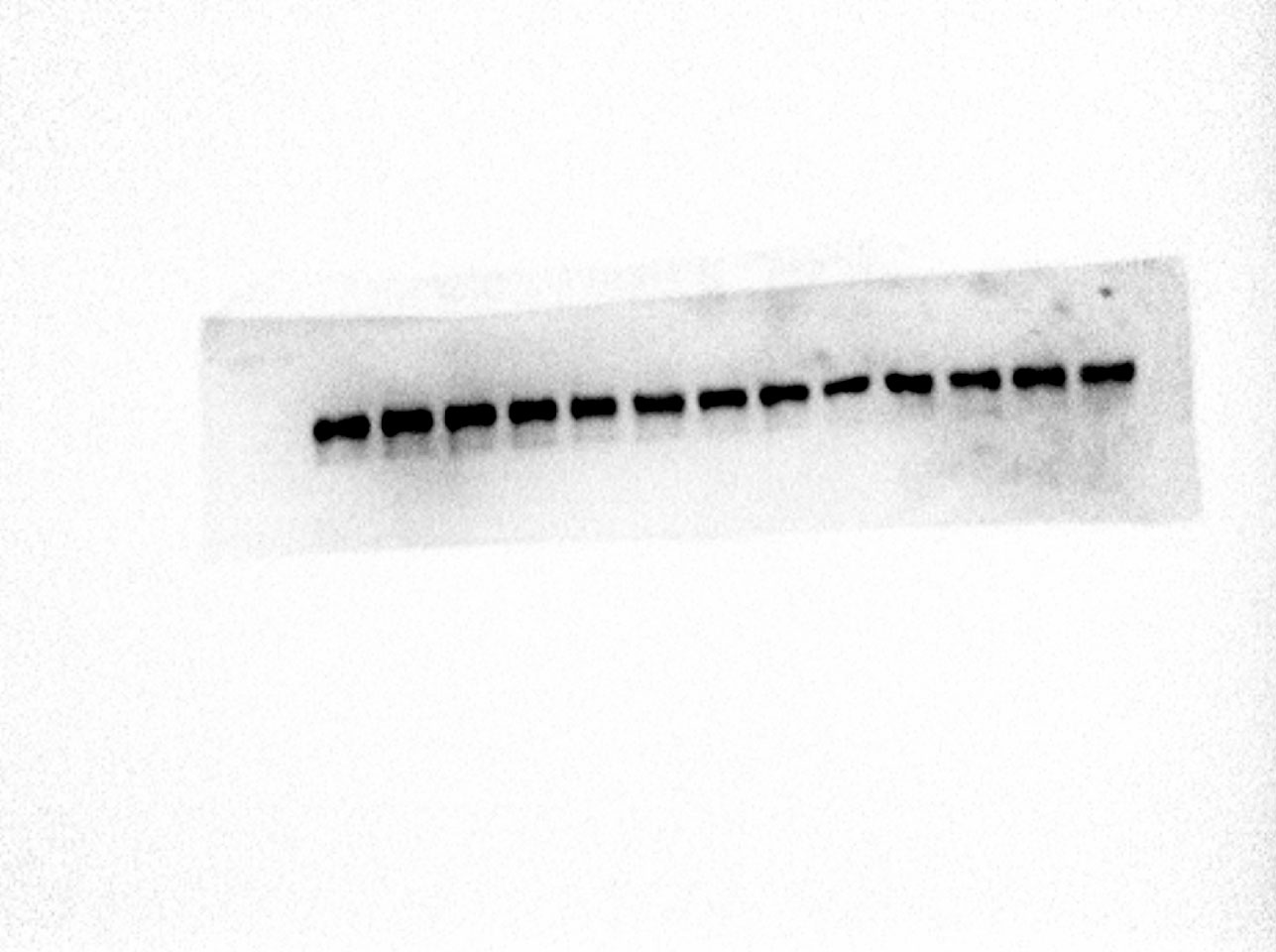

Supplement: Supplemental Information 16 — Raw data exported from western-blot for data analyses for Figs. 3C, 4 and Figs. S6–S8. [file peerj-07-7234-s016.zip › Western blot raw data figure 3C 4 s6 s7 s8/Figure 4 raw data/C6/C HIS.jpg]

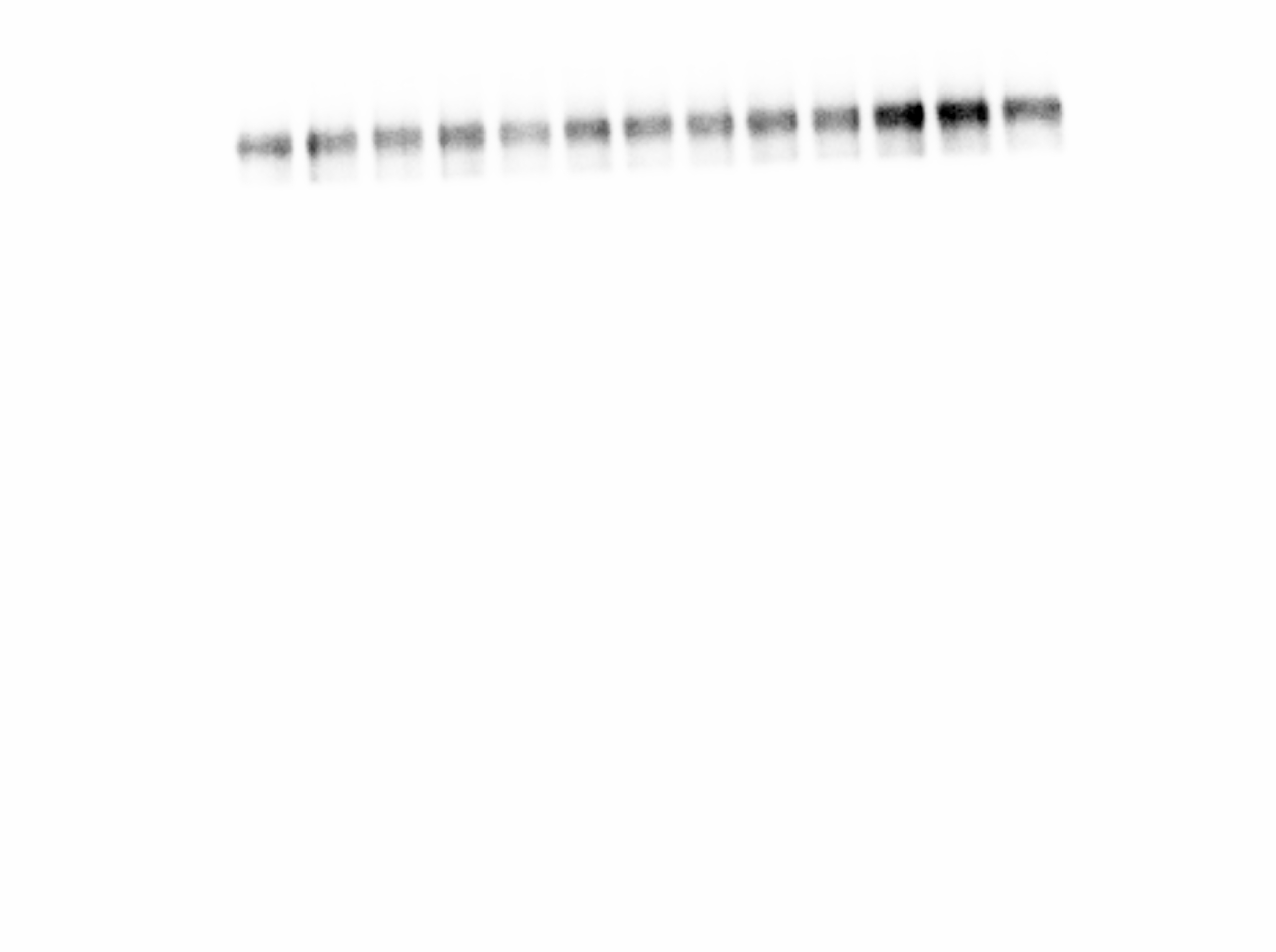

Supplement: Supplemental Information 16 — Raw data exported from western-blot for data analyses for Figs. 3C, 4 and Figs. S6–S8. [file peerj-07-7234-s016.zip › Western blot raw data figure 3C 4 s6 s7 s8/Figure 4 raw data/C6/C P653.jpg]

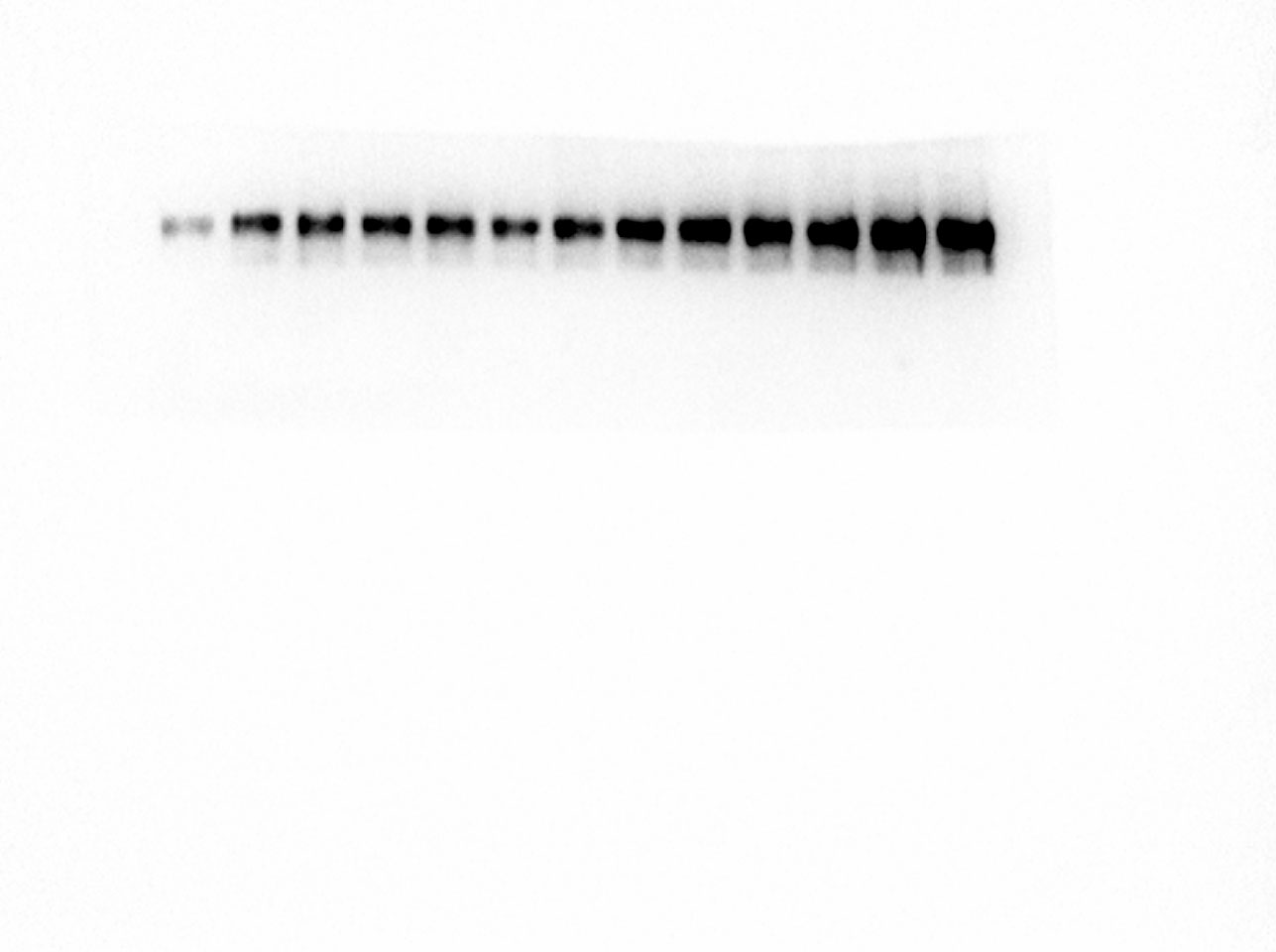

Supplement: Supplemental Information 16 — Raw data exported from western-blot for data analyses for Figs. 3C, 4 and Figs. S6–S8. [file peerj-07-7234-s016.zip › Western blot raw data figure 3C 4 s6 s7 s8/Figure 4 raw data/C6/C P654.jpg]

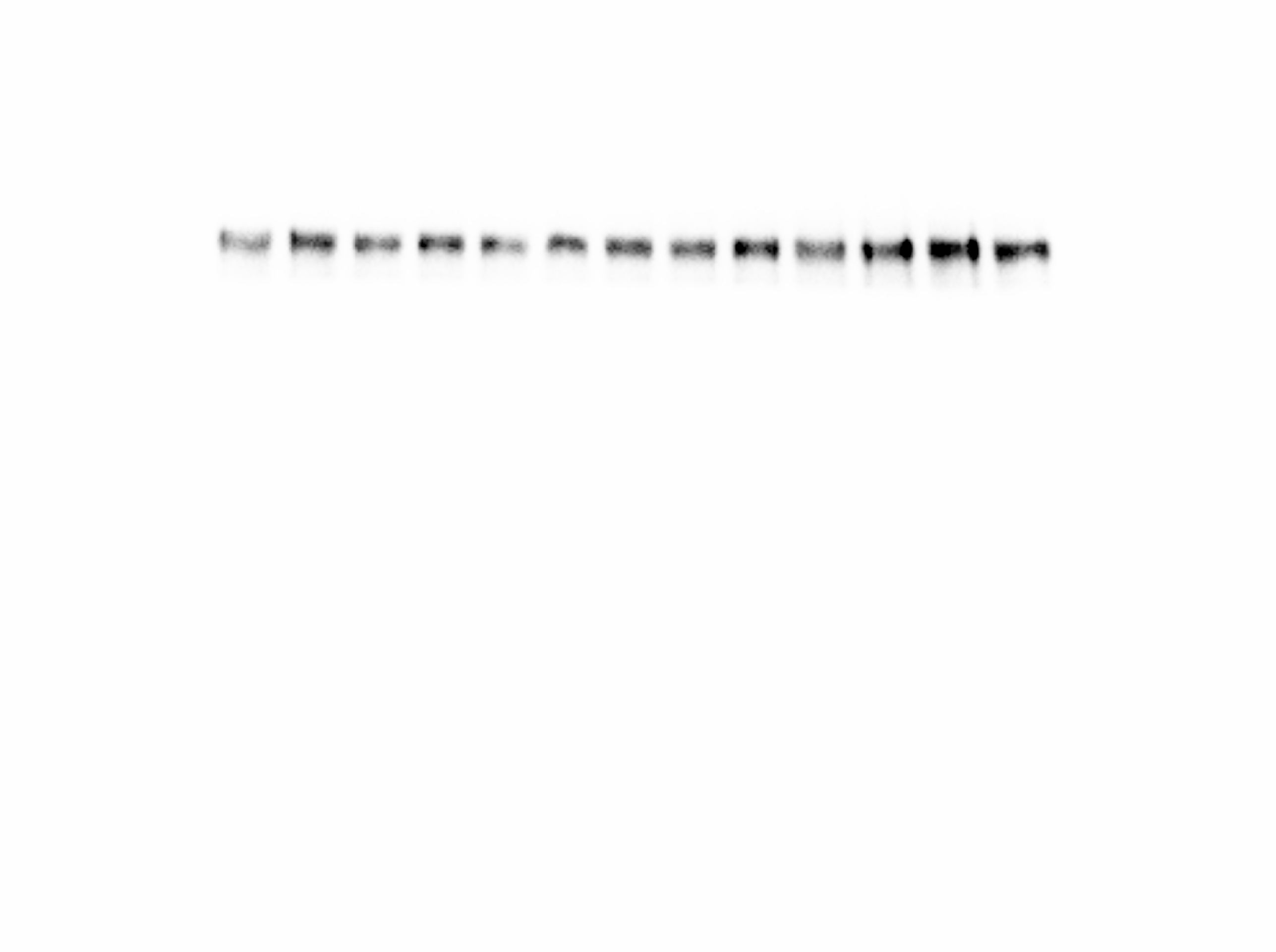

Supplement: Supplemental Information 16 — Raw data exported from western-blot for data analyses for Figs. 3C, 4 and Figs. S6–S8. [file peerj-07-7234-s016.zip › Western blot raw data figure 3C 4 s6 s7 s8/Figure 4 raw data/C6/C PY.jpg]

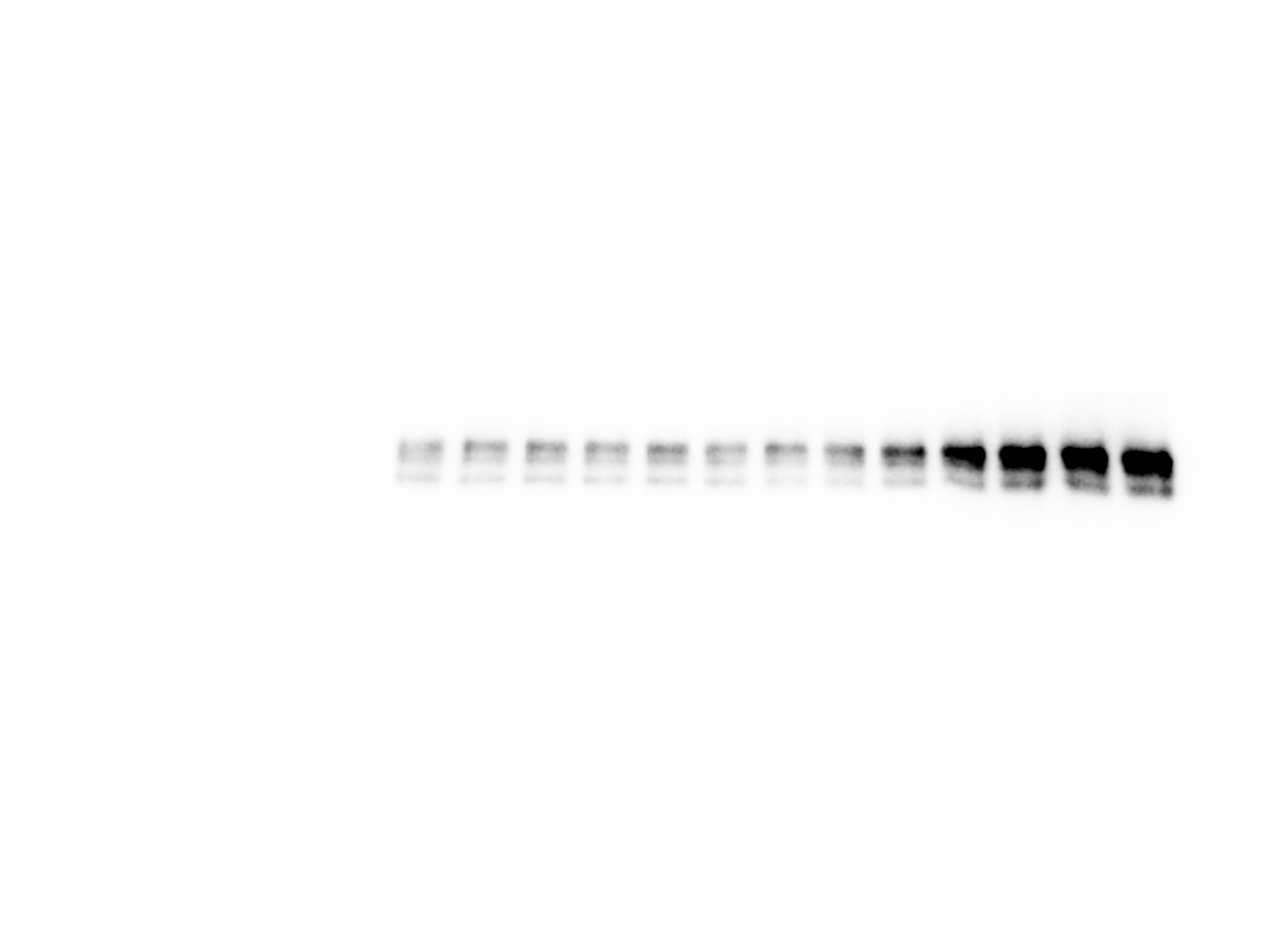

Supplement: Supplemental Information 16 — Raw data exported from western-blot for data analyses for Figs. 3C, 4 and Figs. S6–S8. [file peerj-07-7234-s016.zip › Western blot raw data figure 3C 4 s6 s7 s8/Figure 4 raw data/N1/N -P653.jpg]

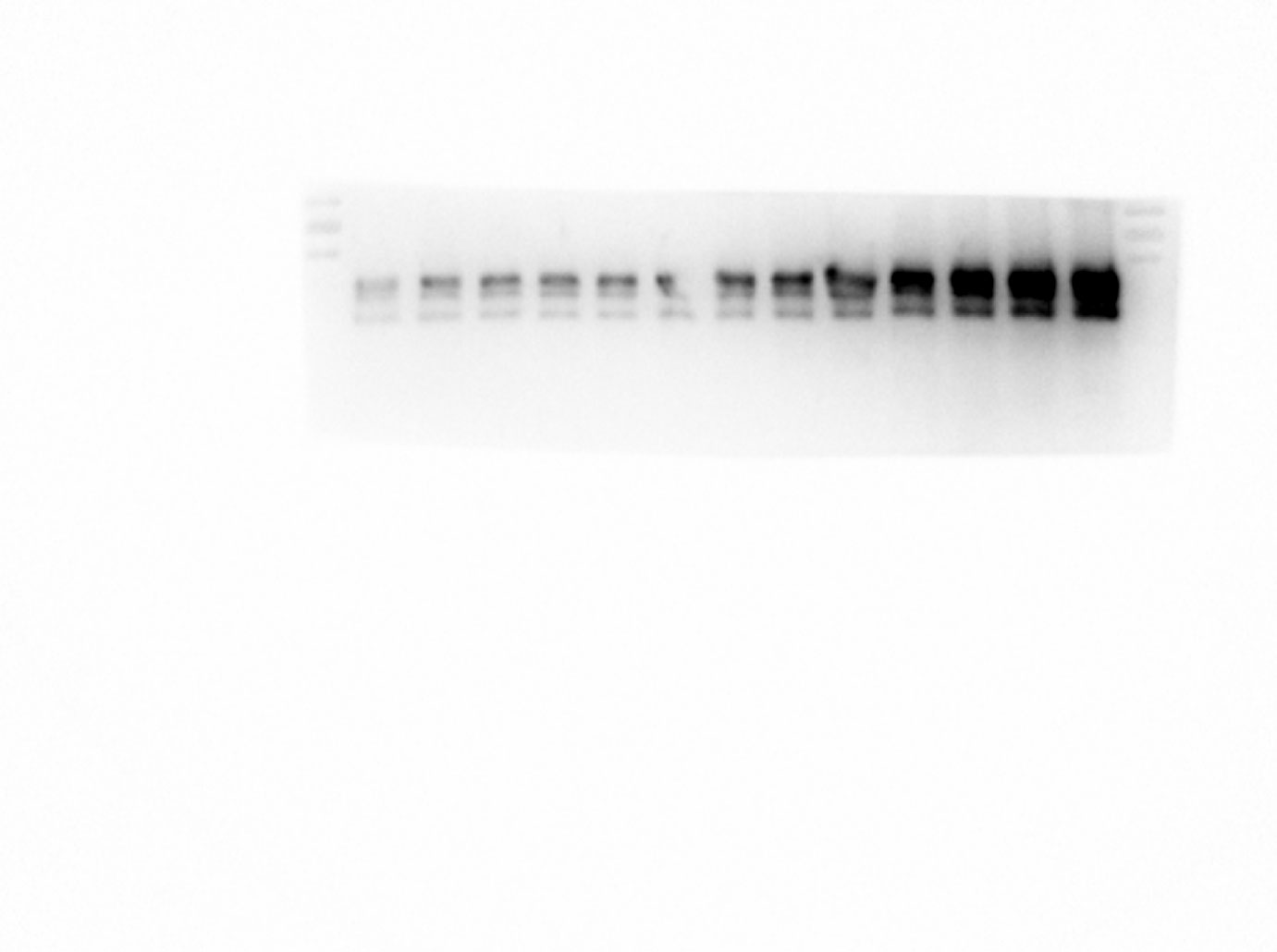

Supplement: Supplemental Information 16 — Raw data exported from western-blot for data analyses for Figs. 3C, 4 and Figs. S6–S8. [file peerj-07-7234-s016.zip › Western blot raw data figure 3C 4 s6 s7 s8/Figure 4 raw data/N1/N -P654.jpg]

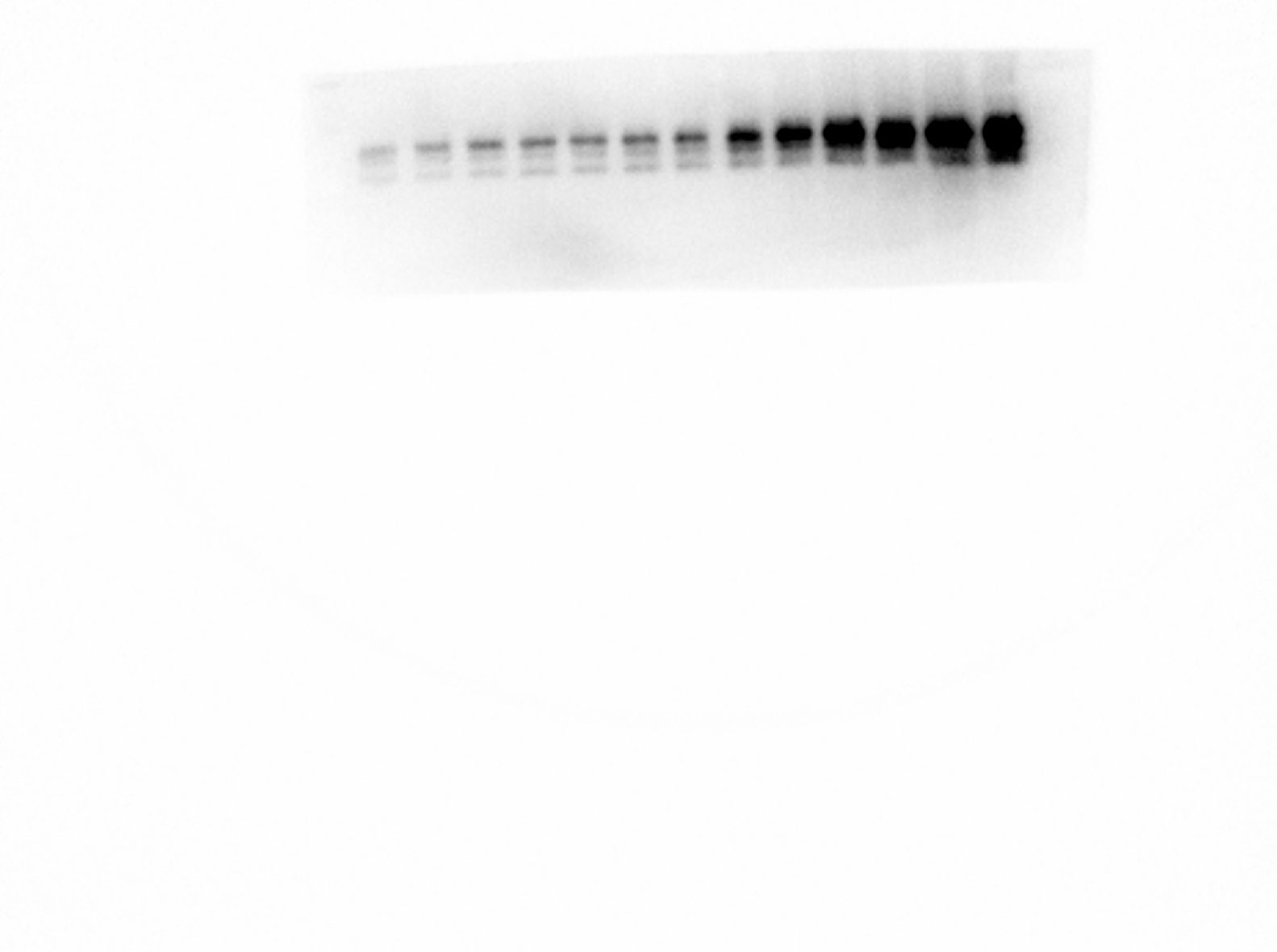

Supplement: Supplemental Information 16 — Raw data exported from western-blot for data analyses for Figs. 3C, 4 and Figs. S6–S8. [file peerj-07-7234-s016.zip › Western blot raw data figure 3C 4 s6 s7 s8/Figure 4 raw data/N1/N -PY.jpg]

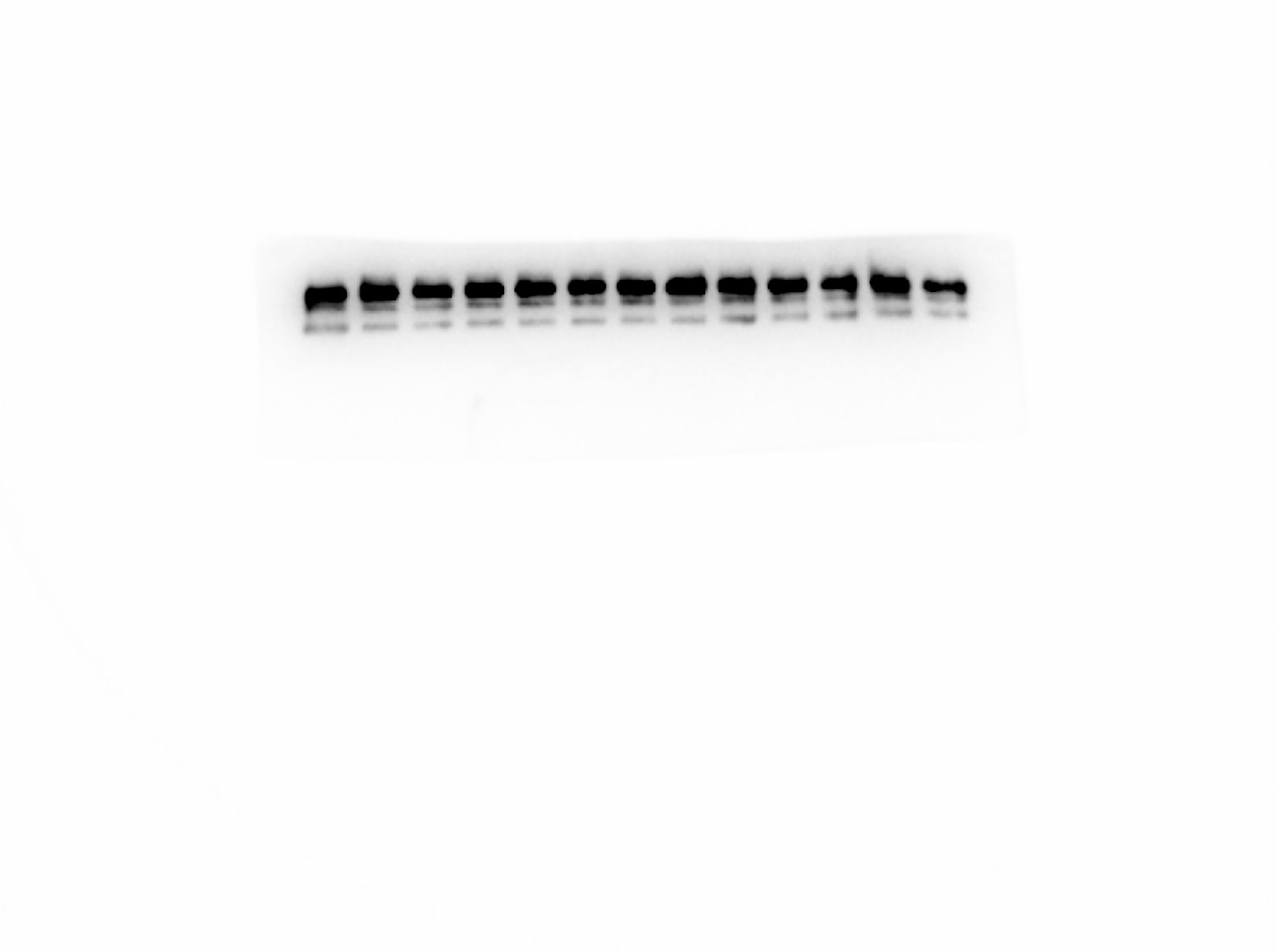

Supplement: Supplemental Information 16 — Raw data exported from western-blot for data analyses for Figs. 3C, 4 and Figs. S6–S8. [file peerj-07-7234-s016.zip › Western blot raw data figure 3C 4 s6 s7 s8/Figure 4 raw data/N1/N-HIS.jpg]

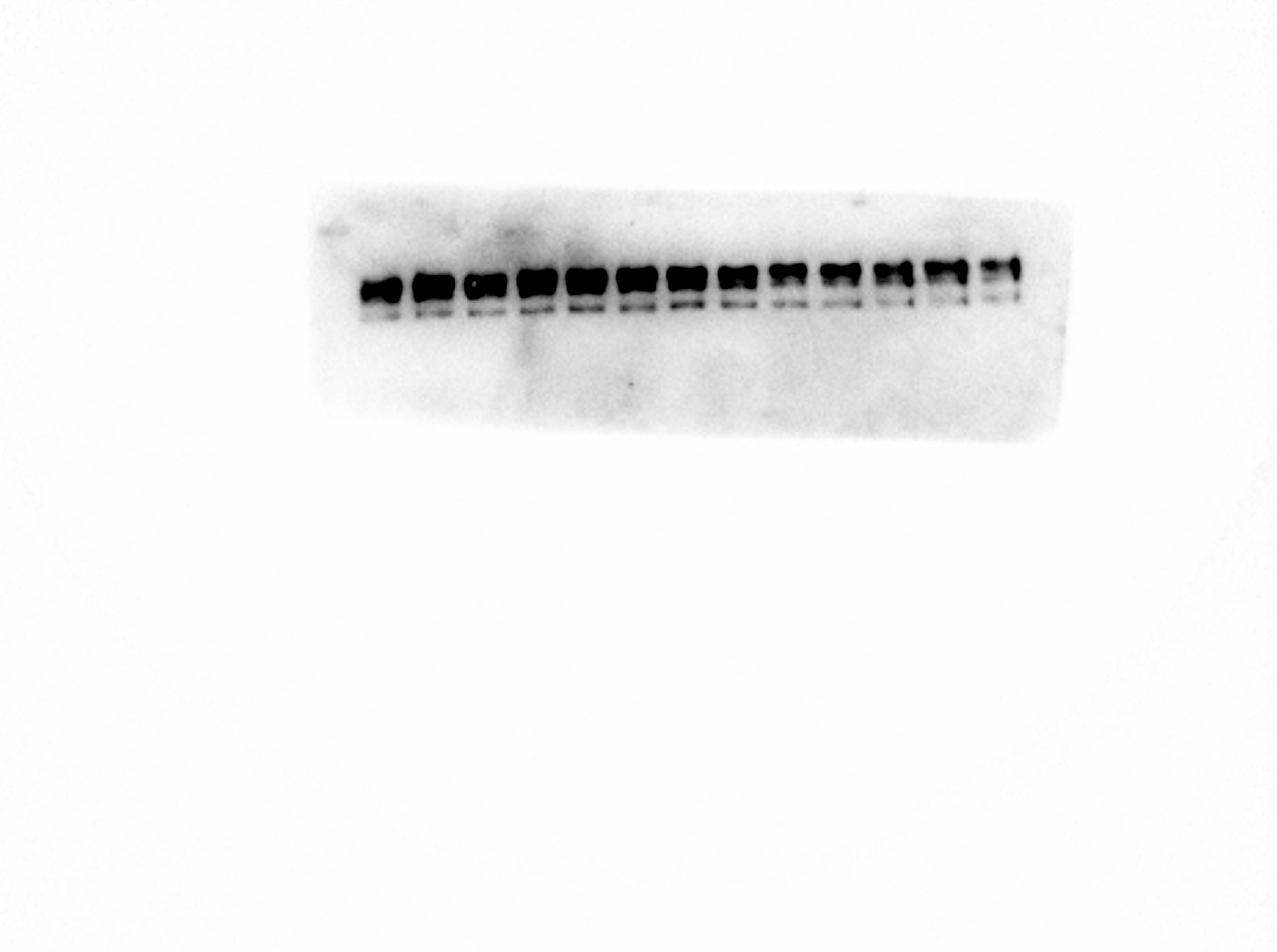

Supplement: Supplemental Information 16 — Raw data exported from western-blot for data analyses for Figs. 3C, 4 and Figs. S6–S8. [file peerj-07-7234-s016.zip › Western blot raw data figure 3C 4 s6 s7 s8/Figure 4 raw data/N2/HIS.jpg]

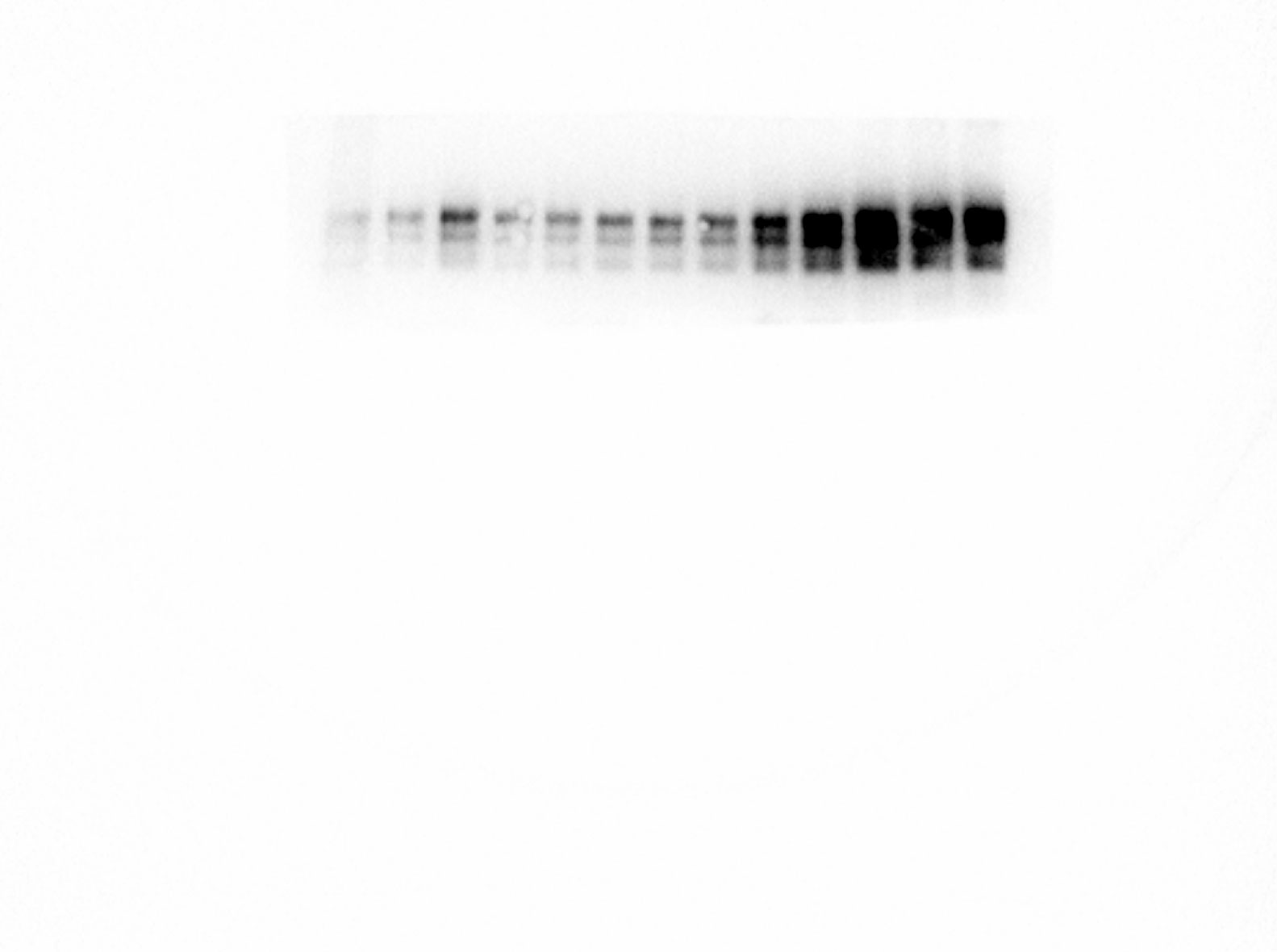

Supplement: Supplemental Information 16 — Raw data exported from western-blot for data analyses for Figs. 3C, 4 and Figs. S6–S8. [file peerj-07-7234-s016.zip › Western blot raw data figure 3C 4 s6 s7 s8/Figure 4 raw data/N2/n PY.jpg]

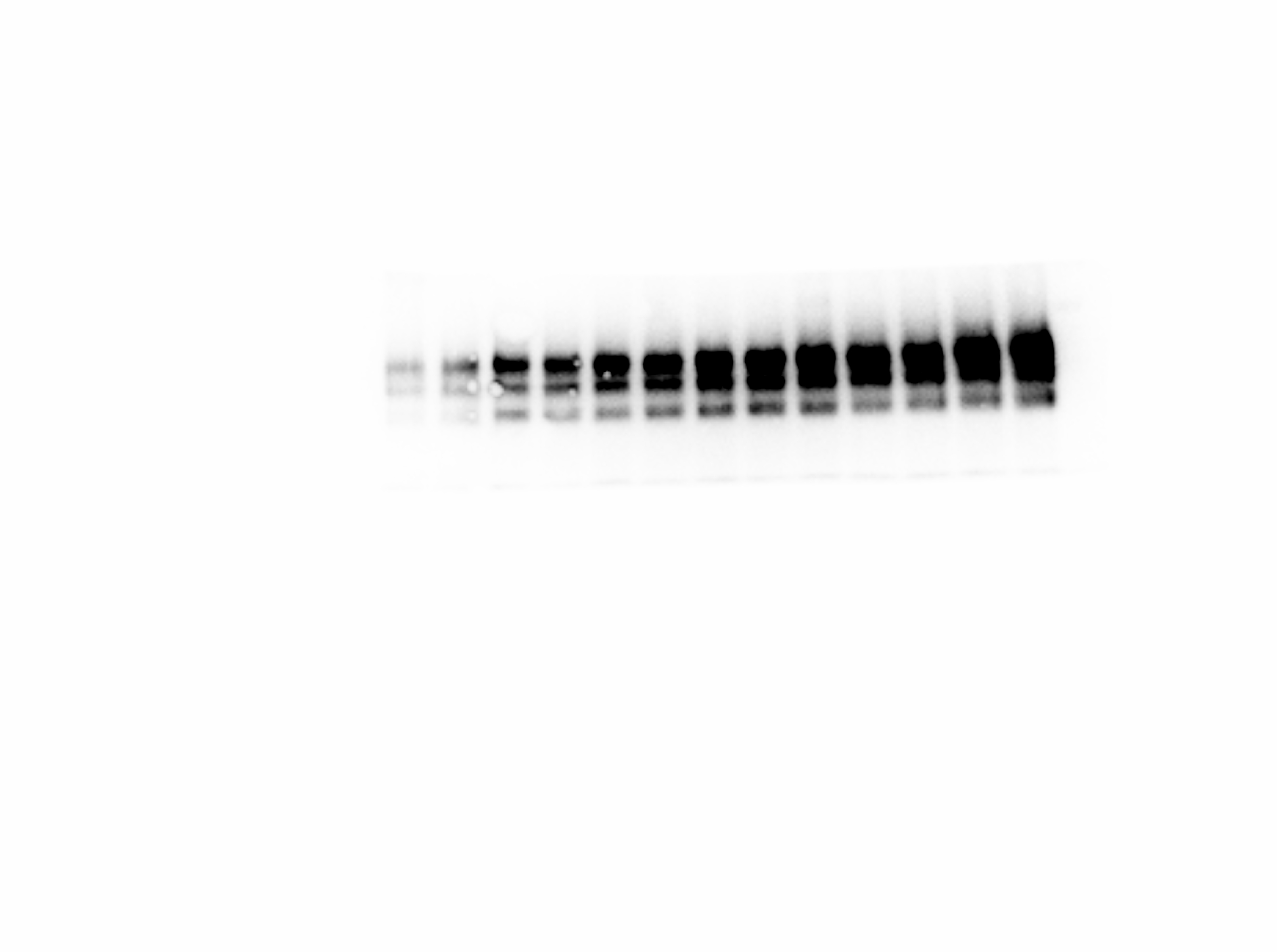

Supplement: Supplemental Information 16 — Raw data exported from western-blot for data analyses for Figs. 3C, 4 and Figs. S6–S8. [file peerj-07-7234-s016.zip › Western blot raw data figure 3C 4 s6 s7 s8/Figure 4 raw data/N2/n p653.jpg]

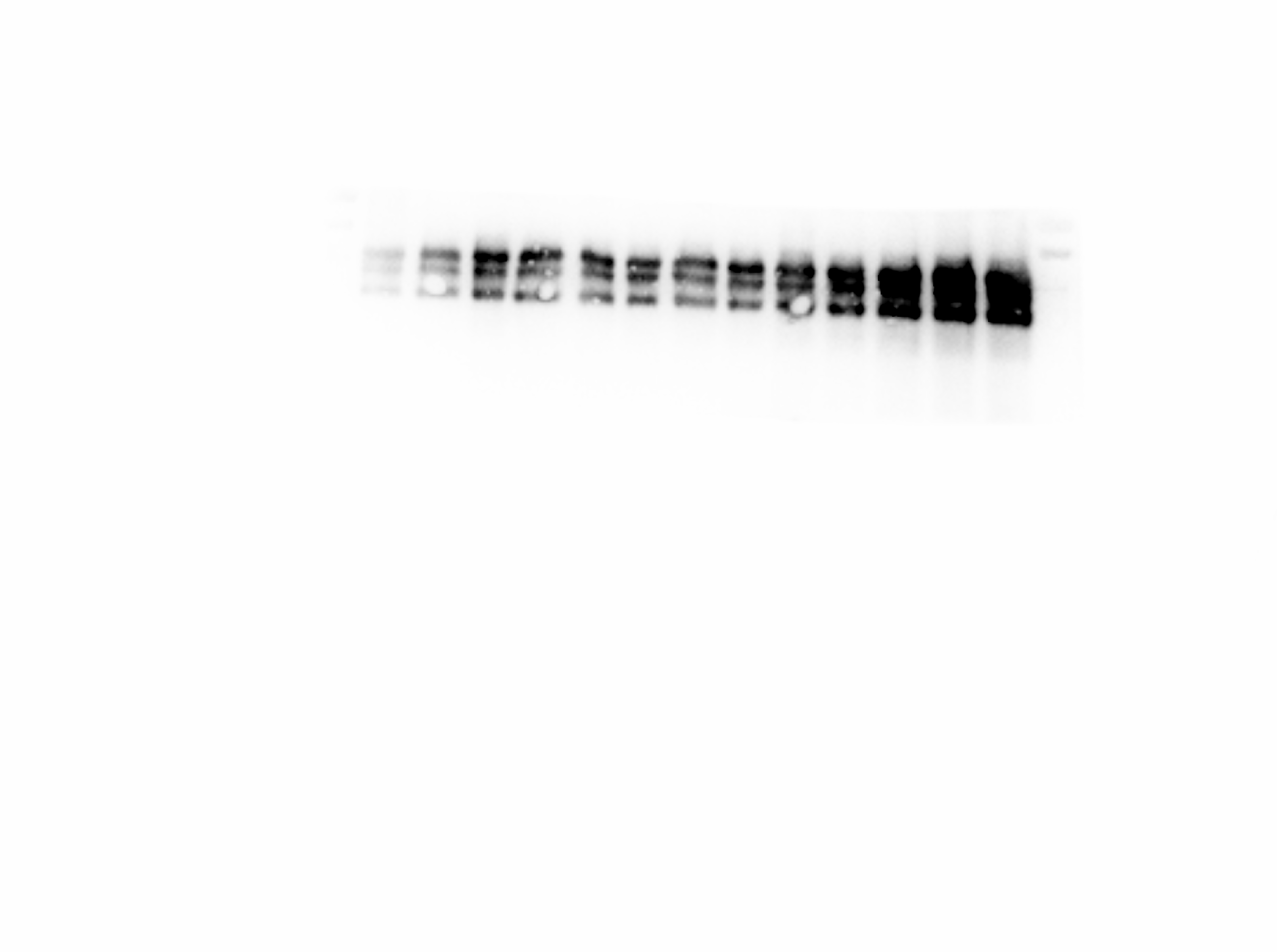

Supplement: Supplemental Information 16 — Raw data exported from western-blot for data analyses for Figs. 3C, 4 and Figs. S6–S8. [file peerj-07-7234-s016.zip › Western blot raw data figure 3C 4 s6 s7 s8/Figure 4 raw data/N2/n p654.jpg]

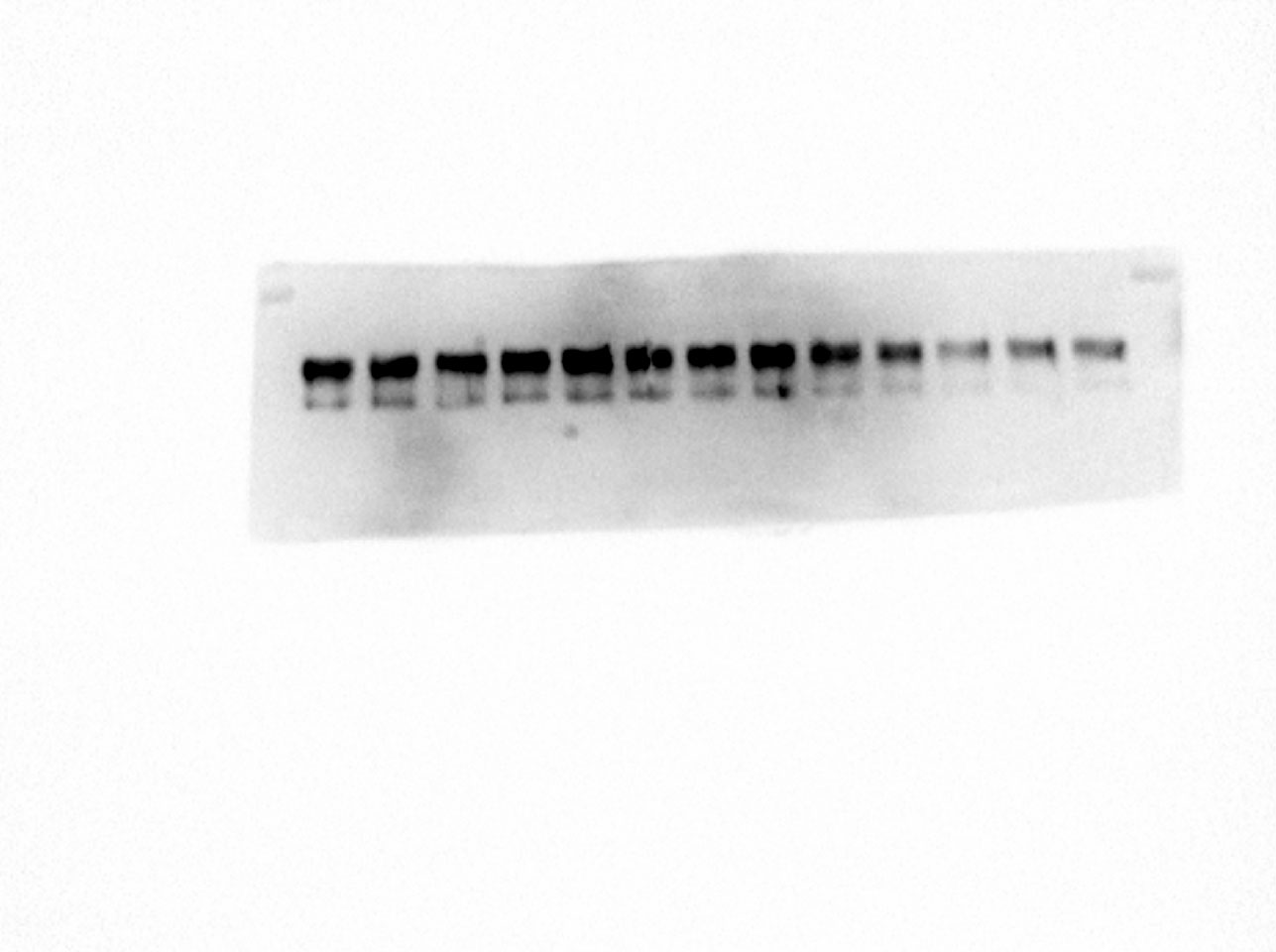

Supplement: Supplemental Information 16 — Raw data exported from western-blot for data analyses for Figs. 3C, 4 and Figs. S6–S8. [file peerj-07-7234-s016.zip › Western blot raw data figure 3C 4 s6 s7 s8/Figure 4 raw data/N3/N-HIS.jpg]

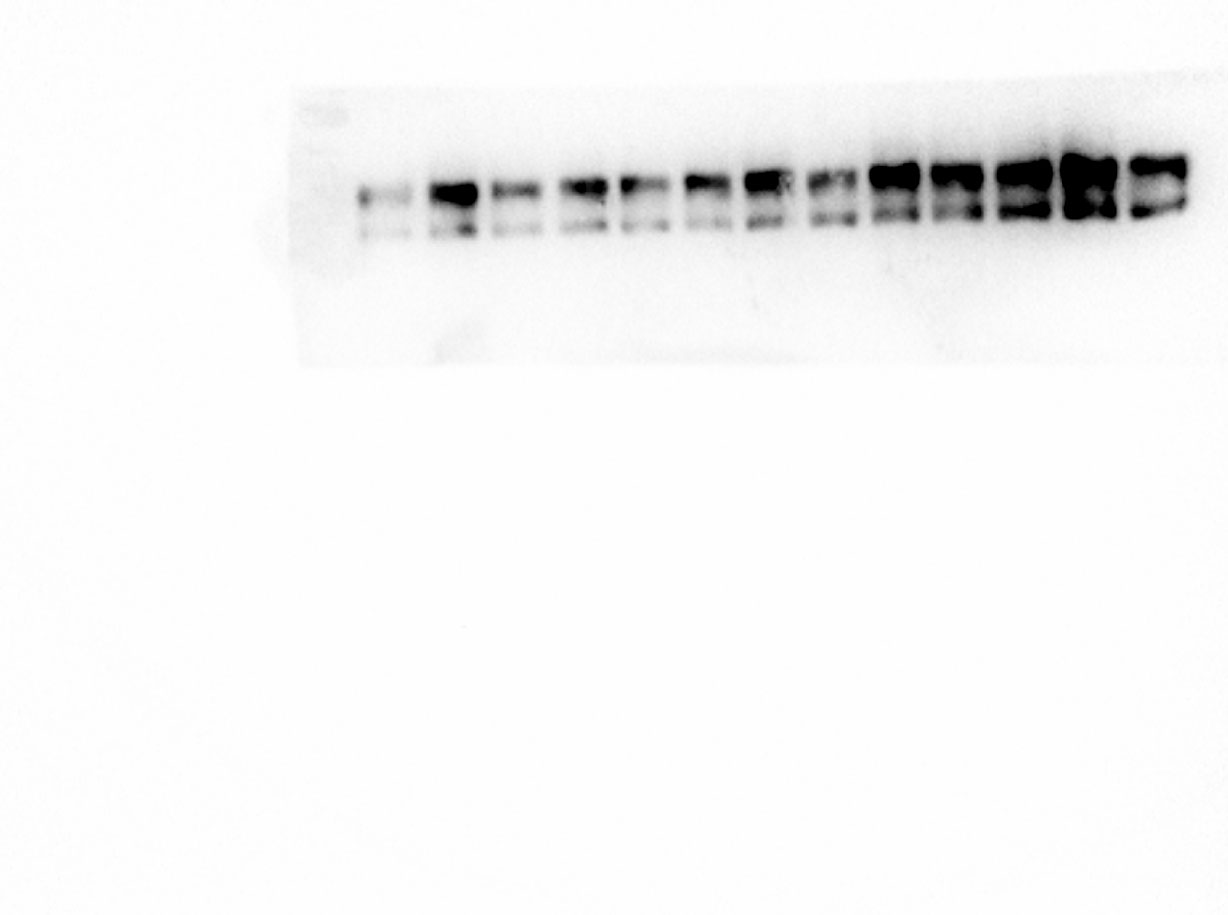

Supplement: Supplemental Information 16 — Raw data exported from western-blot for data analyses for Figs. 3C, 4 and Figs. S6–S8. [file peerj-07-7234-s016.zip › Western blot raw data figure 3C 4 s6 s7 s8/Figure 4 raw data/N3/N-P653.jpg]

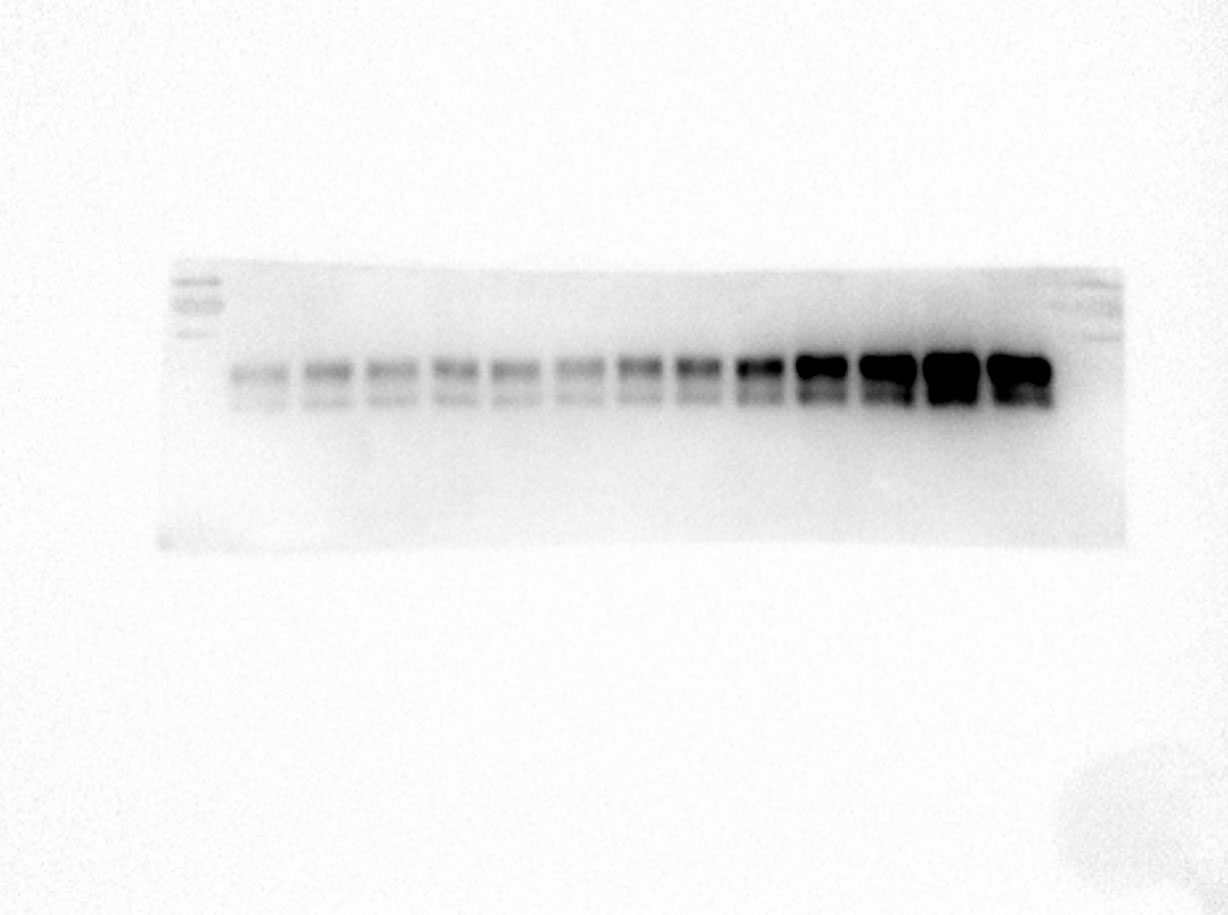

Supplement: Supplemental Information 16 — Raw data exported from western-blot for data analyses for Figs. 3C, 4 and Figs. S6–S8. [file peerj-07-7234-s016.zip › Western blot raw data figure 3C 4 s6 s7 s8/Figure 4 raw data/N3/N-P654.jpg]

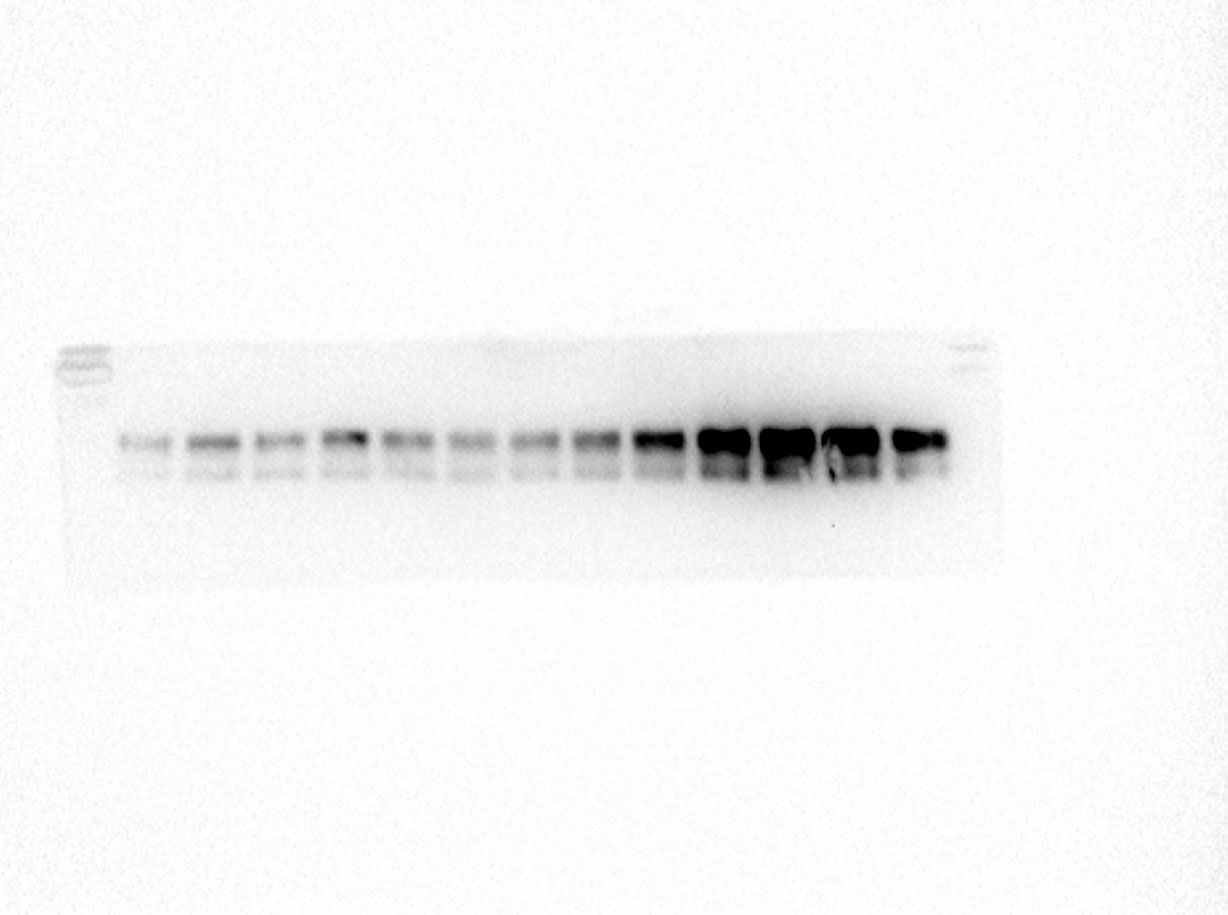

Supplement: Supplemental Information 16 — Raw data exported from western-blot for data analyses for Figs. 3C, 4 and Figs. S6–S8. [file peerj-07-7234-s016.zip › Western blot raw data figure 3C 4 s6 s7 s8/Figure 4 raw data/N3/N-PY.jpg]

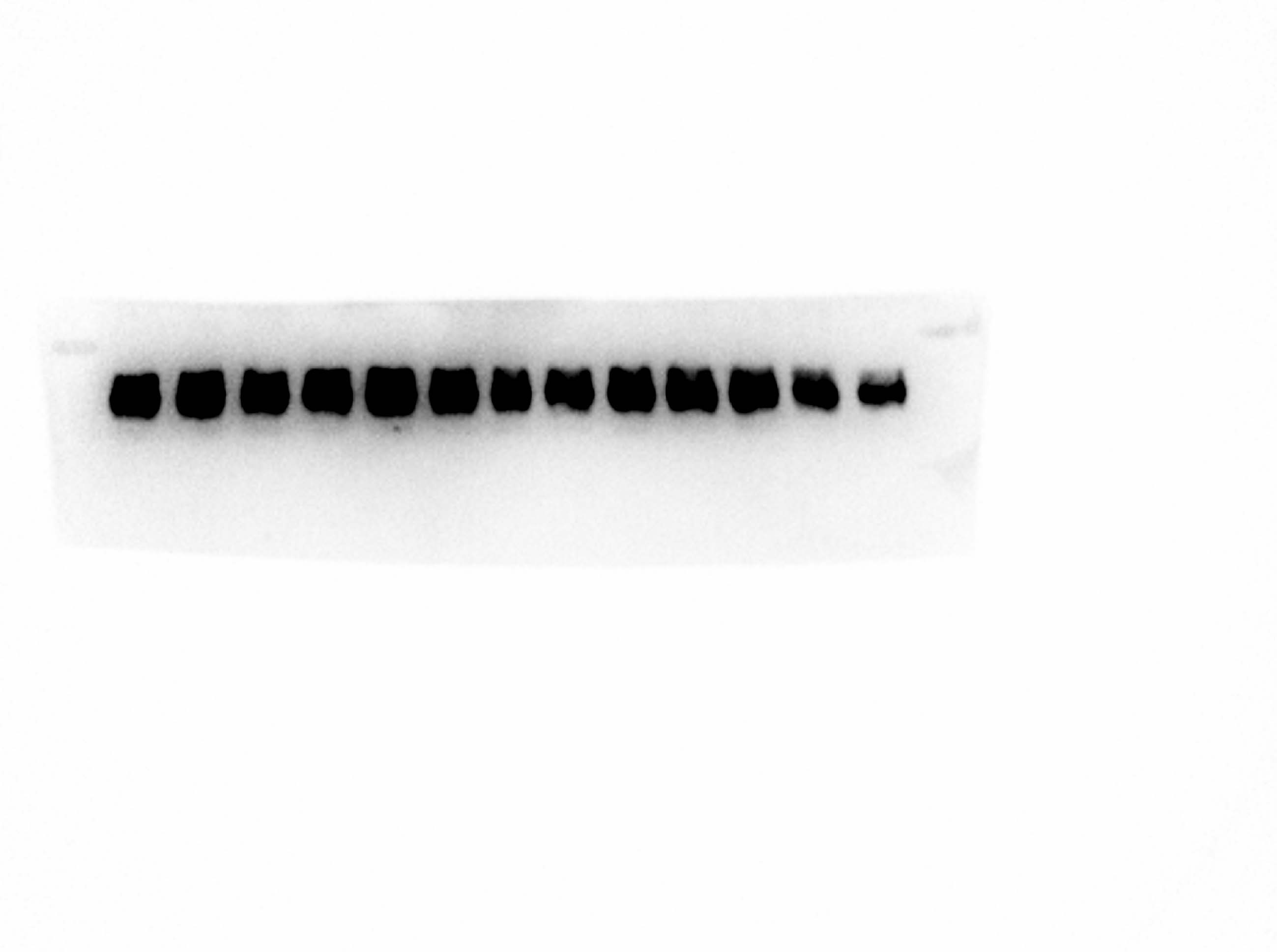

Supplement: Supplemental Information 16 — Raw data exported from western-blot for data analyses for Figs. 3C, 4 and Figs. S6–S8. [file peerj-07-7234-s016.zip › Western blot raw data figure 3C 4 s6 s7 s8/Figure 4 raw data/N4/N HIS.jpg]

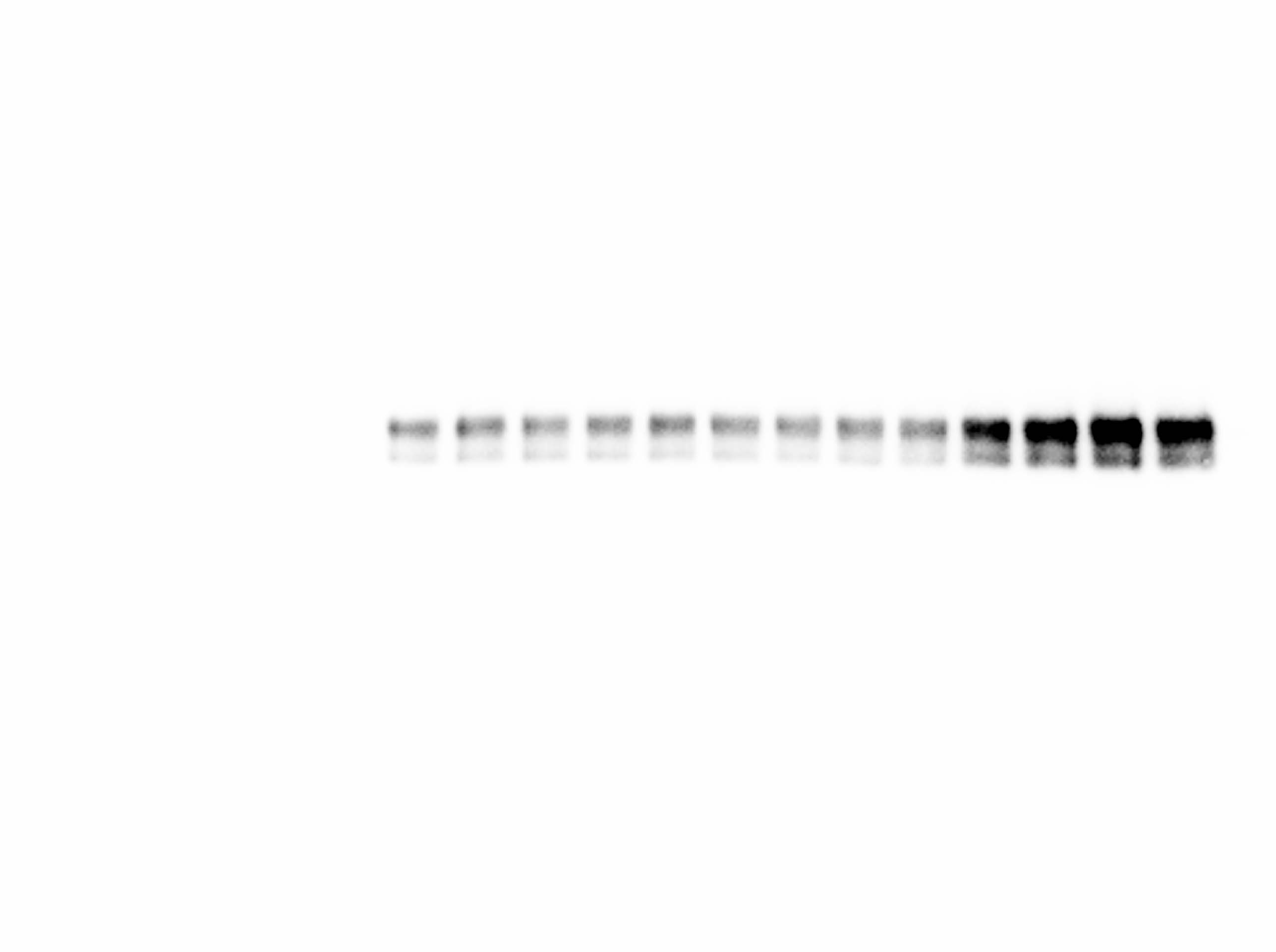

Supplement: Supplemental Information 16 — Raw data exported from western-blot for data analyses for Figs. 3C, 4 and Figs. S6–S8. [file peerj-07-7234-s016.zip › Western blot raw data figure 3C 4 s6 s7 s8/Figure 4 raw data/N4/N P653.jpg]

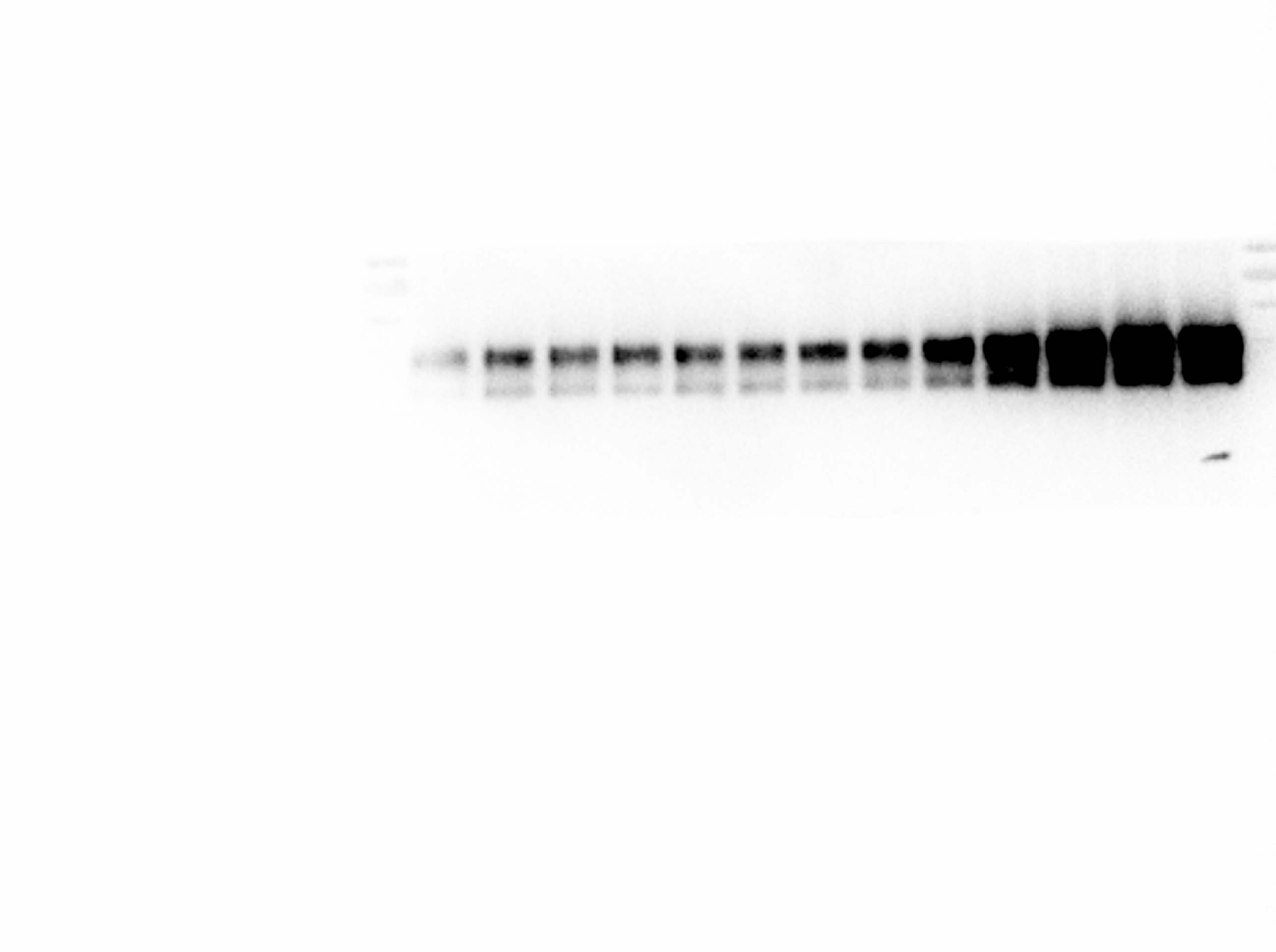

Supplement: Supplemental Information 16 — Raw data exported from western-blot for data analyses for Figs. 3C, 4 and Figs. S6–S8. [file peerj-07-7234-s016.zip › Western blot raw data figure 3C 4 s6 s7 s8/Figure 4 raw data/N4/N P654.jpg]

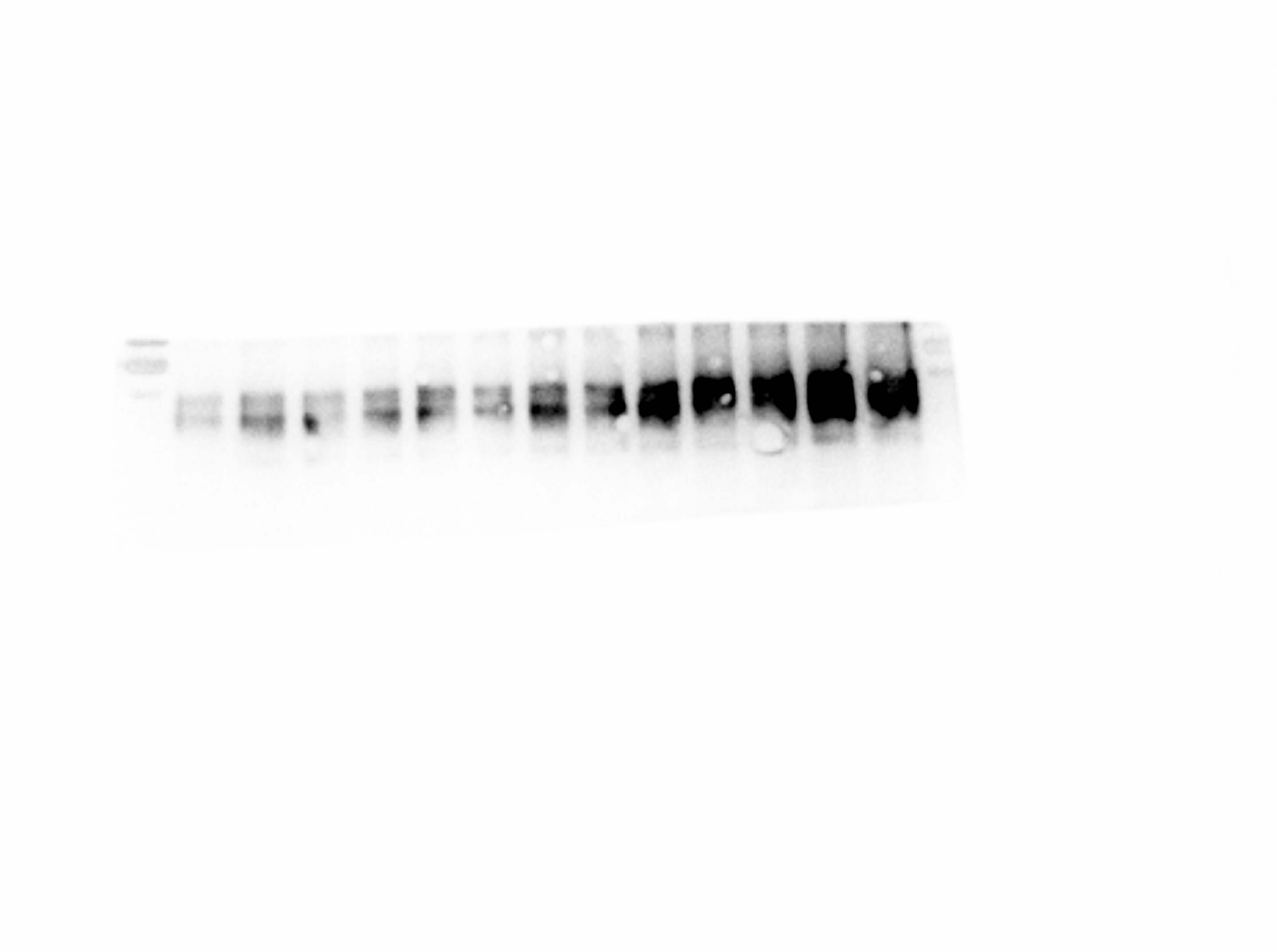

Supplement: Supplemental Information 16 — Raw data exported from western-blot for data analyses for Figs. 3C, 4 and Figs. S6–S8. [file peerj-07-7234-s016.zip › Western blot raw data figure 3C 4 s6 s7 s8/Figure 4 raw data/N4/N PY.jpg]

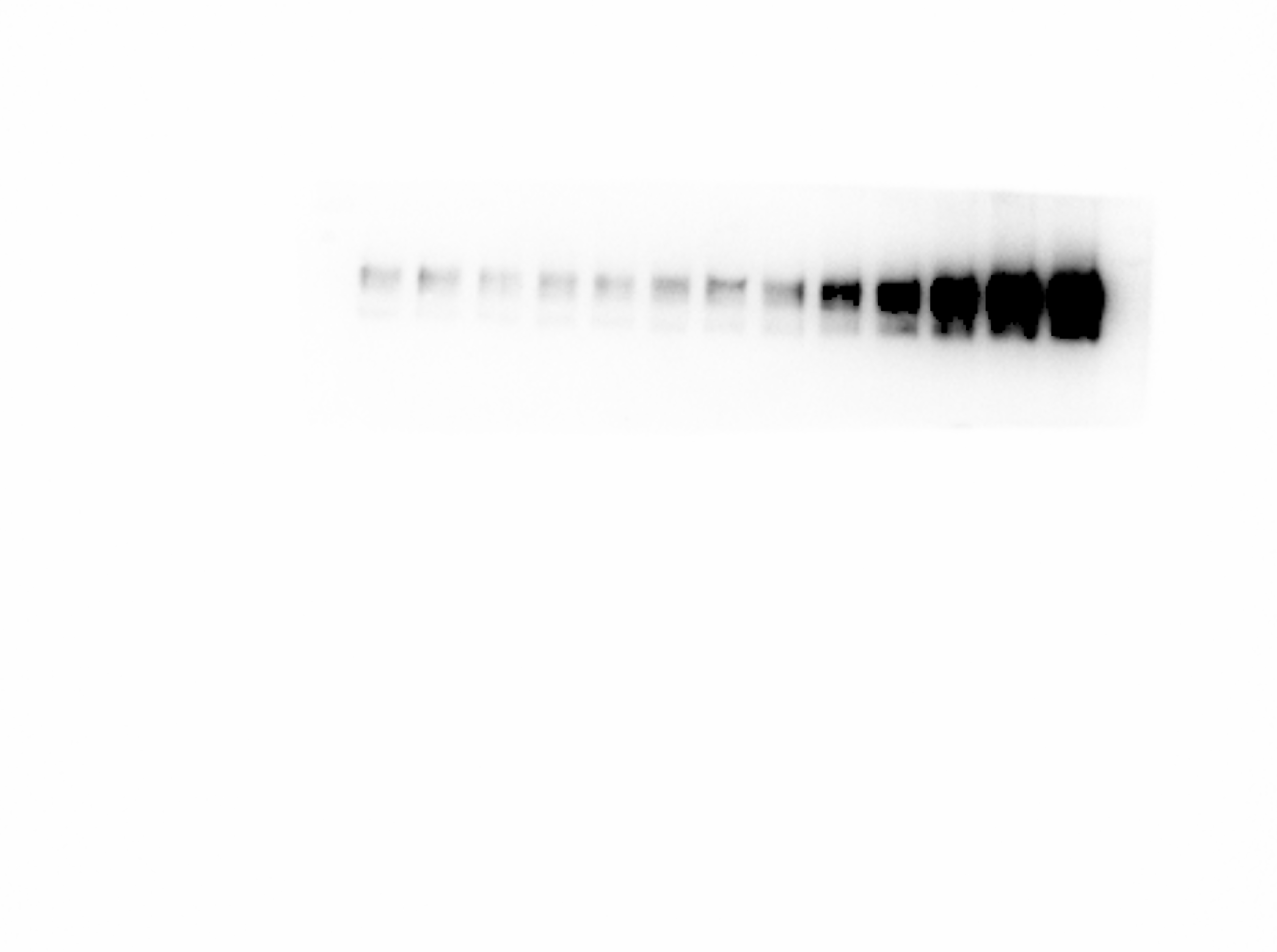

Supplement: Supplemental Information 16 — Raw data exported from western-blot for data analyses for Figs. 3C, 4 and Figs. S6–S8. [file peerj-07-7234-s016.zip › Western blot raw data figure 3C 4 s6 s7 s8/Figure 4 raw data/N5/N P654.jpg]

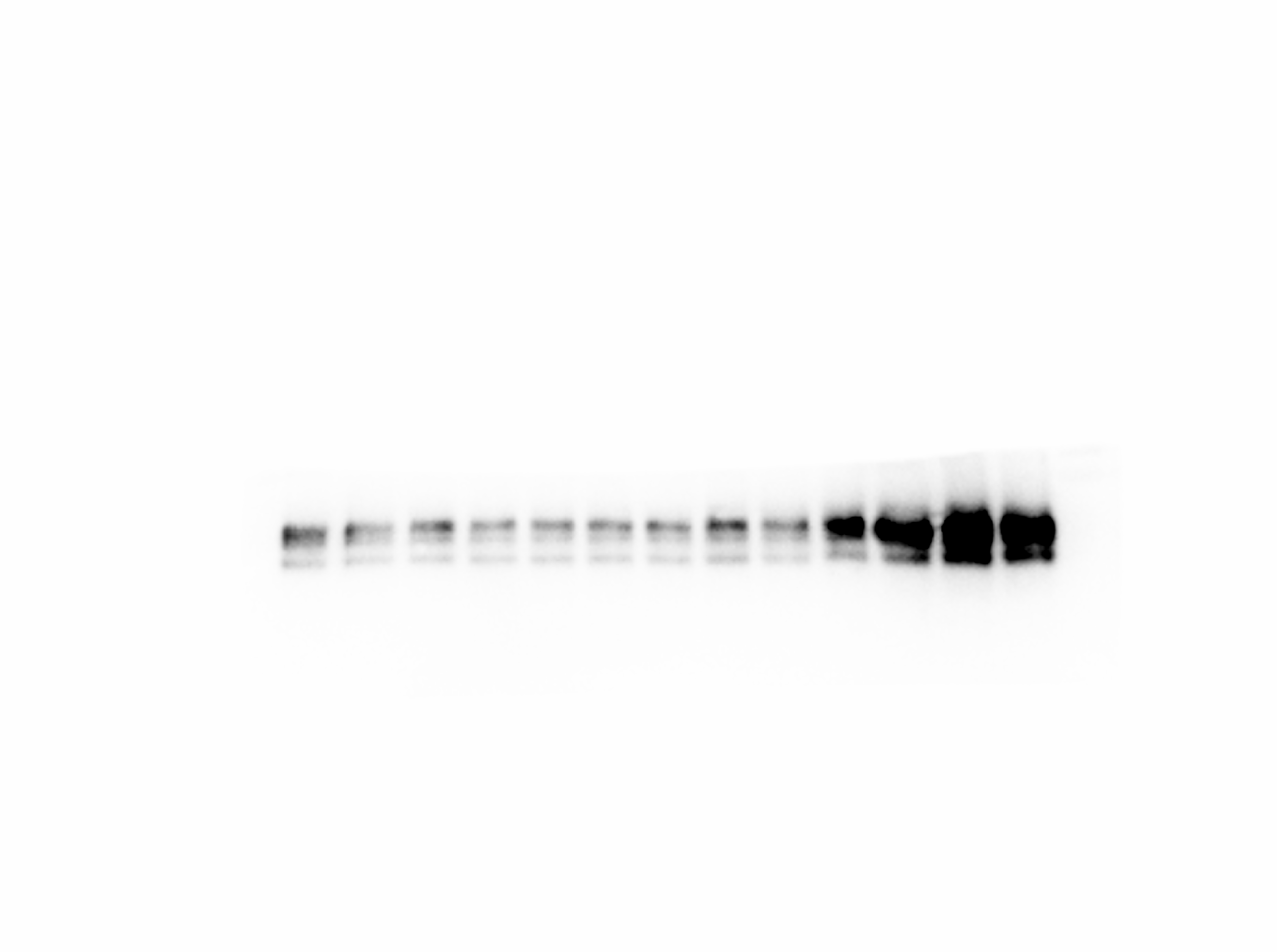

Supplement: Supplemental Information 16 — Raw data exported from western-blot for data analyses for Figs. 3C, 4 and Figs. S6–S8. [file peerj-07-7234-s016.zip › Western blot raw data figure 3C 4 s6 s7 s8/Figure 4 raw data/N5/N -P653.jpg]

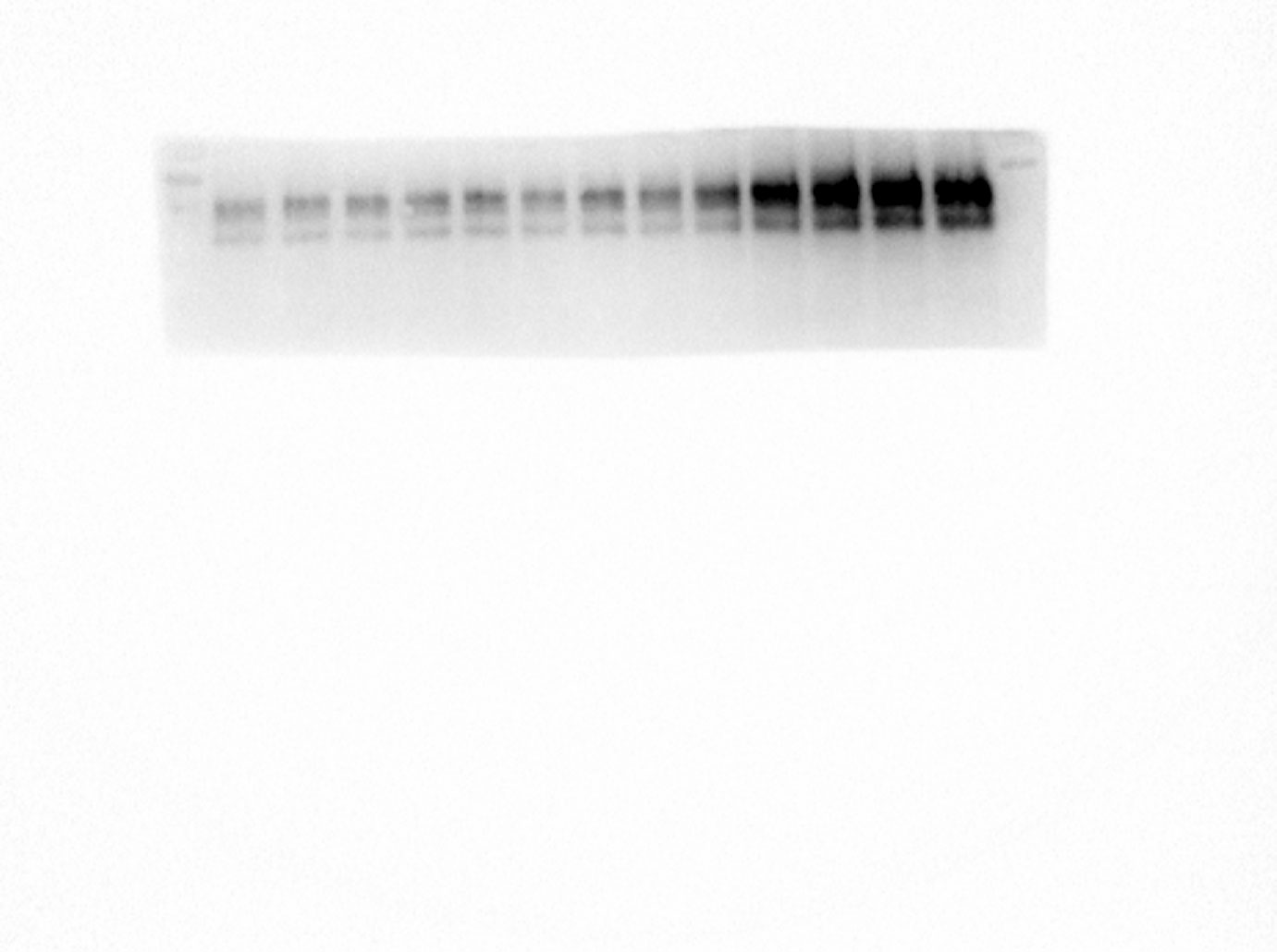

Supplement: Supplemental Information 16 — Raw data exported from western-blot for data analyses for Figs. 3C, 4 and Figs. S6–S8. [file peerj-07-7234-s016.zip › Western blot raw data figure 3C 4 s6 s7 s8/Figure 4 raw data/N5/N -PY.jpg]

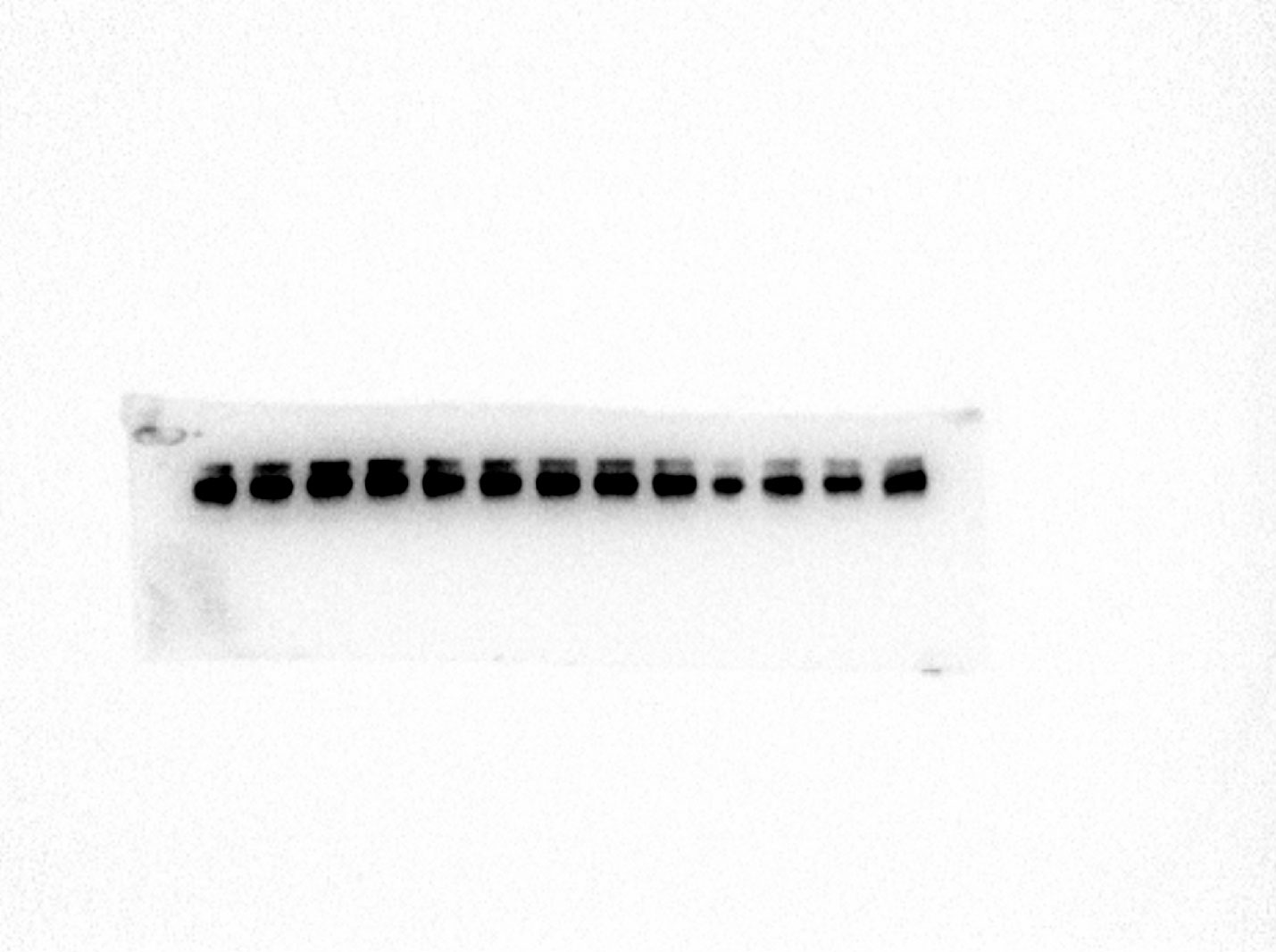

Supplement: Supplemental Information 16 — Raw data exported from western-blot for data analyses for Figs. 3C, 4 and Figs. S6–S8. [file peerj-07-7234-s016.zip › Western blot raw data figure 3C 4 s6 s7 s8/Figure 4 raw data/N5/N HIS.jpg]

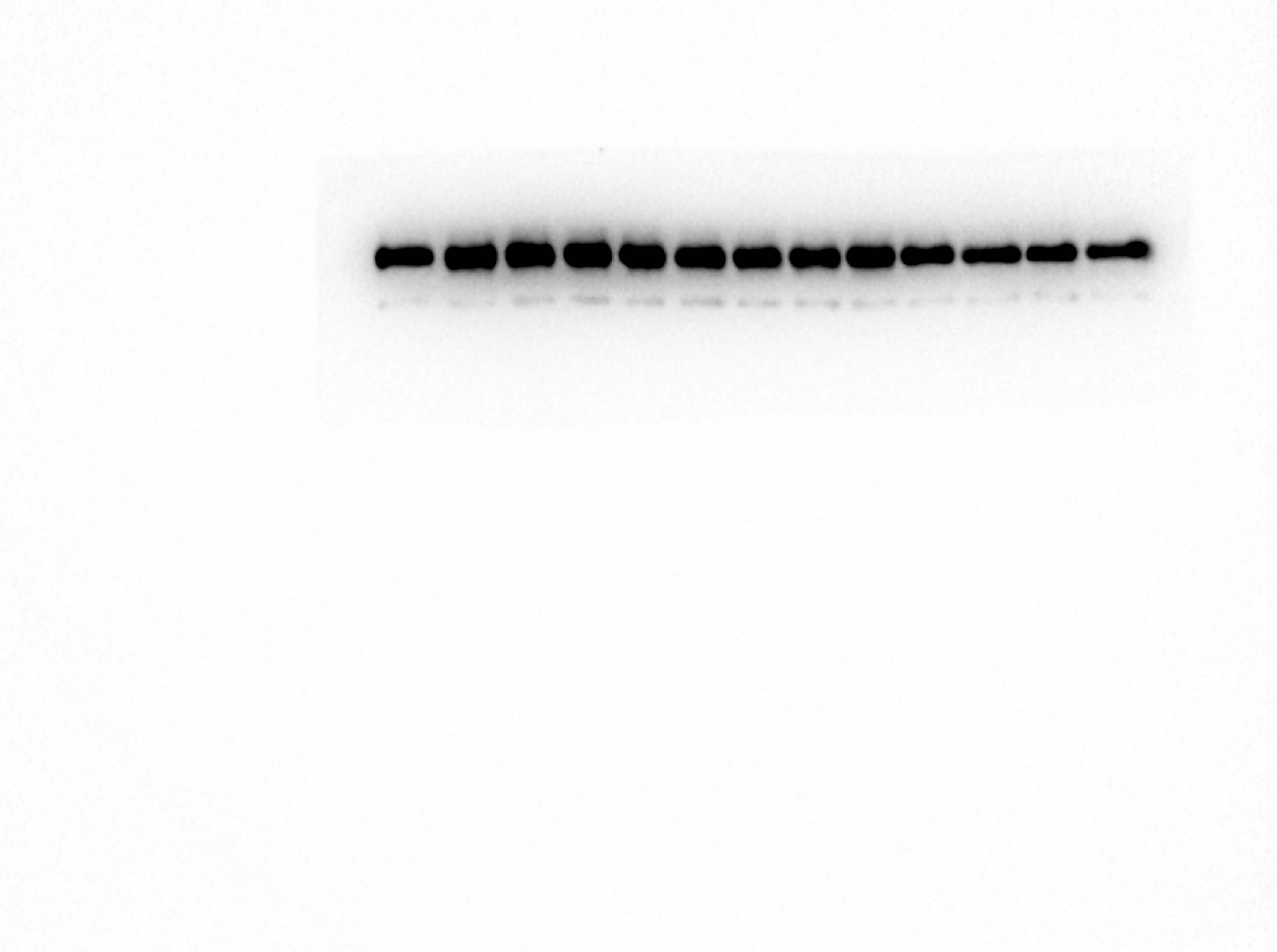

Supplement: Supplemental Information 16 — Raw data exported from western-blot for data analyses for Figs. 3C, 4 and Figs. S6–S8. [file peerj-07-7234-s016.zip › Western blot raw data figure 3C 4 s6 s7 s8/Figure 4 raw data/N6/N HIS.jpg]

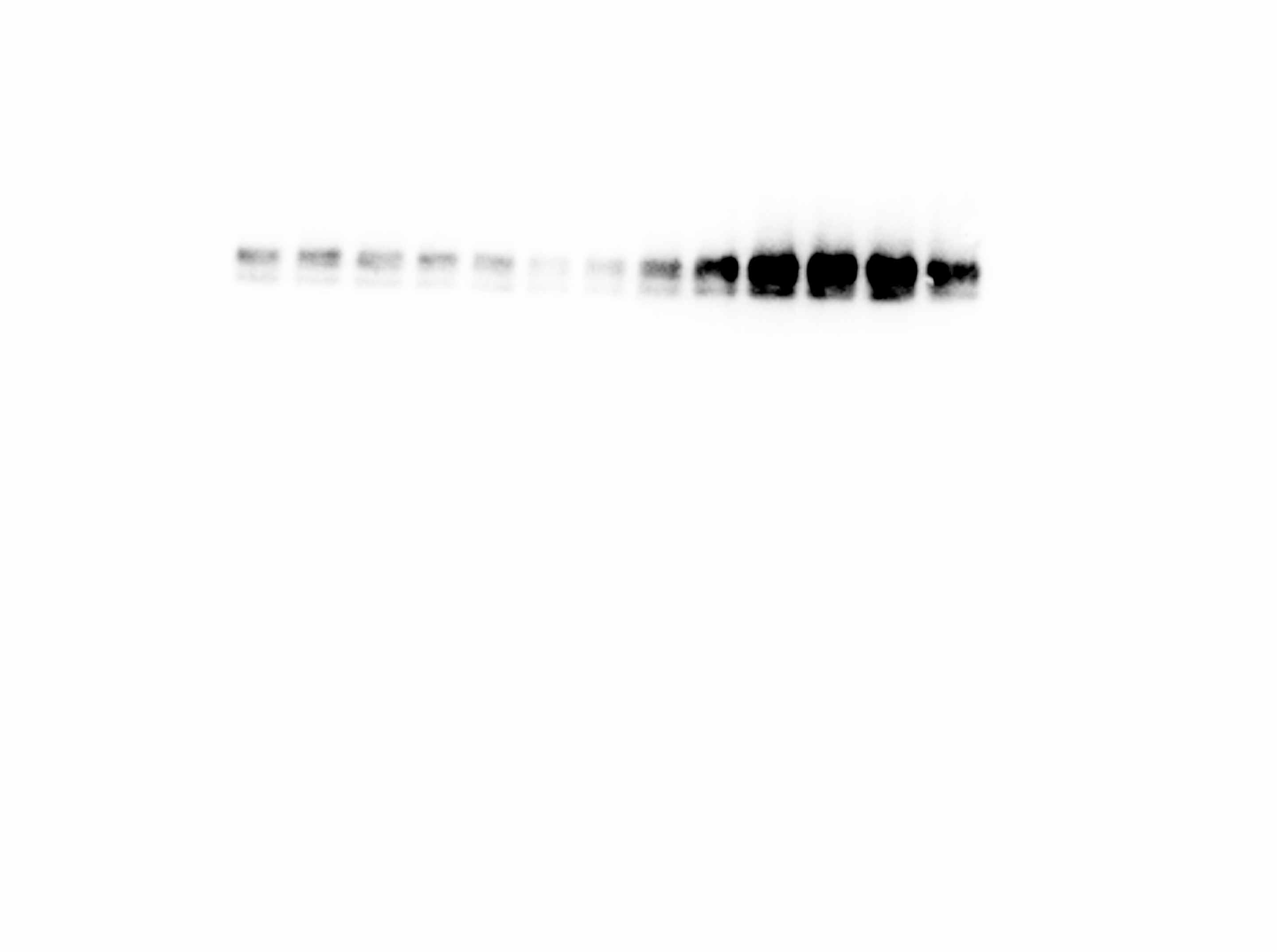

Supplement: Supplemental Information 16 — Raw data exported from western-blot for data analyses for Figs. 3C, 4 and Figs. S6–S8. [file peerj-07-7234-s016.zip › Western blot raw data figure 3C 4 s6 s7 s8/Figure 4 raw data/N6/N P-653.jpg]

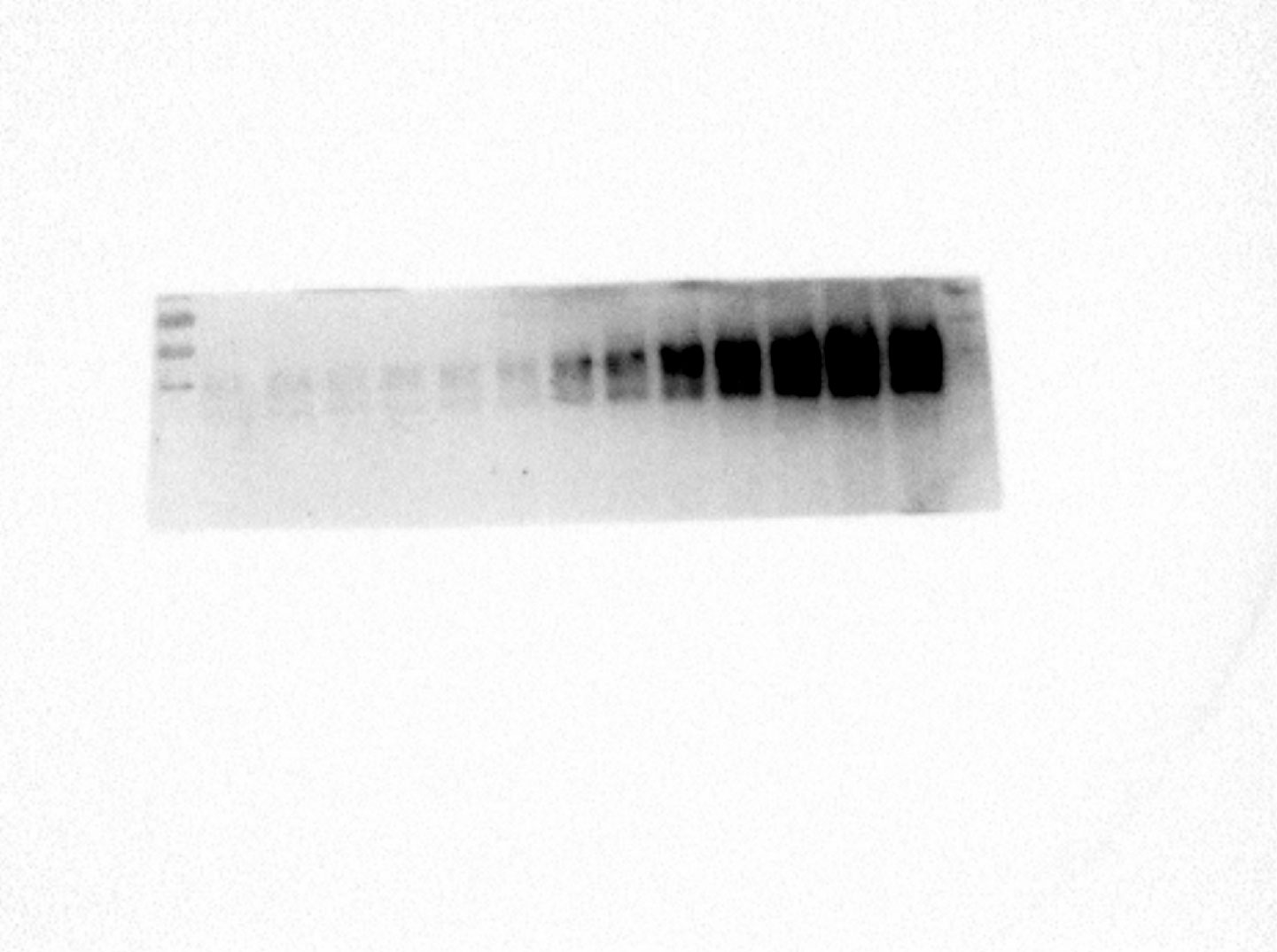

Supplement: Supplemental Information 16 — Raw data exported from western-blot for data analyses for Figs. 3C, 4 and Figs. S6–S8. [file peerj-07-7234-s016.zip › Western blot raw data figure 3C 4 s6 s7 s8/Figure 4 raw data/N6/N P-654.jpg]

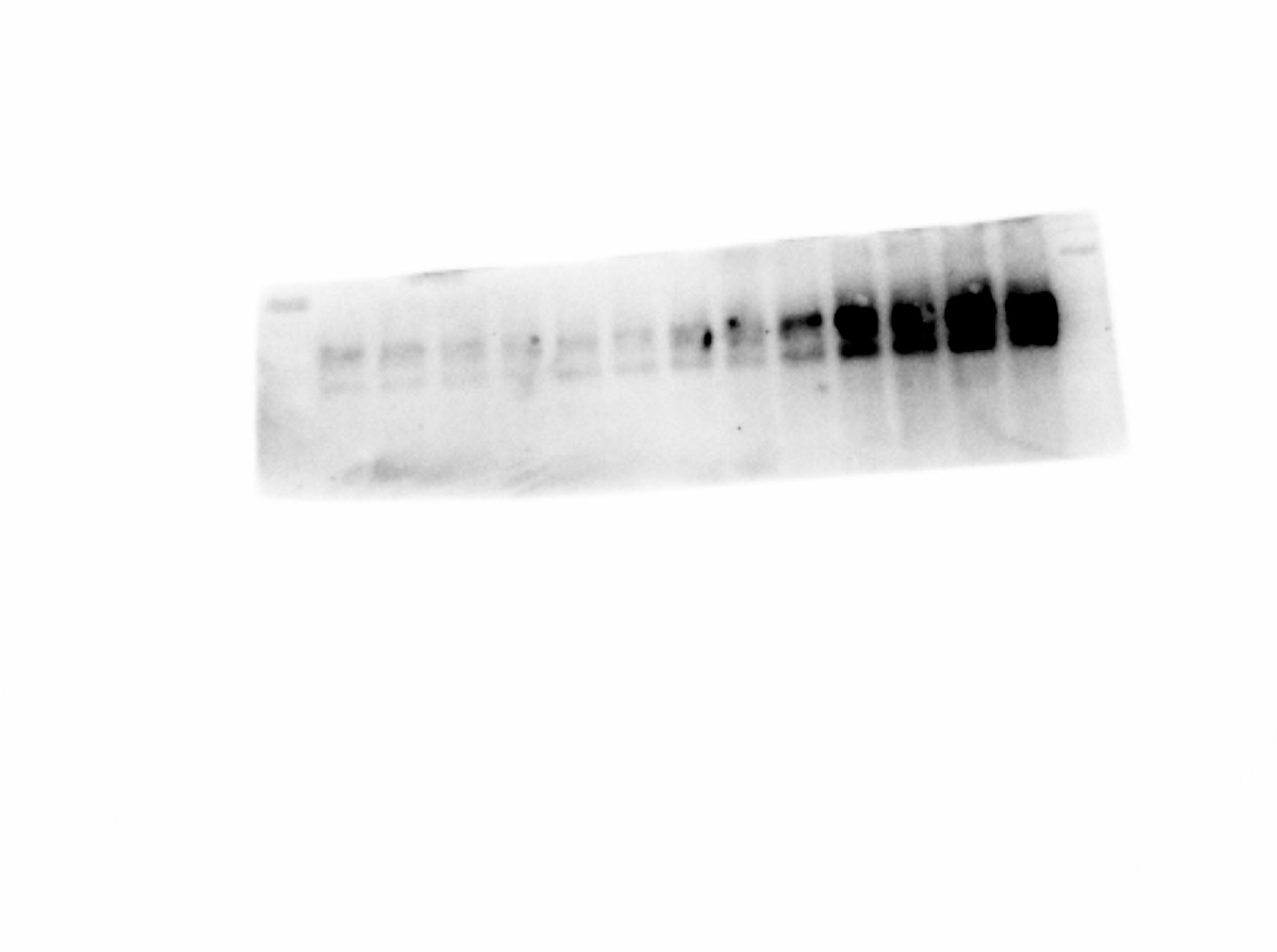

Supplement: Supplemental Information 16 — Raw data exported from western-blot for data analyses for Figs. 3C, 4 and Figs. S6–S8. [file peerj-07-7234-s016.zip › Western blot raw data figure 3C 4 s6 s7 s8/Figure 4 raw data/N6/N P-Y.jpg]

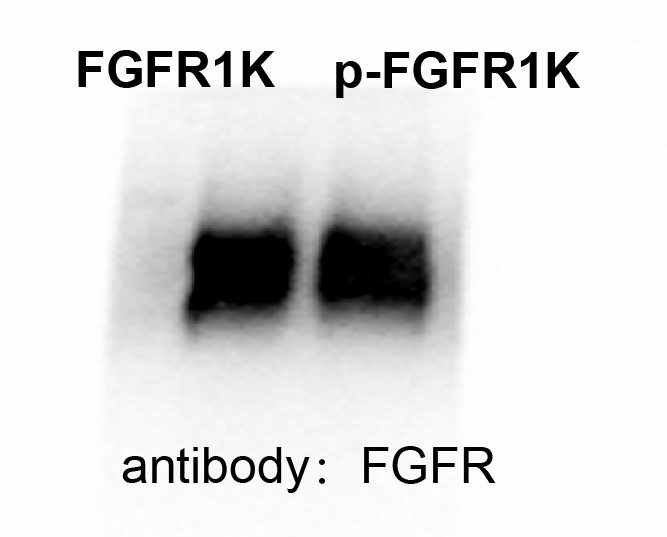

Supplement: Supplemental Information 16 — Raw data exported from western-blot for data analyses for Figs. 3C, 4 and Figs. S6–S8. [file peerj-07-7234-s016.zip › Western blot raw data figure 3C 4 s6 s7 s8/Figure S6 raw data/FGFR.png]

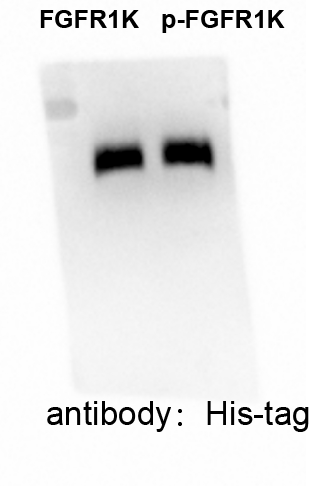

Supplement: Supplemental Information 16 — Raw data exported from western-blot for data analyses for Figs. 3C, 4 and Figs. S6–S8. [file peerj-07-7234-s016.zip › Western blot raw data figure 3C 4 s6 s7 s8/Figure S6 raw data/HIS.png]

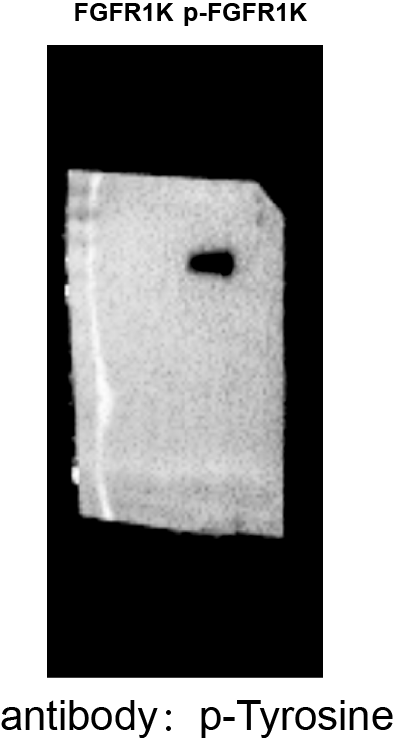

Supplement: Supplemental Information 16 — Raw data exported from western-blot for data analyses for Figs. 3C, 4 and Figs. S6–S8. [file peerj-07-7234-s016.zip › Western blot raw data figure 3C 4 s6 s7 s8/Figure S6 raw data/p-Tyrosine.png]

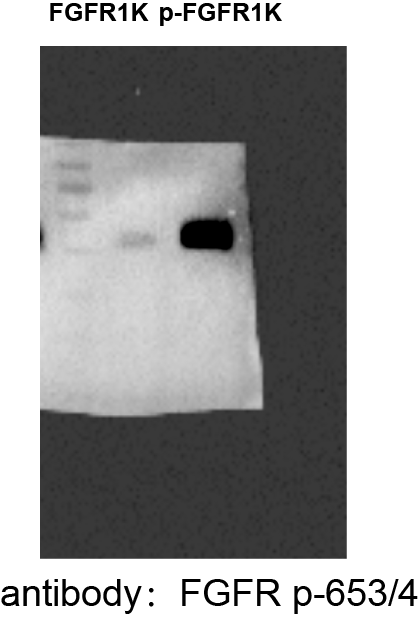

Supplement: Supplemental Information 16 — Raw data exported from western-blot for data analyses for Figs. 3C, 4 and Figs. S6–S8. [file peerj-07-7234-s016.zip › Western blot raw data figure 3C 4 s6 s7 s8/Figure S6 raw data/p-Y6534.png]

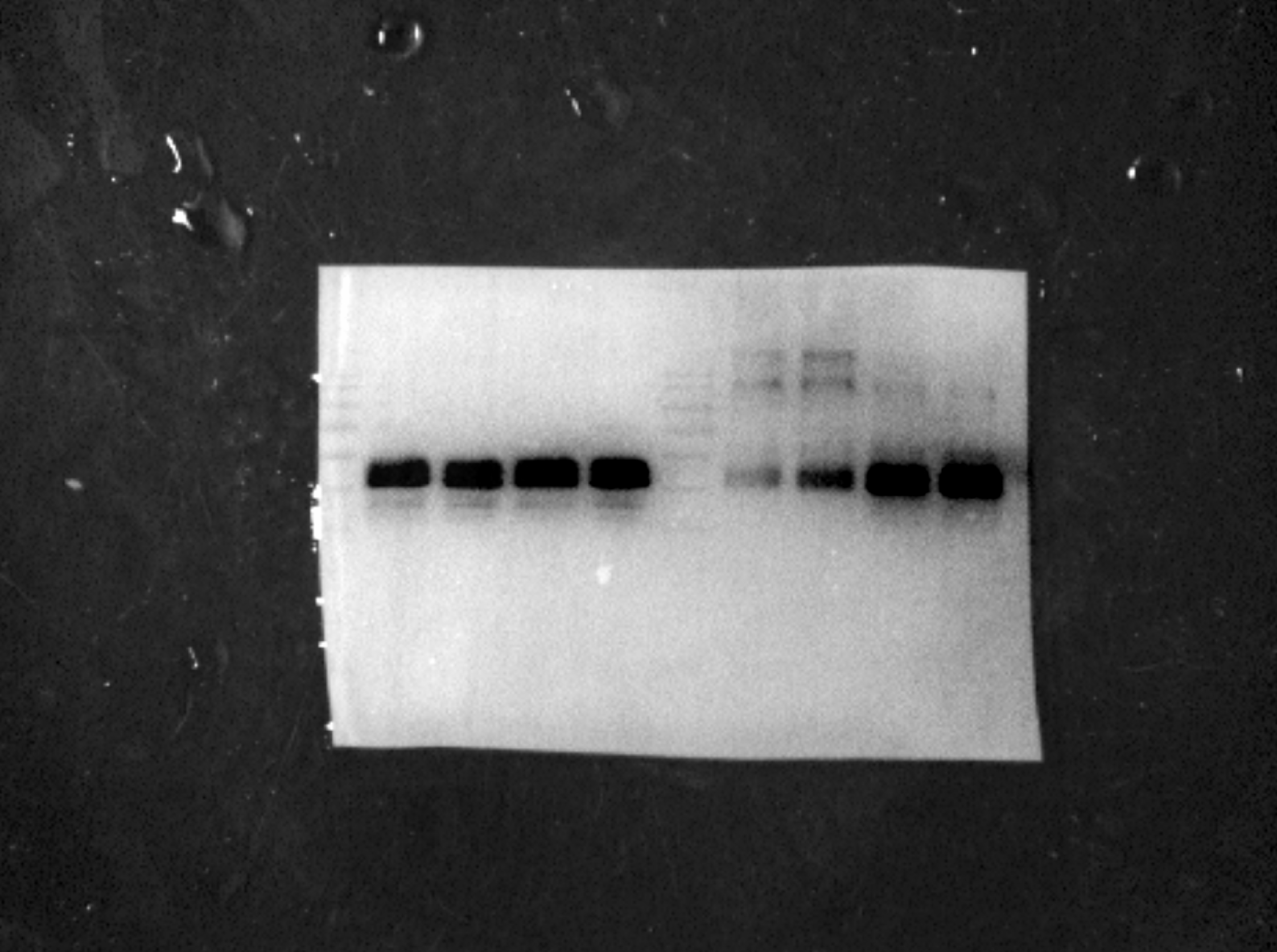

Supplement: Supplemental Information 16 — Raw data exported from western-blot for data analyses for Figs. 3C, 4 and Figs. S6–S8. [file peerj-07-7234-s016.zip › Western blot raw data figure 3C 4 s6 s7 s8/figure S7 raw data.jpg]

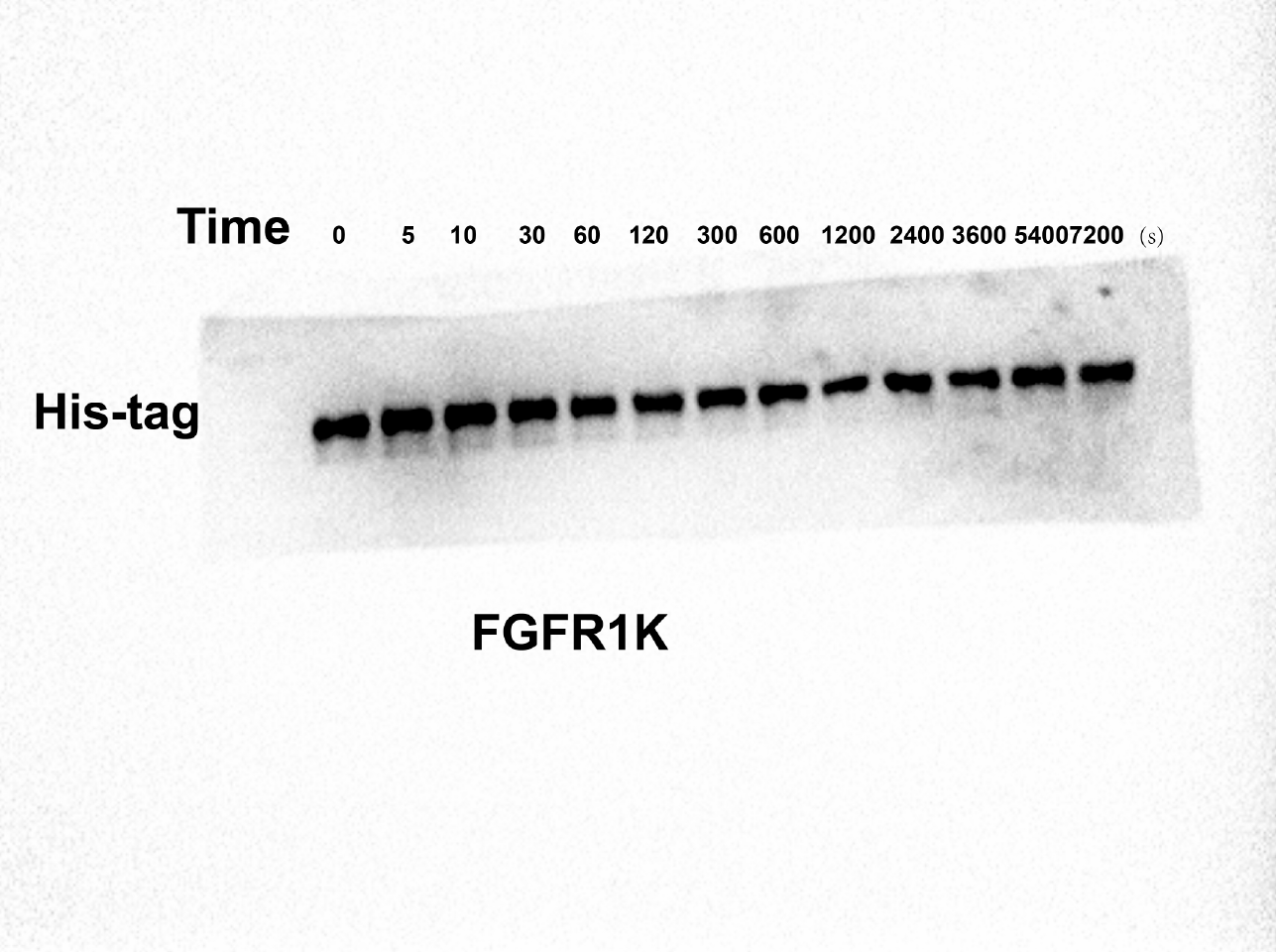

Supplement: Supplemental Information 16 — Raw data exported from western-blot for data analyses for Figs. 3C, 4 and Figs. S6–S8. [file peerj-07-7234-s016.zip › Western blot raw data figure 3C 4 s6 s7 s8/figure S8 raw data/C HIS.png]

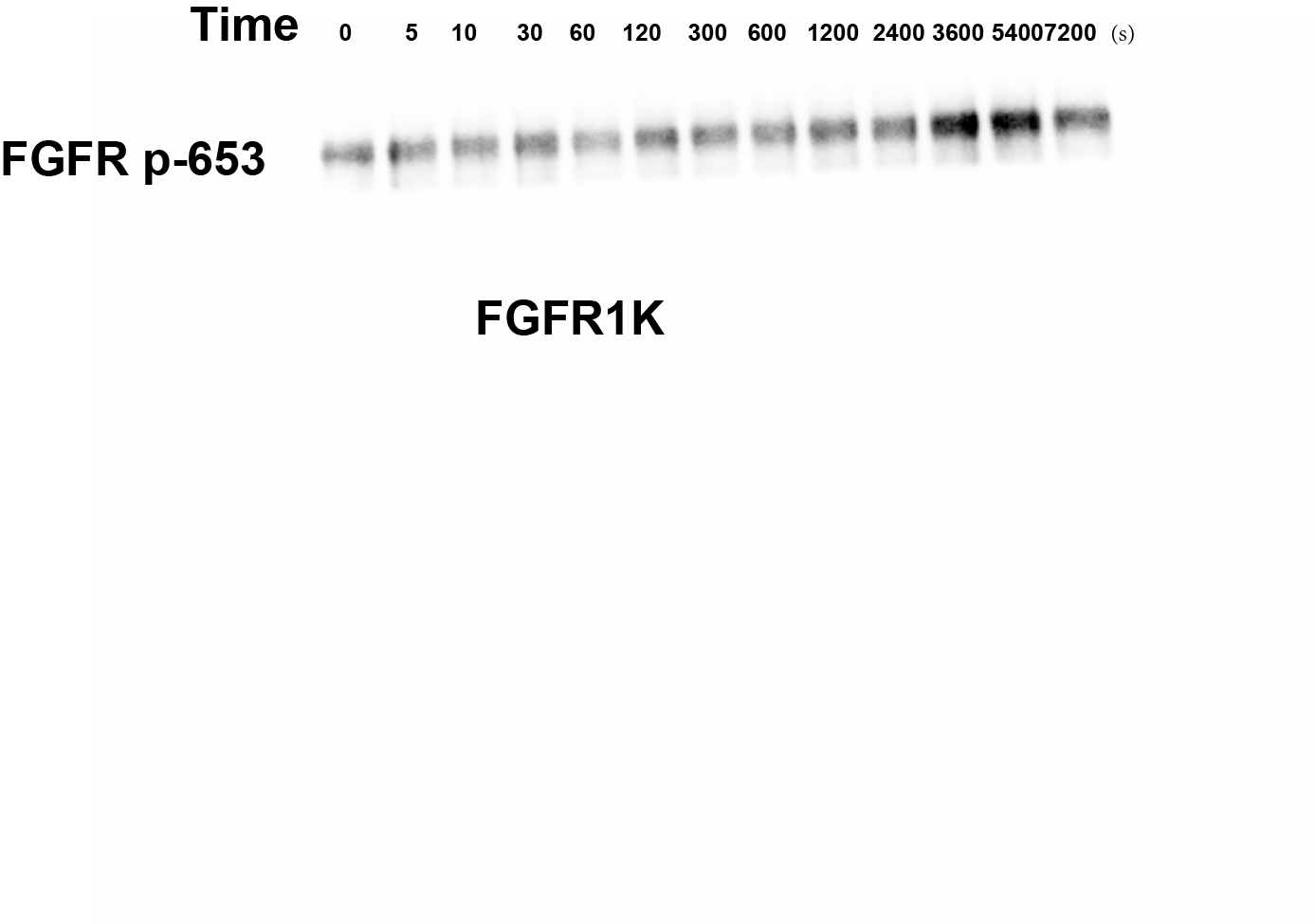

Supplement: Supplemental Information 16 — Raw data exported from western-blot for data analyses for Figs. 3C, 4 and Figs. S6–S8. [file peerj-07-7234-s016.zip › Western blot raw data figure 3C 4 s6 s7 s8/figure S8 raw data/C P653.png]

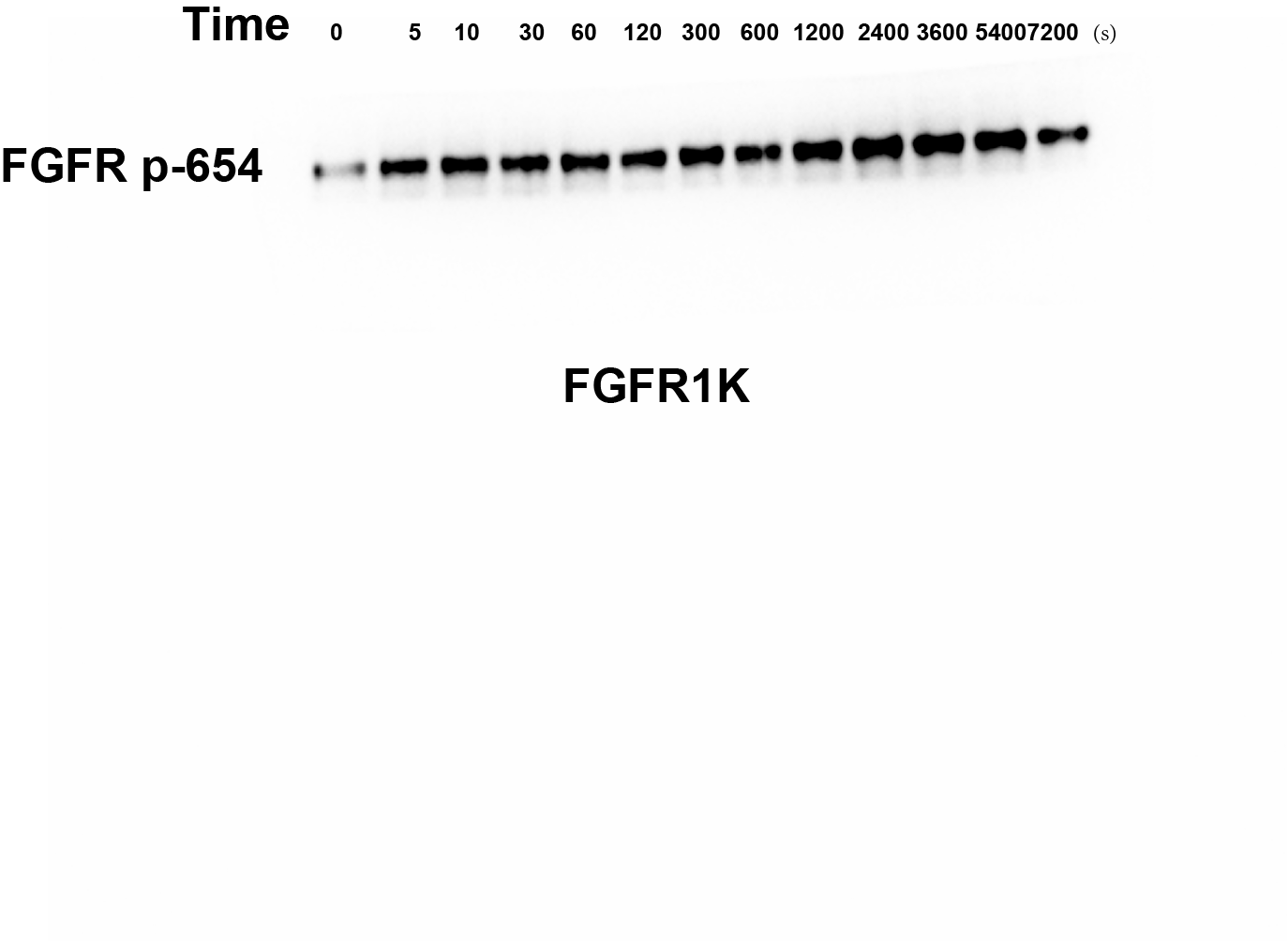

Supplement: Supplemental Information 16 — Raw data exported from western-blot for data analyses for Figs. 3C, 4 and Figs. S6–S8. [file peerj-07-7234-s016.zip › Western blot raw data figure 3C 4 s6 s7 s8/figure S8 raw data/C P654.png]

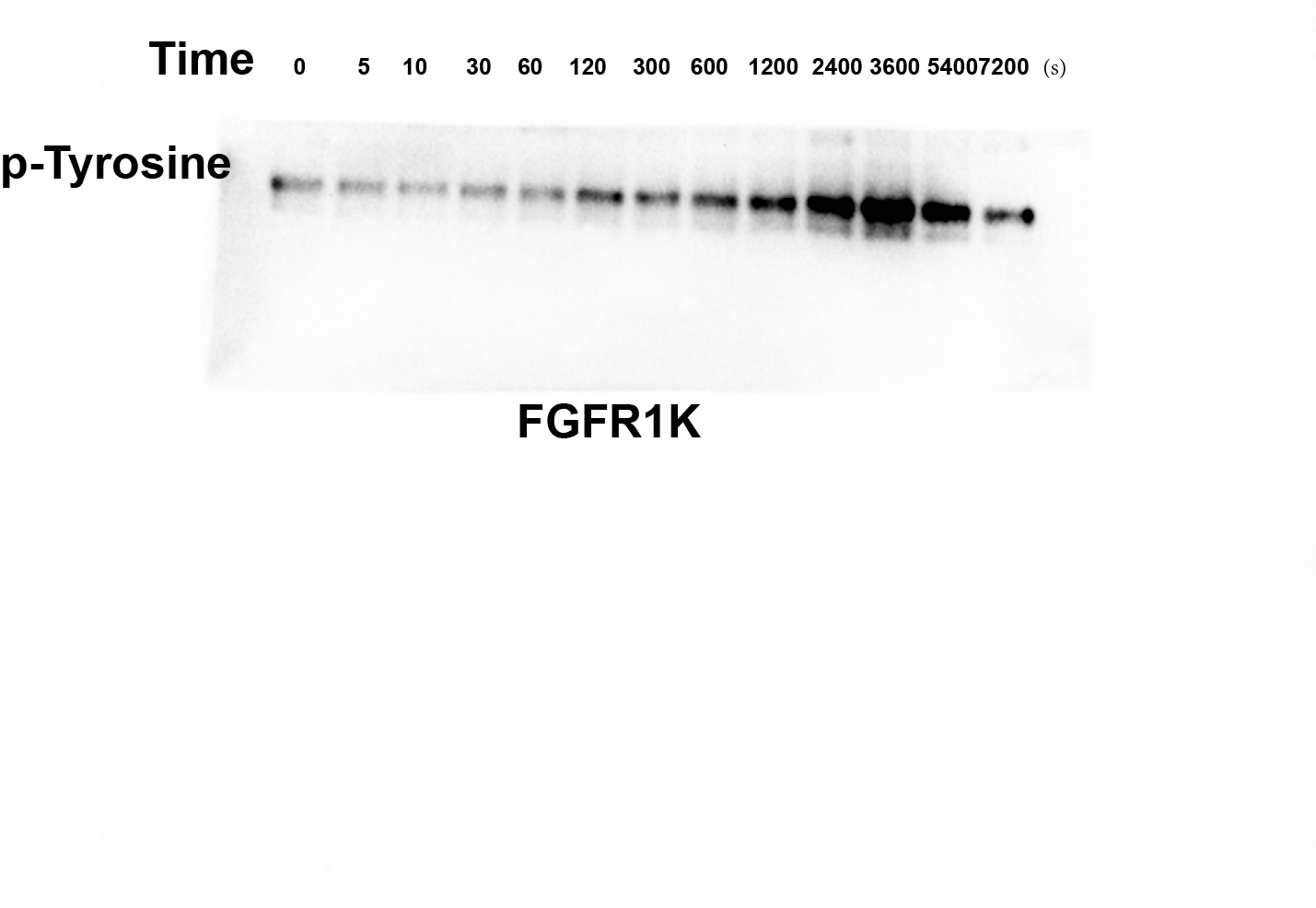

Supplement: Supplemental Information 16 — Raw data exported from western-blot for data analyses for Figs. 3C, 4 and Figs. S6–S8. [file peerj-07-7234-s016.zip › Western blot raw data figure 3C 4 s6 s7 s8/figure S8 raw data/C p-Tyrosine.png]

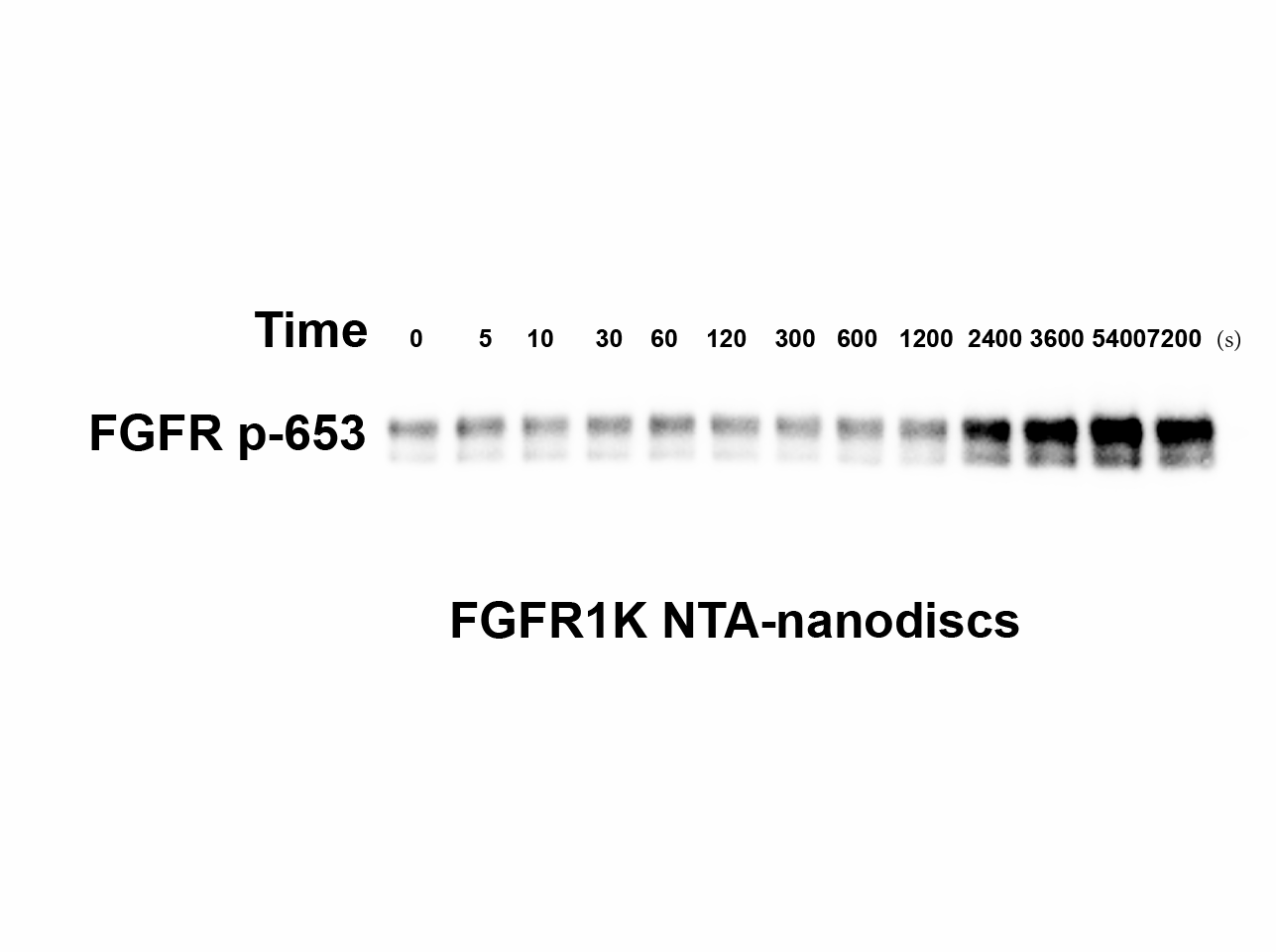

Supplement: Supplemental Information 16 — Raw data exported from western-blot for data analyses for Figs. 3C, 4 and Figs. S6–S8. [file peerj-07-7234-s016.zip › Western blot raw data figure 3C 4 s6 s7 s8/figure S8 raw data/N P653.png]

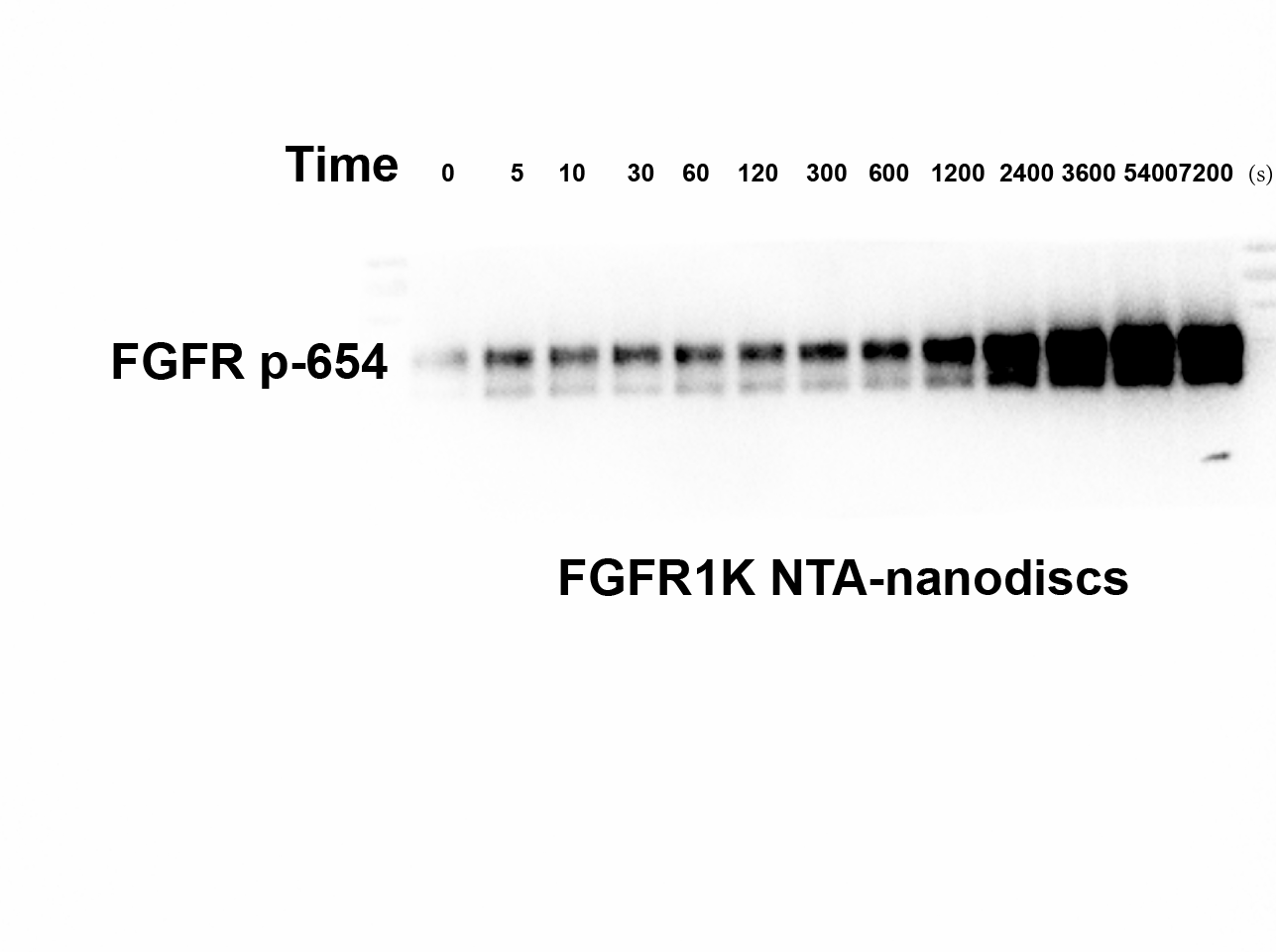

Supplement: Supplemental Information 16 — Raw data exported from western-blot for data analyses for Figs. 3C, 4 and Figs. S6–S8. [file peerj-07-7234-s016.zip › Western blot raw data figure 3C 4 s6 s7 s8/figure S8 raw data/N P654.png]

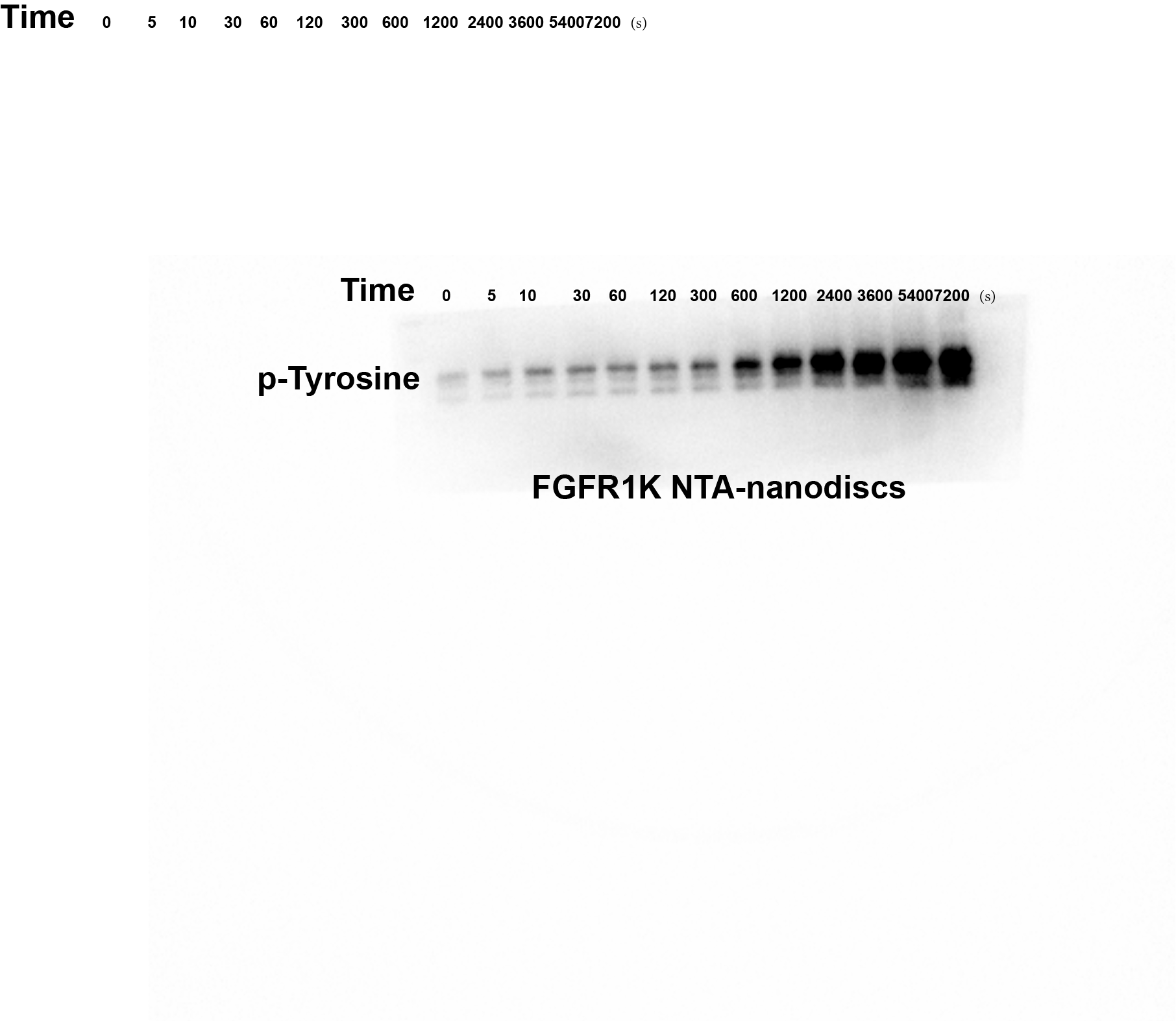

Supplement: Supplemental Information 16 — Raw data exported from western-blot for data analyses for Figs. 3C, 4 and Figs. S6–S8. [file peerj-07-7234-s016.zip › Western blot raw data figure 3C 4 s6 s7 s8/figure S8 raw data/N p-Tyrosine.png]

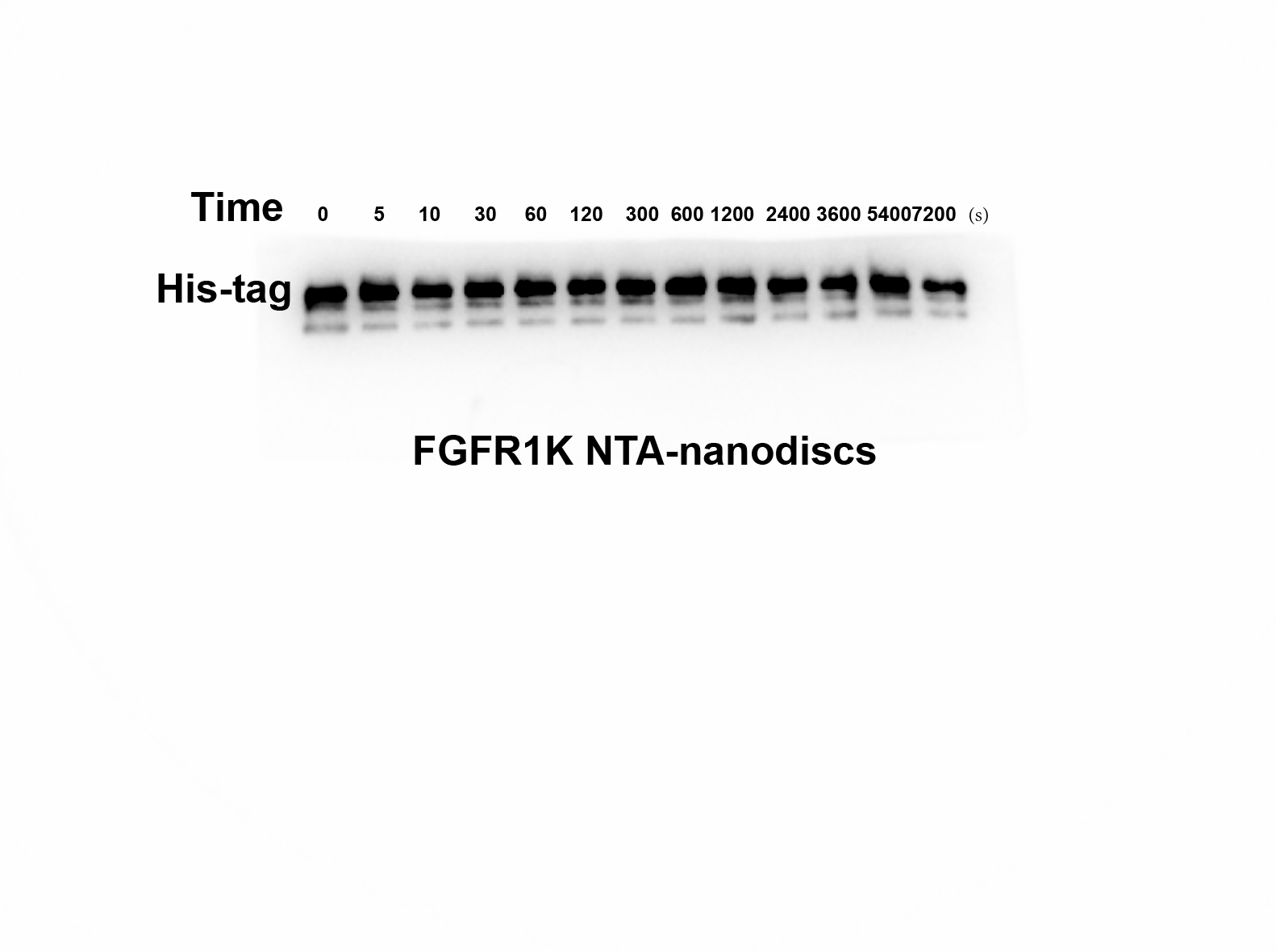

Supplement: Supplemental Information 16 — Raw data exported from western-blot for data analyses for Figs. 3C, 4 and Figs. S6–S8. [file peerj-07-7234-s016.zip › Western blot raw data figure 3C 4 s6 s7 s8/figure S8 raw data/N-HIS.png]
