# Supplementary material for: Peptide-based NTA(Ni)-nanodiscs for studying membrane enhanced FGFR1 kinase activities
Source: PeerJ. 2019 Jul 23;7:e7234. doi: 10.7717/peerj.7234 (PMC6659669; doi:10.7717/peerj.7234)
Supplement: Supplemental Information 9 — The particle size measurement from TEM images were made on the 2D class average analysis. PDI (polydispersity Index) represents the monodisperse standards from Zetasizer software. Values smaller than 0.7 indicate the sample has a reasonably narrow size distribution. [file peerj-07-7234-s009.pdf]

Table S1:

Peptide nanodisc size distribution by TEM and DLS

The particle size measurement from TEM images were made on the 2D class average analysis. PDI (polydispersity Index) represents the monodisperse standards from Zetasizer software. Values smaller than 0.7 indicate the sample has a reasonably narrow size distribution.

**Lipid:peptide (molar)**	**TEM(diameter)**	**DLS (diameter, PDI)**
**1:1**	**12.0 ± 1.8 nm**	**11.5±2.7 nm, 0.25**
**1:3**	**8.6±1.2 nm**	**7.2±1.5 nm, 0.65**
**1:9**	**6.3±1.1 nm**	**5.1±1.2 nm, 0.33**
