# Supplementary material for: Peptide-based NTA(Ni)-nanodiscs for studying membrane enhanced FGFR1 kinase activities
Source: PeerJ. 2019 Jul 23;7:e7234. doi: 10.7717/peerj.7234 (PMC6659669; doi:10.7717/peerj.7234)
Supplement: Supplemental Information 10 [file peerj-07-7234-s010.docx]

**Supplementary Materials**

**Peptide-based NTA(Ni)-nanodiscs for studying membrane enhanced FGFR1 kinase activities**

Juanjuan Liu^1, 2^, Lei Zhu^1^, Xueli Zhang^3,4^, Bo Wu^1^, Ping Zhu^3,4^, Hongxin Zhao^1^ and Junfeng Wang^1,2,5^

^1^ High Magnetic Field Laboratory, Key Laboratory of High Magnetic Field and Ion Beam Physical Biology, Hefei Institutes of Physical Science, Chinese Academy of Sciences, Hefei 230031, Anhui, P. R. China.

^2^ University of Science and Technology of China, Hefei 230036, Anhui, P. R. China.

^3^ National Laboratory of Biomacromolecules, CAS Center for Excellence in Biomacromolecules, Institute of Biophysics, Chinese Academy of Sciences, Beijing 100101, China.

^4^ University of Chinese Academy of Sciences, Beijing 100049, China.

^5^ Institute of Physical Science and Information Technology, Anhui University, Hefei 230031, Anhui, P. R. China.

Corresponding Author:

Hongxin Zhao^1^

Shushanhu road, Hefei, Anhui, 230031, China

Email address: [zhx@hmfl.ac.cn](mailto:zhx@hmfl.ac.cn)

Junfeng Wang^1,2,5^

Shushanhu road, Hefei, Anhui, 230031, China

Email address: [junfeng@hmfl.ac.cn](mailto:junfeng@hmfl.ac.cn)

**Materials and Methods**

**Preparation of the** **MSPΔH5-nanodiscs**

MSPΔH5, a truncated MSP1D1 protein variant lacking helix 5 (residues 121-142), was cloned into the pET28a vector with an N-terminal His_6_ tag and a TEV protease site. The recombinant protein was expressed in E.coli BL21(DE3) cells induced with 0.5 mM IPTG at 25 ℃ for 4 h, and then purified using Ni-NTA affinity chromatography. Nanodiscs were prepared as described previously([Denisov et al. 2004](#_ENREF_1)). Briefly, cholate-solubilized lipid and MSP protein were incubated at a molar ratio of 50:1. Nanodiscs formed after the removal of cholate by Bio-beads, and then were loaded on a Superdex 200 GL 10/300 column (GE Healthcare) at a flow rate of 0.5ml/min at room temperature 20 mM Tris, pH7.2, 100 mM NaCl as the running buffer to further purify ([Hagn et al. 2013](#_ENREF_2)).

**Dynamic Light Scattering (DLS)**

The size and size distribution of the nanodiscs were measured at 25°C using DLS with a Malvern Zetasizer nano ZS (Malvern, UK) instrument. The refractive index was assumed to be equal to that of water. Nanodisc samples were diluted to 40 nM. All samples were analyzed in triplicate. The size distributions of nanodiscs were generated through analyses of autocorrelation functions using Zetasizer software.

**Cross-linking Assays**

For cross-linking assays, FGFR1K and FGFR1K NTA(Ni)-nanodiscs were dialyzed into a buffer containing 0.1 M phosphate and 0.15 M NaCl (pH 7.2). Reactions were initiated by adding a 50-fold molar excess of crosslinker to the protein for 30 min at room temperature and then terminated by adding 50 mM Tris. Western blotting analyses were performed after adding 6× loading buffer without dithiothreitol (DTT) to each sample.


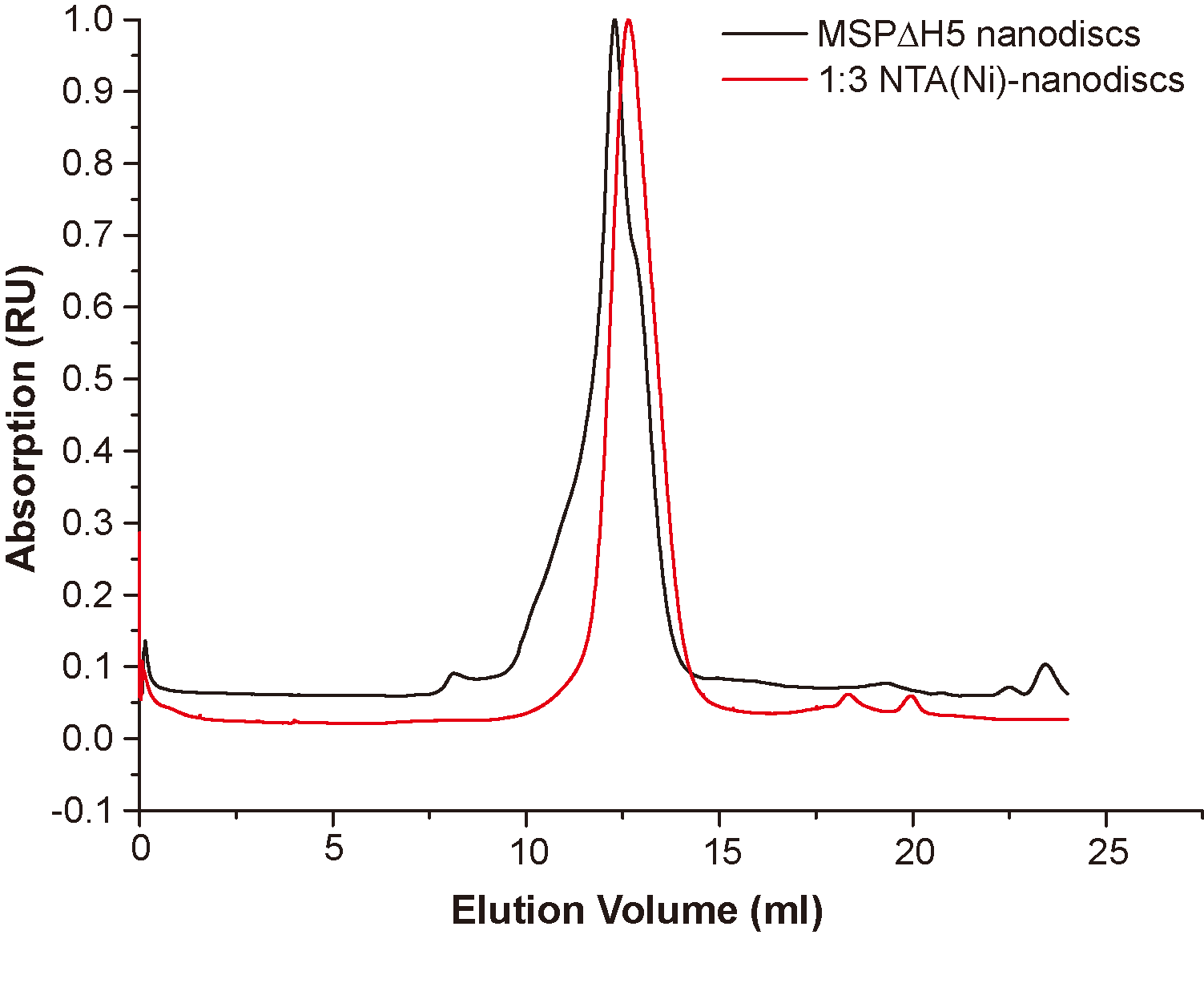


Figure S1 Size exclusion chromatography with Superdex 200 10/300 column of peptide NTA(Ni)-nanodisc at a lipid:peptide ratio of 1:3 (red) and that of MSPΔH5-nanodiscs (black).


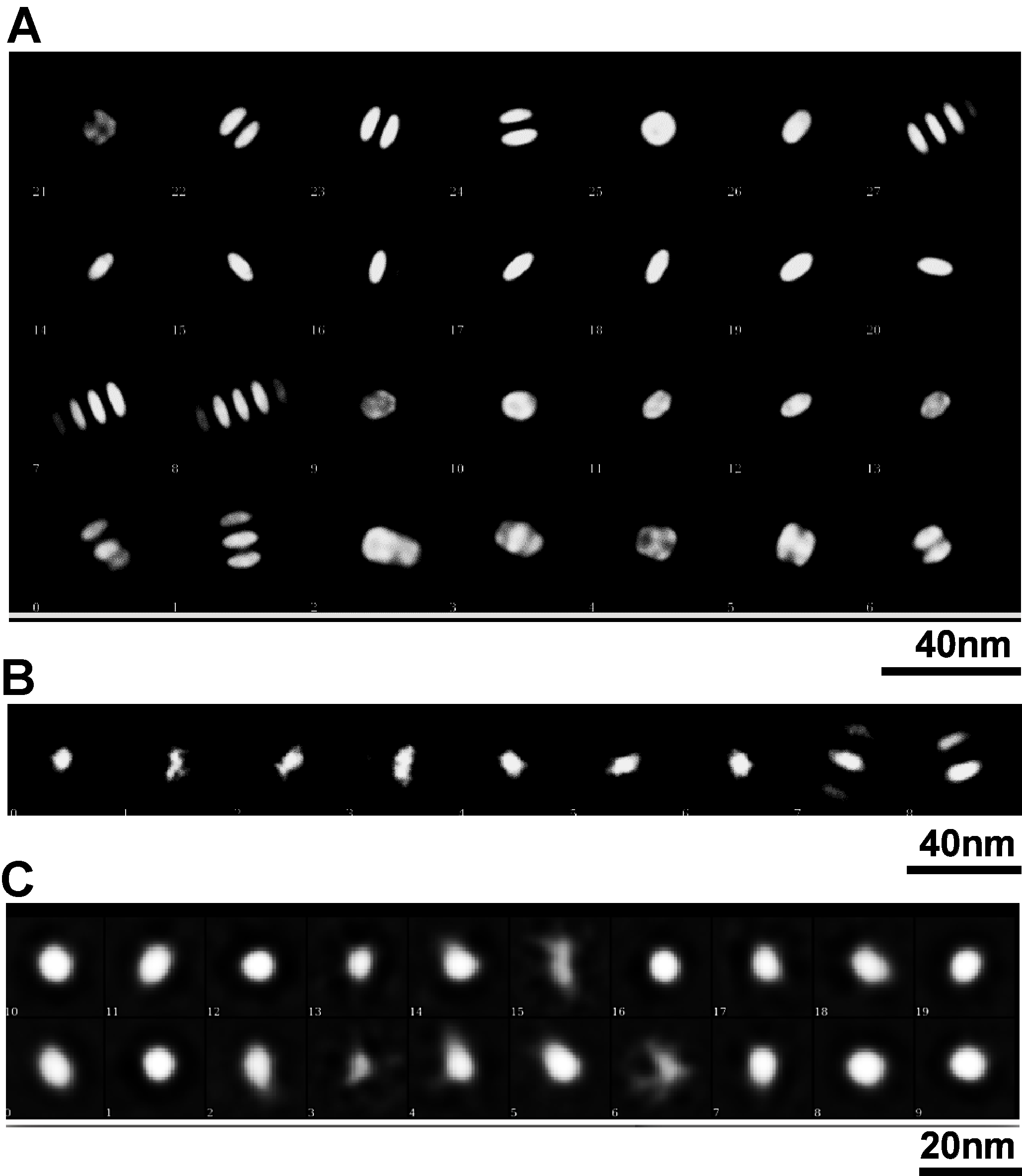


Figure S2 Two-dimensional classification of peptide nanodiscs of molar ratios of 1:1 (A), 1:3 (B), and 1:9 (C).


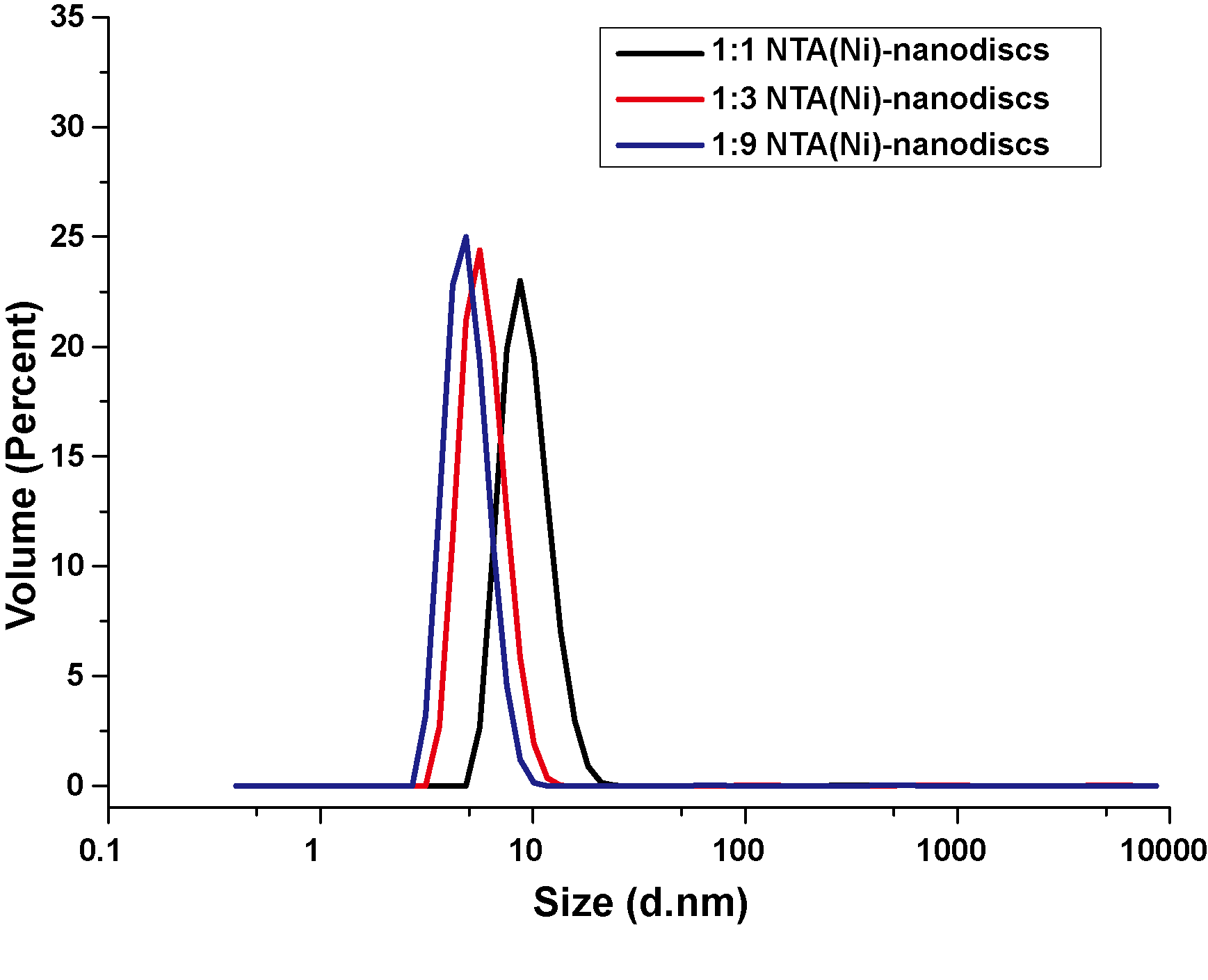


Figure S3 Dynamic Light Scattering measurement of peptide NTA(Ni)-nanodiscs at lipid:peptide molar ratios of 1:1 (black), 1:3(red) and 1:9(blue).

Table S1:

**Peptide nanodisc size distribution by TEM and DLS.**

The particle size measurement from TEM images were made on the 2D class average analysis. PDI (polydispersity Index) represents the monodisperse standards from Zetasizer software. Values smaller than 0.7 indicate the sample has a reasonably narrow size distribution.

| Lipid:peptide (molar) | TEM(diameter) | DLS (diameter, PDI) | |
| --- | --- | --- | --- |
| 1:1 | **12.0 ± 1.8 nm** | | **11.****5 ± 2.7 nm, 0.25** |
| 1:3 | **8.6 ± 1.2** **nm** | | **7.2 ± 1.5 nm, 0.65** |
| 1:9 | **6.3 ± 1.1 nm** | | **5.1 ± 1.2 nm, 0.33** |


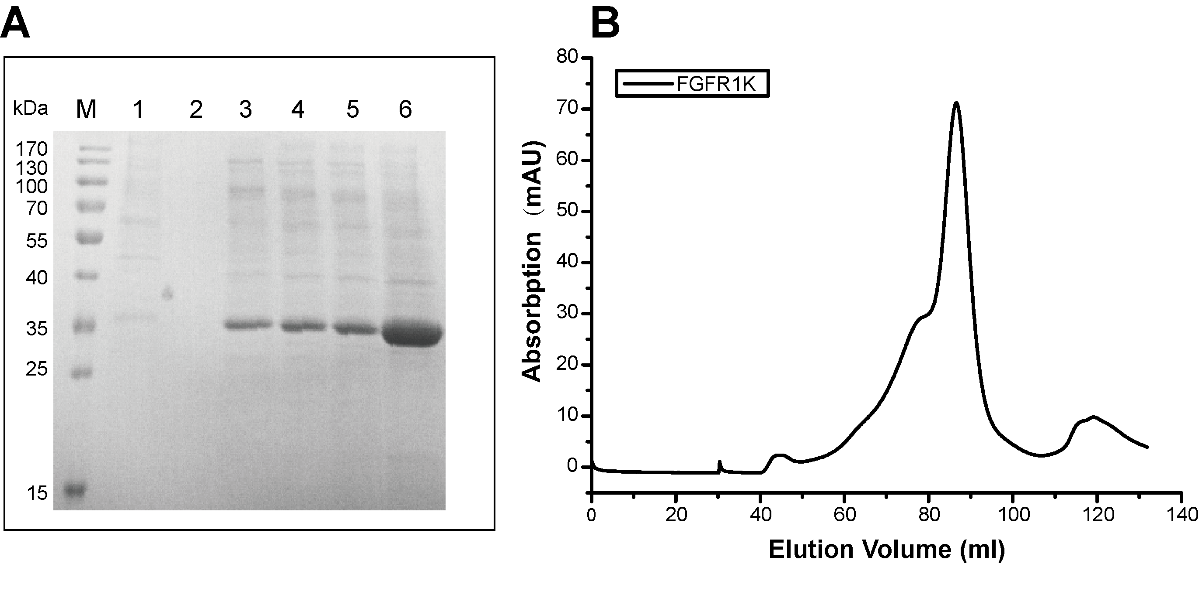


Figure S4 Recombinant expression and purification of FGFR1K. (A) SDS PAGE gel of elution aliquots from Ni-NTA affinity purification of FGFR1K protein. M: protein marks with molecular weights as labeled; 1: flow through; 2: wash buffer elution; 3, 4, 5: elutions with 30 mM imidazole; 6: elution with 300 mM imidazole. (B) Size exclusion chromatography of FGFR1K (Ni-NTA elution with 300mM imidazole) with a Superdex 200 GL 16/60 column (GE Healthcare).


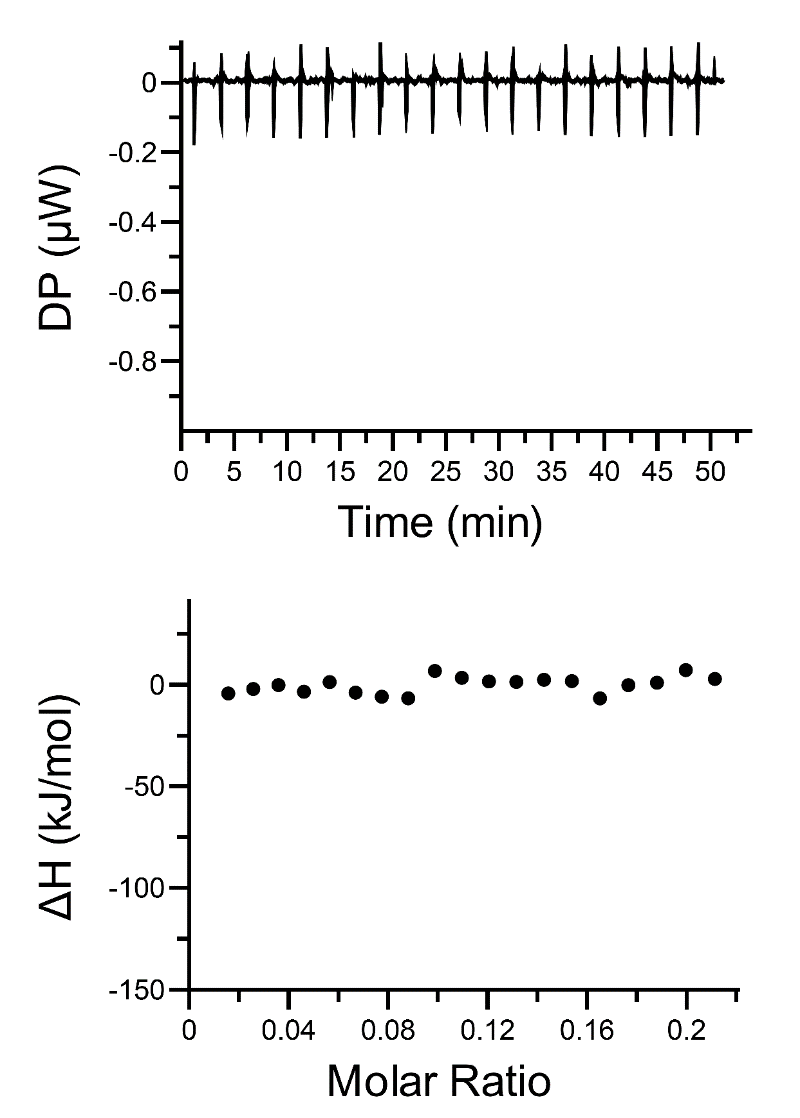


Figure S5 The control ITC measurement of titrating buffer solution (20 mM PB, pH 7.4, 50 mM NaCl) into nanodisc solution (0.75 mM total lipids).


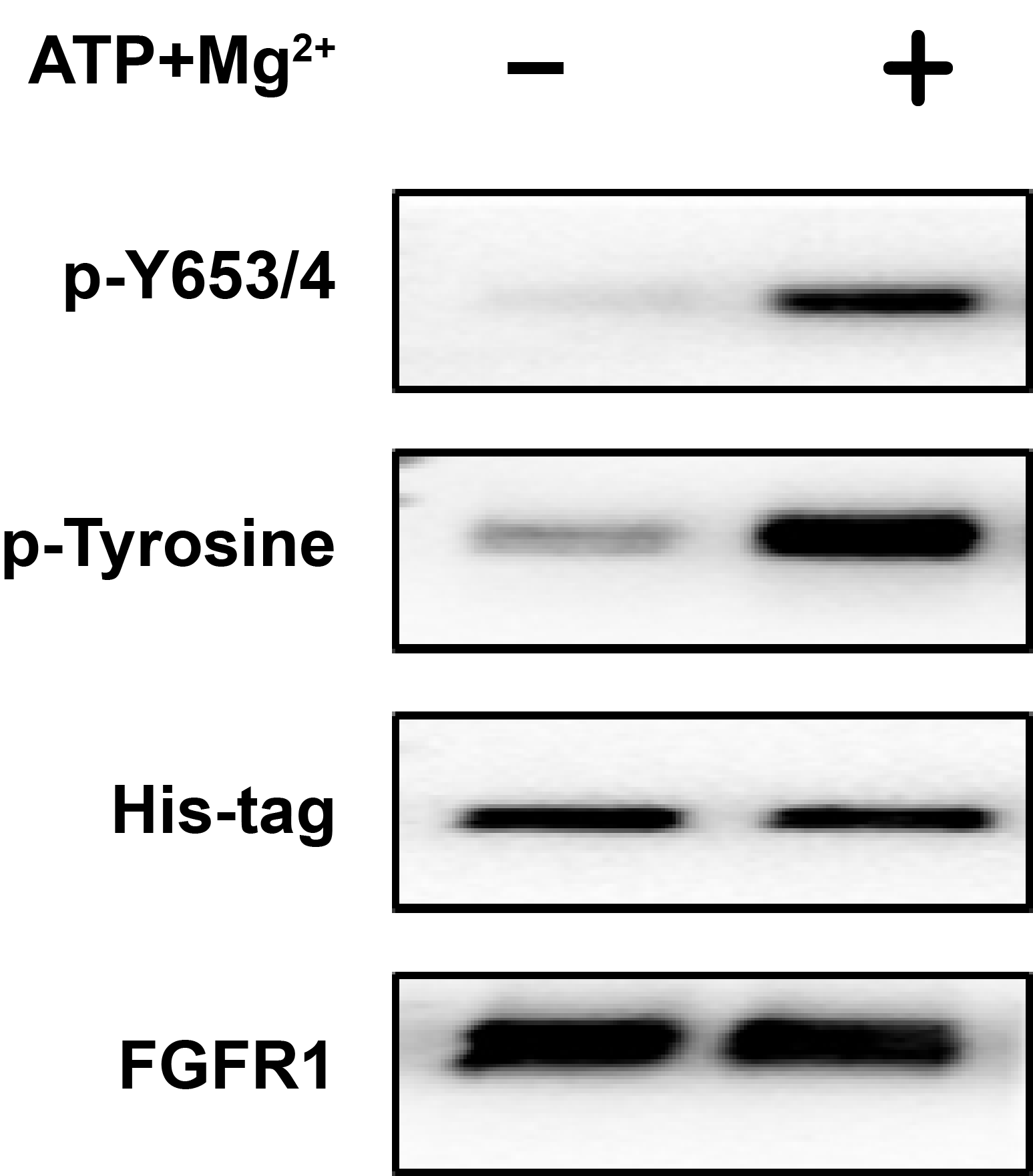


Figure S6 Enzyme activity verification of FGFR1K. The total phosphorylation (p-Tyrosine) levels and phosphorylation of Tyrosine 653/654 before (first lane) and after phosphorylation reaction for 10 minutes (second lane) were characterized by western blotting using specific antibodies. The amount of FGFR1K loaded were verified by western blotting using both His-tag antibody and FGFR1 antibody.


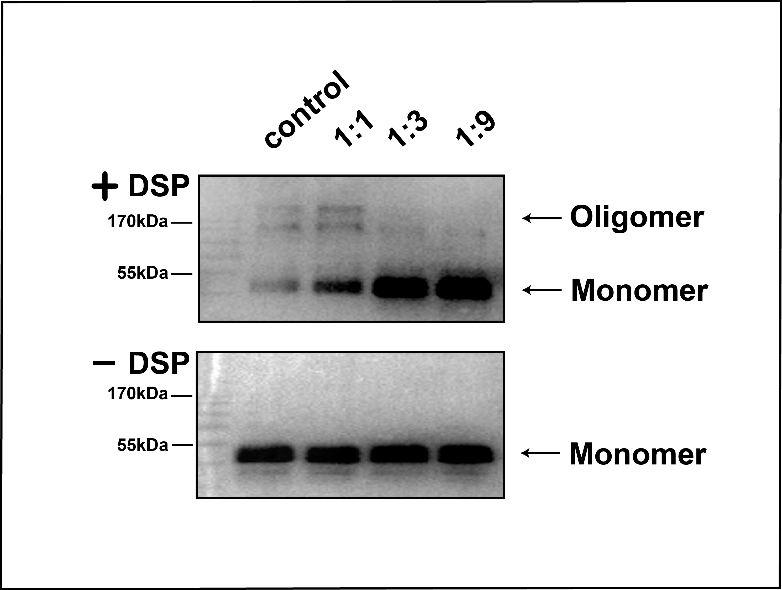


Figure S7 DSP cross-linking assay of FGFR1K in the presence of peptide nanodiscs of various sizes at lipid:peptide ratio of 1:1, 1:3 and 1:9. Cross-linking of FGFR1K in solution without nanodiscs was used as control. The amount of FGFR1K protein was semi-quantitatively characterized by western blotting analyses with an anti-His antibody.


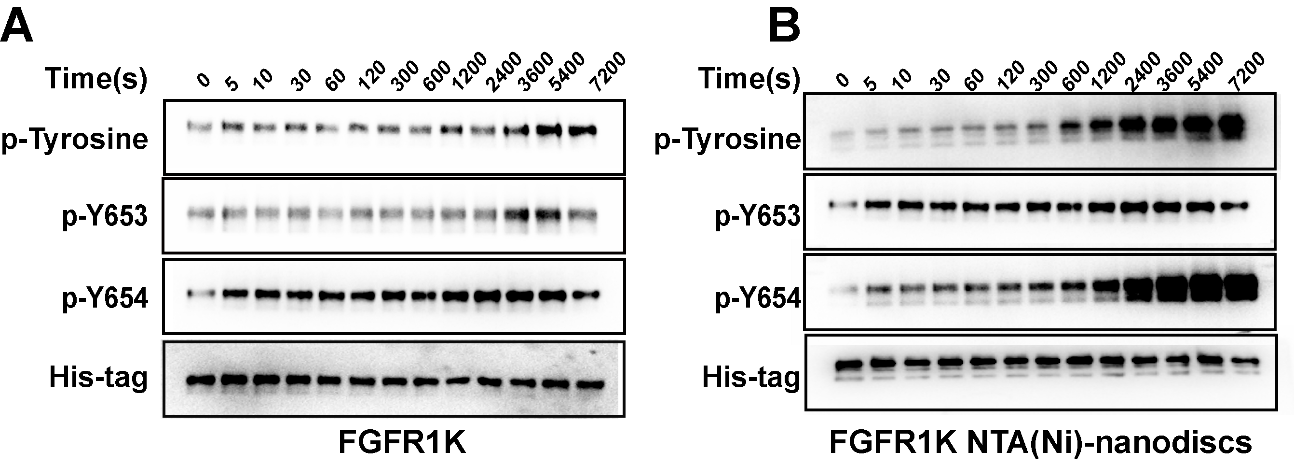


Figure S8 Western blotting analysis of FGFR1K activities in solution (A) and on peptide NTA(Ni)-nanodiscs (B) Specific antibodies of phospho-tyrosine, FGFR1 p-Y653, and FGFR1 p-Y654 were used.

**References**

Denisov IG, Grinkova YV, Lazarides AA, and Sligar SG. 2004. Directed self-assembly of monodisperse phospholipid bilayer Nanodiscs with controlled size. *J Am Chem Soc* 126:3477-3487. 10.1021/ja0393574

Hagn F, Etzkorn M, Raschle T, and Wagner G. 2013. Optimized phospholipid bilayer nanodiscs facilitate high-resolution structure determination of membrane proteins. *J Am Chem Soc* 135:1919-1925. 10.1021/ja310901f
